# Developmental Patterns of Hepatic Peroxisome Proliferator‐Activated Receptor (PPAR) Expression in *Xenopus laevis* and Response to Pharmaceutical Agonists During Metamorphic Climax

**DOI:** 10.1002/jez.70031

**Published:** 2025-09-03

**Authors:** Anna Bushong, Tyler D. Hoskins, Meredith Scherer, Maria S. Sepúlveda

**Affiliations:** ^1^ Odum School of Ecology University of Georgia Athens Georgia USA; ^2^ Savannah River Ecology Laboratory University of Georgia Aiken South Carolina USA; ^3^ Department of Forestry and Natural Resources Purdue University West Lafayette Indiana USA; ^4^ Faculty of Life Sciences Universidad Andres Bello Santiago Chile

**Keywords:** amphibian, bezafibrate, ciprofibrate, ecotoxicology, PPAR, WY‐14643

## Abstract

Peroxisome proliferator‐activated receptors (PPAR) are master transcriptional regulators that maintain metabolic homeostasis in vertebrates. Amphibians are often exposed to endocrine disrupting compounds (EDCs) that could dysregulate lipid metabolism. Larvae of the African clawed frog (*Xenopus laevis*) are routinely used as a model to study aquatic EDC exposures, but PPAR expression has not been characterized across larval development or metamorphosis in this species. We conducted two experiments to elucidate (a) the expression in late metamorphosis for xPPAR*α*/*β*/*γ* subtypes, and (b) the effect of pharmaceutical PPAR agonists (pirinixic acid, bezafibrate, and ciprofibrate) on the expression of xPPAR*α*/*β*/*γ* target genes. Additionally, we considered apical endpoints (body mass, body condition [scaled mass index, SMI], and relative liver mass). We hypothesized pharmaceuticals would agonize hepatic xPPAR*α*/*β*/*γ*, upregulating expression of downstream target genes and reducing apical endpoints with variation reflective of developmental patterns of nuclear receptor expression. We observed upregulation of xPPAR*α* during late premetamorphosis (NF 51), prometamorphosis (NF 56–57), and metamorphic climax (NF 58–66), which also held for xPPAR*γ* with exception for peak of metamorphic climax (NF 62). For xPPAR*β*, we only observed upregulation at conclusion of metamorphic climax (NF 66). Agonists did not cause changes in gene expression for xPPAR*α*/*β*/*γ* targets, but pirinixic acid exposure decreased female body condition. The dynamic hepatic expression of xPPAR*α*/*β*/*γ* during late metamorphosis is presumably necessary to coordinate energy flux and highlights a potential period of susceptibility to PPAR agonism. However, pharmaceuticals identified to interact with xPPARα/β/γ did not elicit a response concordant with PPAR agonism at high doses. These results suggest that *X. laevis* may not be a sensitive model for studies testing PPAR‐mediated effects of xenobiotics.

## Summary

- Contaminants may dysregulate lipid metabolism by altering peroxisome proliferator‐activated receptors (PPAR) signaling pathways.
- We characterized the expression of PPAR‐related genes during *X. laevis* metamorphosis.
- Aquatic exposure to the PPAR agonist pirinixic acid caused no changes in expression of hepatic target genes, but reduced female body condition.

## Graphical abstract

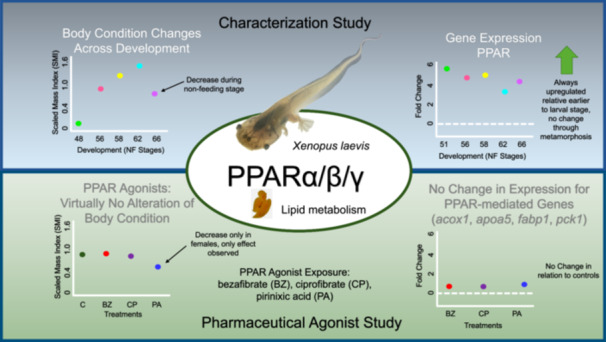

## 1 Introduction

Peroxisome proliferator‐activated receptors (PPAR) are nuclear receptors fundamental in orchestrating vertebrate tissue differentiation and development, as well as energetic coordination through regulation of lipid metabolism and energetic homeostasis. PPAR have been identified as potential targets of numerous environmental contaminants (i.e., phthalates, BPA, PFAS, organotins, etc.) (Casals‐Casas et al. 2008; Lau et al. 2010). Despite the clinical exploration for agonists to exploit PPAR to ameliorate metabolic disorders, there is concern that long‐term interference of these signaling cascades may promote metabolic disorders like dyslipidemia, hyperglycemia, and nonalcoholic fatty liver disease, which has concerning implications for the health of both humans and wildlife (Casals‐Casas et al. 2008; Nadal et al. 2017).

Given the vast number of environmental contaminants that may bind to PPAR and result in adverse effects, in vitro binding assays have served as valuable, efficient tools to screen compounds for potential bioactivity. For example, PFAS have been shown to bind to the active site of PPAR with different affinities that are substantially driven by differences in chain length and functional group (Khazaee et al. 2021). Therefore, tests that also inform in vivo physiological responses of such chemicals are of great utility due to the vast number of putative PPAR agonists. However, there is indication for species differences in the transcriptional response and ligand affinity of PPAR between rodents, humans, fish, and anurans (Gonzalez and Shah 2008; Garroche et al. 2021). For amphibians, the only data available on PPAR subtype expression is for *Xenopus laevis* with xPPAR*α/β/γ* data limited to Nieuwkoop and Faber (NF) stages 0–50 and adult organs (Faber and Nieuwkoop 1994; Bowes et al. 2010). Additionally, there is a lack of information available regarding dynamics of xPPAR*α/β/γ* expression during late premetamorphosis (NF 48–51), prometamorphosis (NF 52–57), and metamorphic climax (NF 58–66), which spans a developmentally vital period of energetic coordination as tadpoles transform into froglets. This is despite the fact that PPAR were identified decades ago, and that this anuran has long been a commonly‐used, valuable model organism for developmental biology and environmental toxicology (Dreyer et al. 1992).

The relative expression of xPPAR*α/β/γ* and effects of PPAR agonism have not been well characterized in vivo in *Xenopus laevis*. High interspecies variability on the transcriptional response and ligand affinity of PPAR supports the need to evaluate the in vivo effect of PPAR agonism for the amphibian model *X*. *laevis*. The goal of our work was twofold. First, we aimed to characterize the relative xPPAR*α/β/γ* gene expression from late premetamorphosis to metamorphic climax in the liver, which is often the focal target for toxic insult and a valuable organ for energy storage. We hypothesized transcriptional activity to be heightened for all receptors during late prometamorphosis and metamorphic climax to generally support anabolic requirements of metamorphic reorganization relative to premetamorphic stages. Second, we further tested the utility of this amphibian model to examine the effects of pharmaceutical PPAR agonists on the downstream expression of xPPARα/β/γ target genes during prometamorphosis. We hypothesized that exposure of *Xenopus* larvae to established xPPAR pharmaceutical agonists (pirinixic acid for xPPAR*α*, bezafibrate for xPPAR*β*, and ciprofibrate for xPPAR*γ*) would alter expression of associated downstream genes and lipid storage as determined through apical proxies. Additionally, we characterized scaled mass index (SMI) across metamorphosis, which can serve as a non‐destructive proxy for energetic reserves in anurans, given its correlation with lipid reserved (MacCracken and Stebbings 2012). This information may help identify developmental stages that are particularly vulnerable to suspected endocrine disruptors like PFAS, which are hypothesized to induce toxicity via PPAR‐mediated dysregulation of lipid homeostasis (Lin et al. 2022).

## 2 Methods

### 2.1 *Xenopus laevis* Acquisition and Common Garden Husbandry

Larval *Xenopus laevis* were sourced from the Great Lakes Toxicology and Ecology Division (GLTED) of the USEPA (Duluth, MN, USA for the characterization study) and from Xenopus1 Inc. (Dexter, MI, USA for the pharmaceutical study). Culturists injected 750 µL of 500 IU/mL human chorionic gonadotrophin (HGC) into the dorsal lymph sac of females. The animals from the GLTED were transported to Purdue University Entomology Environmental Laboratory (West Lafayette, IN) in temperature‐controlled coolers 48‐h post‐hatching. Upon arrival, embryos were transferred to culture water (reconstituted reverse osmosis; hereafter, RO water, to 50 mg/L CaCO_3_ hardness via addition of 9.6 mg/L sodium bicarbonate and 0.132 mL/liter Seachem Replenish^TM^ (Madison, GA), pH 7–8), with partial water changes conducted daily. At 4 days post fertilization, tadpoles were fed Sera Micron® (Sera, Heinsberg, Germany), dissolved in RO according to a graduated feeding schedule based on age (Table S.1). A single clutch of late premetamorphic tadpoles (~NF 51) was shipped overnight by Xenopus1 to Purdue University and transported to the Wildlife Ecology Research Facility (West Lafayette, IN, USA). Upon arrival, larvae were transferred to 15 L polypropylene containers with 7.5 L aged‐well water (~200 mg/L hardness, pH 7.0–8.5) at a density of 40 tadpoles per bin. Partial water changes (80%) were conducted at a minimum every 48 h. Tadpoles from Xenopus1 were also fed Sera Micron® in the same manner as those from the GLTED (Table S.1). The feeding schedule was intended to maintain ad libitum feeding and our target water parameters (dissolved oxygen, ≥ 3.5 mg/L; pH 7.0–8.5; total ammonia, ≤ 0.25 mg/L). If a scheduled feeding rate increase caused excess buildup of food from inadequate clearance, feeding levels for all units were returned to the previous bracket until clearance increased. Water chemistry was monitored using a YSI multiparameter meter (Yellow Springs, OH) to measure dissolved oxygen, and pH and total ammonia quantified using API® freshwater water test kits and test strips (Chalfont, PA, USA).

### 2.2 Chemicals, Stock, and Exposure Solutions in Agonist Study

In vitro coreceptor binding assays have shown that xPPAR bind to pharmaceutical PPAR agonists and may be capable of forming transcription complexes, indicating the potential for altering gene expression. Specifically, pirinixic acid, bezafibrate, and ciprofibrate have been identified as high affinity ligands for xPPAR*α*, xPPAR*β*, and xPPARγ, respectively (Krey et al. 1997; Desvergne et al. 1998). Pirinixic acid (i.e., WY 14,643) (PA, Batch# 0611865‐7, CAS 50892‐23‐4), Bezafibrate (BZ, Batch# 0546935‐9, CAS 41859‐67‐0), and Ciprofibrate (CP, Batch# 0474238‐10, CAS 52214‐84‐3) were obtained from Cayman Chemical (Ann Arbor, MI, USA). Each stock solution was prepared by dissolving compounds in molecular‐grade dimethyl sulfoxide (DMSO) from Sigma Aldrich (St. Louis, MO, USA) (Lot# SHBP7906, CAS 67‐68‐5) at the following concentrations: PA, 16.0 mg/mL; BZ, 17.88 mg/mL; and CP, 14.29 mg/mL. Stock solutions were prepared for each temporal block (7.3 mL, see more on this below) in two batches in 20 mL glass scintillation vials at room temperature and left on a stir plate overnight. Based upon the biodegradation half‐lives of these compounds (~3.5–4.5 days), this approach was taken to minimize pharmaceutical degradation from initiation to each water change (Williams et al. 2017). The exposure solution was prepared using aged‐well water by slowly spiking the stock solution into 500 mL water on a stir plate and adding 250 mL water for a total volume of 750 mL. This yielded four exposure solutions with a final concentration of 0.1% v/v DMSO and nominal 50 µM of the designated pharmaceutical agonist (see justification for chosen concentrations below).

### 2.3 Characterization Study

We conducted this study in the Environmental Entomology Laboratory at Purdue University. Sampling occurred across NF developmental stages starting in premetamorphosis (NF 48, NF 51), through prometamorphosis (NF 56, NF 57) and metamorphic climax (NF 58, NF 62, NF 66). Study replicates consisted of 1 L MeOH‐rinsed deli containers holding 750 mL of reconstituted RO water (made in the same manner as described for common garden husbandry) and a single *Xenopus laevis* larva.

Replicates were randomly designated for stage‐specific sampling (*n* = 18 at NF 48; *n* = 15 at NF 51, 56, 57, 58, 62, and 66) and split between two spatial blocks following a randomized block design. Once the larvae from the three clutches reached NF 46, tadpoles were pooled to homogenize genotypic variation across clutches and haphazardly added to study replicates. Sampling started in early premetamorphosis (NF 48) due to expression of xPPAR subtypes having been previously characterized through the end of premetamorphosis in whole larvae (Punzon et al. 2013; Session et al. 2016). Study units were kept at ambient water temperature (21°C ± 3°C) with a 12:12 photoperiod. Tadpoles were fed twice daily (~9:00 and ~15:00 EST) with 100% water changes every 48 h (Table S.1). Water quality was measured before each water change to check if parameters were within target values. During metamorphic climax, *X. laevis* tadpoles reach a non‐feeding stage (~NF 62) as the intestines undergo remodeling, which can be observed due to loss of continuous feeding behavior and sudden lack of food clearance (Schreiber et al. 2005). When lack of food clearance for a tadpole was observed, feeding ceased until the next water change, when larvae were fed once more to confirm their non‐feeding status.

### 2.4 Pharmaceutical Agonist Study

We conducted this experiment at the Wildlife Ecology Research Facility at Purdue University. This experiment included only a DMSO vehicle control (0.1% v/v DMSO), and three pharmaceutical compounds identified as agonists of xPPAR, pirinixic acid (50 µM), bezafibrate (50 µM), and ciprofibrate (50 µM). This dose for the pharmaceutical exposure is based on 50% of the effective dose (ED_50_) values for ligand‐xPPAR binding reported through coactivator‐dependent receptor ligand assays (CARLAs) for each compound, selecting a concentration that represents ~ED_75_‐ED_90_ (Krey et al. 1997).

Experimental replicates consisted of 1 L deli containers holding 750 mL of exposure solution and a single *X. laevis* larvae (*n* = 12 replicates/treatment). We spread replicates evenly across three temporal blocks (*n* = 4 replicates/treatment/block) following a randomized block design. Tadpoles were reared under common garden conditions until close to NF 56 by visual assessment, anesthetized using buffered MS‐222 (140 mg/L), and presorted into 15 L polypropylene container by NF stage. Tadpoles (NF 56) presorted bins were haphazardly selected to initiate the first temporal block. Temporal blocks were initiated 24‐h apart and we sourced tadpoles for subsequent blocks from the same presorted bins. The day we added tadpoles to the deli containers of the first temporal block was designated as experimental day 0 (08/21/22), and subsequently initiated the second temporal block on experimental day 1 (08/22/22), and the third temporal block on experimental day 2 (08/23/22). Tadpoles from temporal blocks were sampled sequentially on experimental days 8 (08/29/22), 9 (08/30/22), and 10 (08/31/22). A control (aged‐well water with a tadpole) was included per temporal block to monitor water quality before each water change and ensure our target water parameters were met. Study units were kept at ambient water temperature (21°C ± 1°C) and the room was set for a 12:12 photoperiod. Tadpoles were fed twice per day (~9:00 and ~15:00 EST) with a 100% water change and treatment renewal every 48 h (Table S.1).

### 2.5 Apical Data and Tissue Collection

Tadpoles were sampled after euthanasia in buffered MS‐222 (3 g/L). For the characterization study, tadpoles were collected upon reaching their predesignated NF stage for sampling. Tadpoles from the pharmaceutical study were exposed for 7 days and sampled on the 8th day according to the randomized block design for their temporal block with an average exposure time of 191 h (SD = 0.55), based on hours elapsed between the addition of the first tadpole for a temporal block to the lethal sampling of that same tadpole. NF stage, snout‐vent‐length (SVL), and wet body mass were recorded for each tadpole. Calipers were used to measure SVL with the tadpole positioned on its right side (head oriented left), with exception for premetamorphic tadpoles (characterization study) that had SVL measured from a photograph taken on a Leica Flexcam C1 microscope camera (Wetzlar, Germany) using ImageJ software. Tissues dissected from tadpoles, except NF 48 sampled as whole tadpoles, were immediately preserved upon excision. Tail clips (NF 56–62) or two‐outer digits of the right hindfoot (NF 66) were flash frozen for genotypic sexing and bisected livers were preserved in RNAlater® (Thermo Fisher Scientific, Waltham, MA, USA) for RT‐qPCR.

### 2.6 Water Chemistry

Water samples were analyzed using liquid chromatography mass spectrometry (LC/MS/MS) with an Agilent 6460 Triple Quadrupole Mass Spectrometer (Santa Clara, CA) at the Metabolite Profiling Facility of the Purdue Bindley Bioscience Center. A standard curve was constructed via a 10‐fold serial dilution (0.0–50,000 ng/mL) using water, which was used to calculate sample concentrations. Water samples were diluted 5x before LC/MS/MS analysis to ensure samples were within dynamic range of the standard curve (5.0–5000 ng/mL).

### 2.7 Gene Expression

A bisected portion of liver was stored in RNAlater®, left at ambient temperature for 48 h, then stored in −20°C until processing. Whole tadpoles (NF 48) and livers were individually extracted to obtain total RNA using Qiagen RNeasy® mini kit (Hilden, Germany) according to manufacturer protocols and recommendations for extraction from livers. Individual samples provided adequate RNA yield and purity, which were measured using a Nanodrop 8000 spectrophotometer (260/280 = 2.0 ± 0.2) before storage at −80°C. After thawing, RNA was treated for DNA contamination with Thermo Fisher Scientific DNase 1 (Waltham, MA) immediately before cDNA synthesis. For the gene expression characterization study, we used DNase‐treated RNA from NF 48 whole tadpoles (*n* = 9) and bisected livers of individual tadpoles (NF 51, 56, 57, 58, 62, 66) (*n* = 8/stage) to synthesize cDNA from 200 ng total starting material. For the pharmaceutical agonist study, we used DNase‐treated RNA from a bisected liver of single tadpoles (*n* = 12/treatment) to synthesize cDNA from 1 μg total RNA starting material. All reverse transcription was completed using Invitrogen SuperScript III reverse transcriptase (Waltham, MA, USA) per manufacturer instruction. Synthesized cDNA was diluted to a working concentration of either 4 ng/μL (characterization study) or 2.5 ng/μL (pharmaceutical agonist study) and stored at −20°C until qPCR analysis. We selected a higher working concentration of cDNA for the characterization study since we were concerned with nuclear receptors being a less abundant target transcript relative to other proteins.

**Figure 1:**
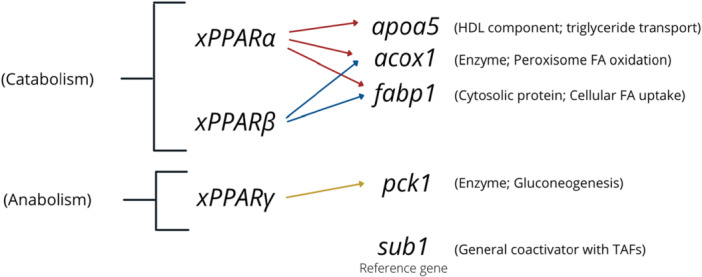
Summary of xPPAR*α*/*β*/*γ* target genes selected for RT‐qPCR analysis and the function of the encoded protein. HDL = High Density Lipoprotein, FA = Fatty Acid, TAFs = Transcription factors.

Downstream target genes (*apoa5*, *fabp1*, *acox1*, *pck1*) were selected for analysis based upon their biological function related to lipid homeostasis, namely lipid transport (*apoa5*), fatty acid transport (*fabp1*), and fatty acid oxidation (*acox1*), and the xPPAR KEGG pathway to identify targets directly downstream of xPPAR*α*/*β*/*γ* (Figure 1; KEGG 2021). Although related to gluconeogenesis, the downstream target, *pck1*, provides a downstream target specific to PPAR*γ*. As a reference gene, we selected *sub1* since it has been identified as a stable reference gene for NF 0–50 in *Xenopus laevis* for RT‐qPCR research and evaluated through a pilot assessment as suitable for our selected NF stages (Mughal et al. 2018; Table S.13).

RT‐qPCR assays were performed in technical duplicate on a Thermo Fisher Scientific QuantStudio 3 Real‐Time qPCR system (Waltham, MA, USA) with Bio‐Rad iQ SYBRTM Green SuperMix (Hercules, CA, USA). Each assay concluded with a continuous melt curve. RT‐qPCR primers for *Xenopus laevis* xPPAR*α*/*β*/*γ*, target genes of interest (*apoa5*, *fabp1*, *acox1*, *pck1*) and reference gene (*sub1*) were sourced from literature or developed using published NCBI nucleotide data, Primer3Plus software, and the UCSC genome browser (Kent et al. 2002; Sayers et al. 2022). Primer pair reaction efficiency was determined using serially diluted standard curves with cDNA derived from NF 48 whole tadpoles (Table S.2). Additional information on primer parameters and validation process can be found in the Supporting Information.

### 2.8 Genotypic Sexing

Tissue samples collected for genotypic sex determination were held at −80°C until being processed. DNA was extracted using the Qiagen DNeasy® blood and tissue kit (Hilden, Germany) according to manufacturer instructions. Genotypic sex was determined using a PCR assay on BioRad T100 Thermal Cycler with GoTaq® Green Master Mix (Madison, WI, USA) using the following thermal profile: (a) 1 cycle at 95°C for 1 min, (b) 40 cycles of 95°C for 15 s, primer‐specific annealing temperature for 30 s, 72°C for 30 s, and (c) 1 cycle of 72°C for 30 s. PCR product was visualized using gel electrophoresis to determine the presence of the DM‐W gene, which is a W‐linked gene necessary for ovary formation in *Xenopus laevis* ZW individuals (Yoshimoto et al. 2008).

## 3 Body Condition and Organ Indices

### 3.1 Scaled Mass Index

We calculated scaled mass index (SMI) as a measure of body condition according to the approach of Peig and Green (2009), which adjusts an individuals' mass to a fixed body size based on an average length measurement associated with structural size. First, mass and SVL for the reference animals (each NF stage for the characterization study; DMSO vehicle controls for the pharmaceutical agonist study) were plotted and extreme outliers excluded according to the 3xIQR criterion. A reference group is utilized to calculate the scaling coefficient to avoid skewness by a given treatment or developmental effect of interest. Next, mass and SVL were natural log‐transformed and used to perform a standard major axis regression (SMA). The slope of the best fit line was used to obtain the bSMA scaling exponent (Equation 1). This approach was also used to calculate scaled hepatic index (SHI) to report this allometrically‐scaled proxy for liver condition alongside the commonly used hepatic somatic index (HSI).

$${\hat{M}}_{i}=M_{i}{\left[\frac{L_{0}}{L_{i}}\right]}^{b_{\text{SMA}}}$$

**Equation 1**. Formula for Scaled Mass Index (SMI). “Mi” = mass of individual. “Li” = length of individual. “L0” = mean length of the reference group of animals. “bSMA” = exponent that scales the relationship to the reference group. “M^_i” = scaled mass index for a given animal (i.e., predicted body mass at the average length of the reference group).

### 3.2 Hepatic Somatic Index (HSI) and Fat Body Somatic Index (FSI)

Liver mass and fat body mass of tadpoles were expressed as a percentage of whole‐body mass to provide a relative measure for assessing developmental trends across late metamorphosis in the characterization study (Equation 2). HSI was calculated for the pharmaceutical study, but not fat body somatic index (FSI) since fat bodies were not excised.

$$\mathrm{Organ Somatic Index}=\frac{\mathrm{Whole Organ Mass}\;(g)}{\mathrm{Whole Body Mass}\;(g)}*100\%$$

**Equation 2**. Formula for Organ Somatic Index (hepatic somatic index, HSI; fat body somatic index, FSI), expressing the proportion of organ mass to body mass as a percentage.

#### 3.2.1 Statistical Analyses

All data analysis and statistics were carried out in R (R version 4.3.1; R Core Team 2019).

### 3.3 Morphometric Data

Morphological and developmental endpoints were analyzed using factorial independent analyses of variance (ANOVA) to assess either changes across life stages or pharmaceutical treatment, dependent on the study. We did not use repeated measures approaches because animals were lethally sampled. Developmental stage or pharmaceutical treatment were used as the main independent variable, and study‐specific blocking variables and genotypic sex as covariates. For post‐hoc analysis, we used Tukey contrasts to test all pairwise combinations, specifically to assess apical changes across development in the characterization study, and Dunnett contrasts to compare treatment groups or NF stages to a baseline control group, which we used for the pharmaceutical study and when performing relative gene expression analysis. Assumptions for normality and homogeneity of variance were visually assessed utilizing residual plots and, for the latter, a Levene's test. If assumptions were violated, we considered non‐parametric alternatives, such as Welch's ANOVAs with a Games‐Howells post‐hoc test if variances between groups were not equal. We defined extreme outliers using the 3xIQR criterion, excluding these experimental units before rechecking assumptions. For a given endpoint, we defined statistical significance for a developmental group or experimental treatment at α = 0.05, indicating difference from the reference group or vehicle control group.

### 3.4 Gene Expression Data

Analysis of gene expression was performed using calculated relative mRNA (i.e., fold change) for all experimental samples. Before analysis, raw Ct data underwent quality assurance/quality control to exclude unreliable values (Ct > 35). After calculating relative mRNA, the data were checked for extreme outliers as defined by the 3xIQR, grouping by treatment and gene, and excluded before rechecking assumptions. Expression of target genes were calculated using the Pfaffl method relative to the housekeeping gene sub1 (Pfaffl 2001; Equation 3). Analysis of gene expression across either developmental stage or treatment, dependent on the study, was completed using factorial independent ANOVAs on the natural log of relative mRNA with post‐hoc comparisons at a fixed threshold of *p* ≤ 0.05 for statistical significance. As with our morphometric analysis, assumptions for normality and homogeneity of variance were visually assessed utilizing plots of residuals and Levene's test.

$$\mathrm{Relative mRNA}=\frac{{\left(E_{\mathrm{target}}\right)}^{\left({\bar{\mathrm{Ct}}}_{\mathrm{RefG}}-{\bar{\mathrm{Ct}}}_{\mathrm{ExpS}}\right)}}{{\left(E_{\mathrm{housekeeping}}\right)}^{\left({\bar{\mathrm{Ct}}}_{\mathrm{RefG}}-{\bar{\mathrm{Ct}}}_{\mathrm{ExpS}}\right)}}$$

**Equation 3**. Formula for relative mRNA. “E” = converted primer efficiency of either the target gene or housekeeping gene used for normalization. “${\bar{\text{Ct}}}_{\text{RefG}}$“ = average Ct of the gene in the reference group. “${\bar{\text{Ct}}}_{\text{ExpS}}$” = the average Ct of the gene between the technical duplicates of the experimental sample.

## 4 Results

### 4.1 Characterization of Morphometric Changes Over *X. laevis* Metamorphosis

**Figure 2:**
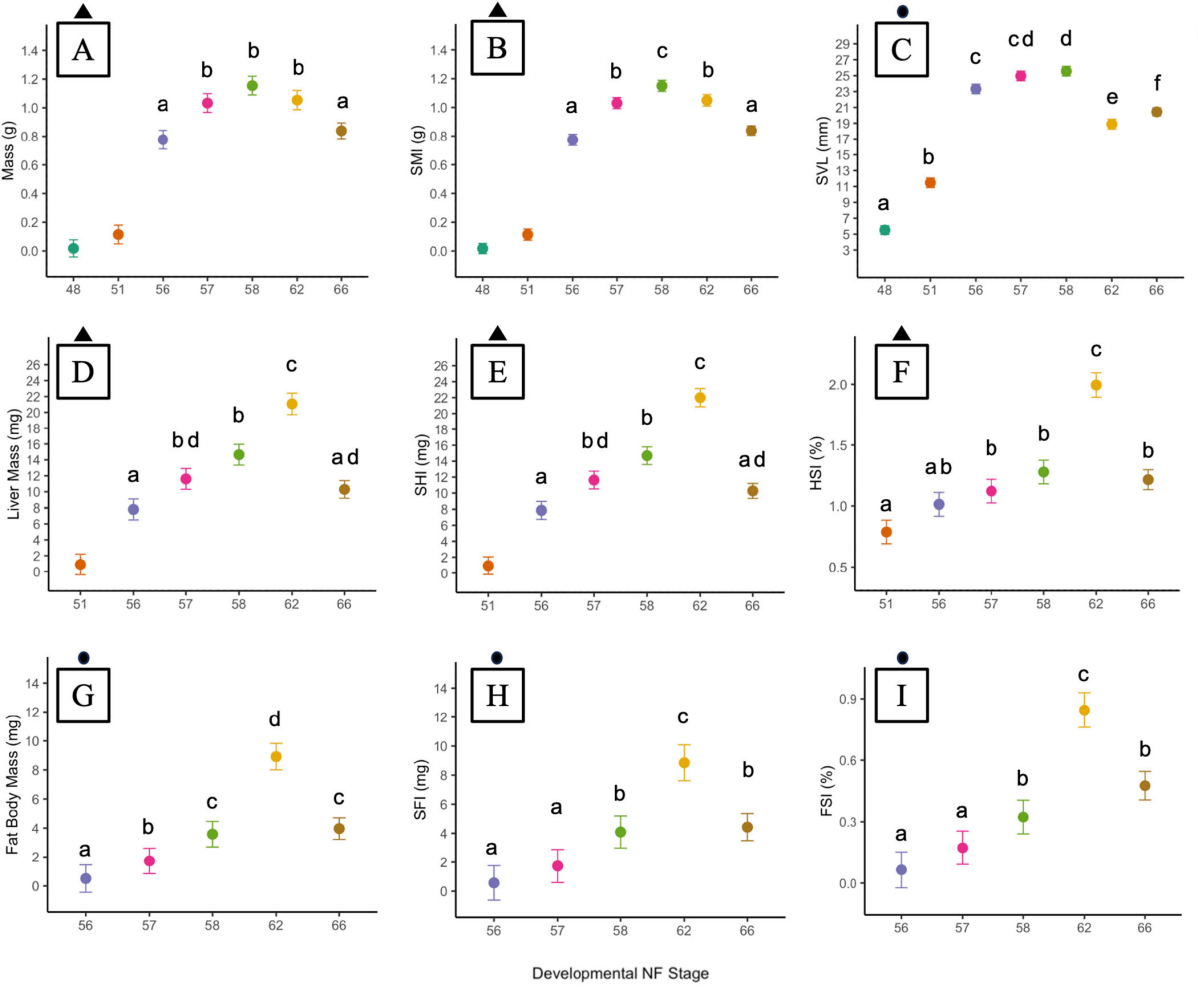
Marginal mean ± 95% CI of mass (A), Scaled Mass Index (B), Snout‐Vent‐Length (C), liver mass (D), Scaled Liver Index, SLI (E), Hepatic Somatic Index, HSI (F), fat body mass (G), Scaled Fat Body Index, SFI (H), and Fat Body somatic Index, FSI (I) of *X. laevis* across NF Stages for the characterization study. Colors represent NF Stages. Triangles denote for which plots compact letter display represents Tukey post‐hoc test at 5% threshold of significance. No letter indicates NF Stage was excluded from parametric analysis due to severe heteroskedasticity. Circles denote for which plots compact letter display represents Games‐Howells post‐hoc test at 5% threshold of significance. Marginal means not sharing any letters are statistically different. *n* = 12–19 per NF Stage.

We observed significant changes in morphometrics over focal phases of metamorphosis, namely, premetamorphosis (NF 48 and 51), prometamorphosis (NF 56 and 57), and metamorphic climax (NF 58, 62, and 66) (Figure 2). We first report analysis for whole‐body parameters and then report organ‐level trends.

A type III ANOVA accounting for spatial block and genotypic sex indicated a significant main effect of NF stage on mass and SMI (Table S.3). Post‐hoc comparisons indicate average tadpole mass increased from early prometamorphosis to late metamorphosis (NF 56–57), remained steady between late prometamorphosis to height of metamorphic climax (NF 57–62), and decreased at the conclusion of metamorphosis climax (NF 66) (Figure 2; Table S.4). However, SMI increased through prometamorphosis to peak at the beginning of metamorphic climax (NF 58) and decreased until conclusion of metamorphosis (NF 66) (Figure 2; Table S.4).

Welch's ANOVAs indicated a significant main effect of NF stage on SVL, SHI, HSI, fat body mass, SFI, and FSI (Table S.5). SVL increased from premetamorphosis to the beginning of metamorphic climax (NF 48–58), declined during the peak of metamorphic climax (NF 62), and slightly increased at the conclusion of metamorphosis (NF 66) relative to metamorphic climax (Figure 2; Table S.6). Changes in liver mass and SHI were similar across development with relative liver mass significantly increasing in late prometamorphosis (NF 57), peaking at the height of metamorphic climax (NF 62), and then significantly decreasing at the conclusion of metamorphosis (NF 66) to comparable size in late prometamorphosis (Figure 2; Table S.4). Changes in HSI followed a similar trend, with the main difference being a lack of increase in relative liver size between prometamorphic stages (NF 56–57) (Figure 2; Table S.4). SFI and FSI increased beginning in late prometamorphosis (NF 57), continuing through to peak at the height of metamorphic climax (NF 62), and decreasing at the conclusion of metamorphosis (NF 66) (Figure 2; Table S.6).

### 4.2 Characterization of xPPAR Expression Over *X. laevis* Metamorphosis

**Figure 3:**
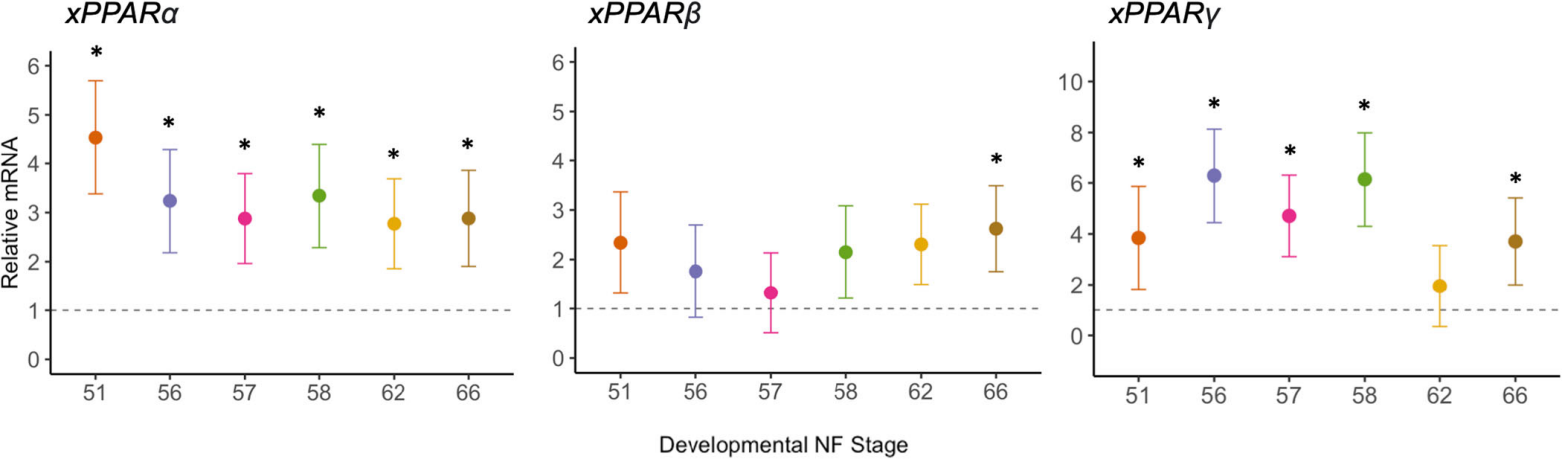
Marginal mean ± 95% CI of liver x*PPARα*, x*PPARβ*, and x*PPARγ* relative mRNA (i.e., fold change) for *X. laevis* (NF 51‐66) relative to whole animal NF 48 expression represented by a dash line at 1. Dots represent treatment marginal mean ± 95% C.I. Asterisks (*) indicate the marginal mean was significantly different from NF 48 (*p* < 0.05) from a Dunnett post‐hoc test. *n* = 5–8 per NF stage.

Hepatic expression of xPPAR*α*, xPPAR*β*, and xPPAR*γ* varied significantly across development (type III ANOVA) (Figure 3; Tables S.7, S.8, S.9). We did not include genotypic sex as a covariate for relative gene expression due to few genotypic males across NF stages (1 ≤ n ≤ 3). xPPAR*α* expression was upregulated relative to early premetamorphosis (NF 48) and showed highest expression at NF 51 (4.5‐fold change, FC) (Figure 3; Tables S.7, S.8). xPPAR*β* expression was significantly upregulated at the conclusion of metamorphosis (NF 66) (2.6 FC) relative to NF 48, but not during earlier stages (NF 51–62). With exception of NF 62, xPPAR*γ* expression was significantly upregulated relative to premetamorphosis, with a highest expression observed for NF 56 (6.3 FC) and NF 58 (6.1 FC).

## 5 Pharmaceutical Agonist Study

### 5.1 Water Chemistry

**Figure 4:**
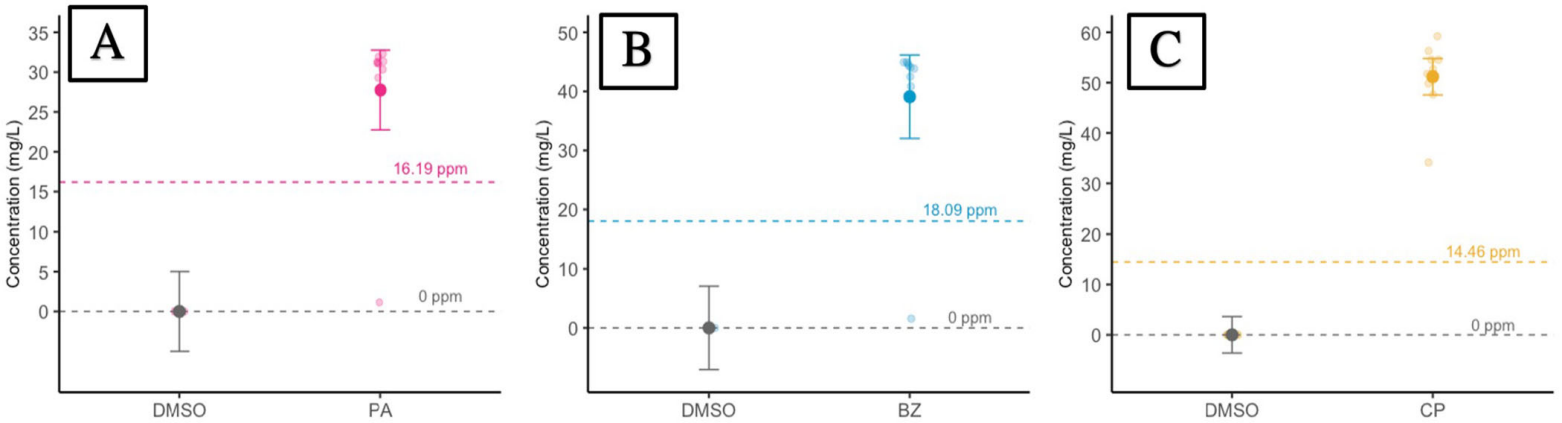
Marginal mean ± 95% CI of concentrations of pirinixic acid PA (A), bezafibrate BZ (B), and ciprofibrate CP (C) using LC/MS/MS. Panels A–C represent concentrations in exposure solution from pooled water samples collected at the initiation of each temporal block (*n* = 9) taken immediately after addition of the tadpole. Dashed line represents nominal concentration for exposure solution (A–C).

Measured concentrations of agonists pirinixic acid, bezafibrate, and ciprofibrate in water samples from the first temporal block were all above nominal (~70%–230%), after adjusting for injection volume (Figure 4; Table 1). Each treatment had an outlier sample that measured substantially lower than others and these samples represent the first batch processed for LC‐MS/MS. Therefore, we believe these outliers arose from an error in homogenization before injection.

**Table 1:** Mean concentrations of agonists (pirinixic acid, bezafibrate, ciprofibrate) using LC/MS/MS.

Sample type	Treatment	Nominal mean [Treatment] (mg/L, ppm)	Measured mean [Treatment] (mg/L, ppm)	Measured mean [Control] (mg/L, ppm)	Treatment SD	Treatment SD^	Percent difference
Exposure solution	Pirinixic acid	16.19	27.75	0	10.02	0.94	71.41%
	Bezafibrate	18.09	39.09	0.005	14.13	1.43	116.07%
	Ciprofibrate	14.46	51.18	0.008	7.25	3.66	253.94%

*Note:* Exposure solution concentrations represent the mean of pooled water samples collected at the initiation of each temporal block (*n* = 9). “SD” = standard deviation of mean treatment concentration. “SD^” = standard deviation if outlier shown in Figure 4 is excluded.

### 5.2 Effect of Acute xPPAR Agonist Exposure on Phenotypic Endpoints of *X. laevis* at Metamorphic Climax

**Figure 5:**
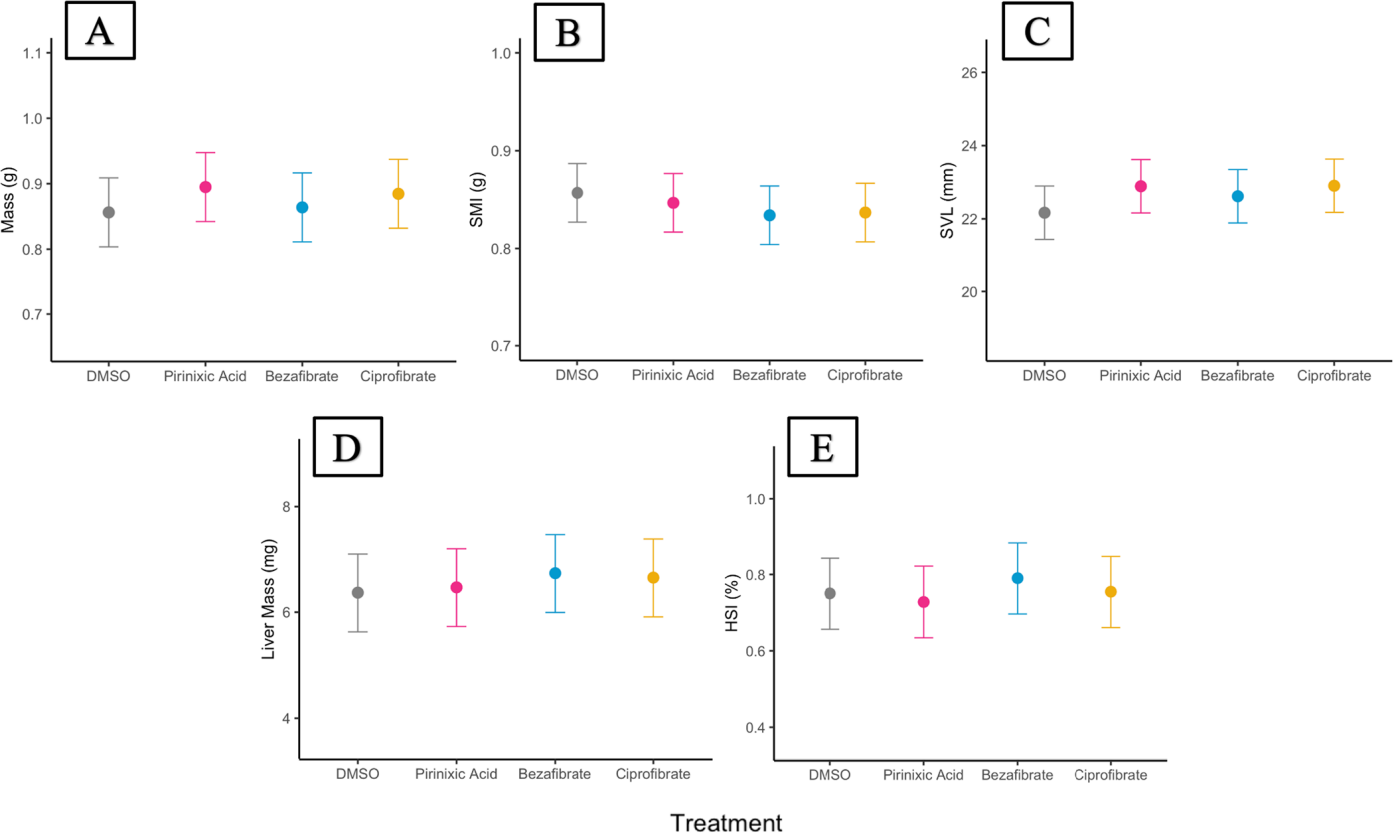
Marginal mean ± 95% CI of mass (A), SMI (B), SVL (C), liver mass (D), and HSI (E) for *X. laevis* exposed to a DMSO vehicle control or a xPPAR agonist (pirinixic acid, bezafibrate, ciprofibrate). *n* = 11–12 per treatment.

**Figure 6:**
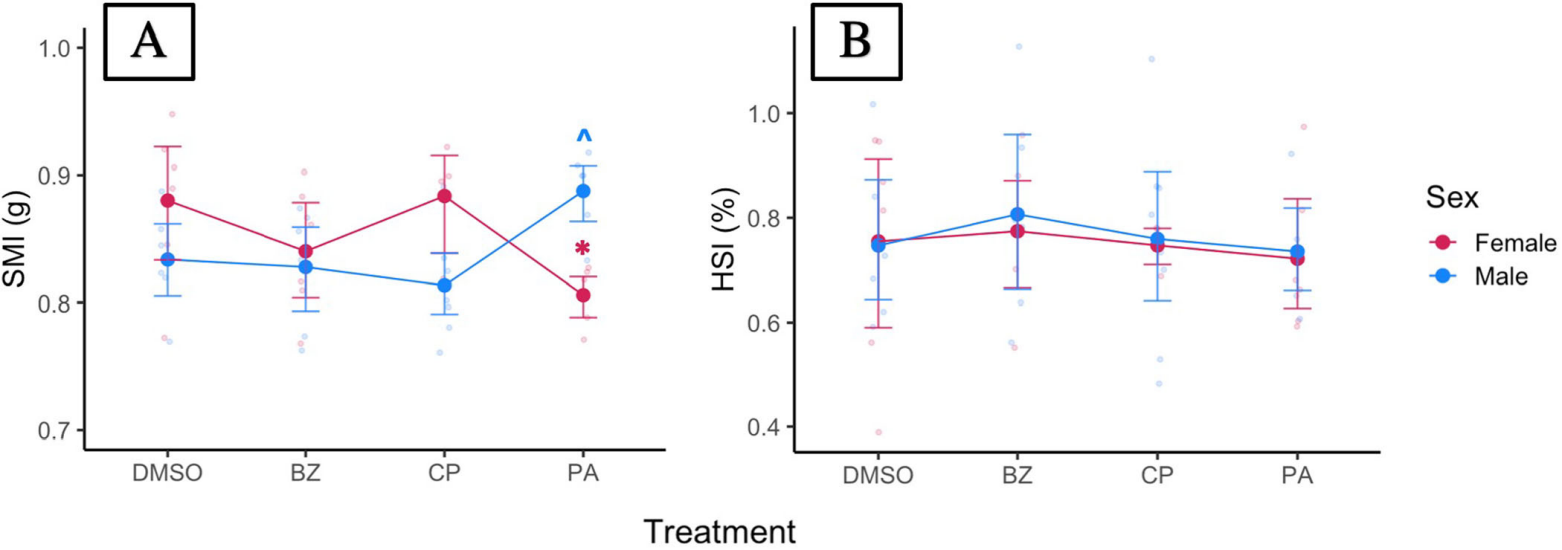
Interaction plot between genotypic sex and treatment means ± 95% CI for Scaled Mass Index, SMI (A) or Hepatic Somatic Index, HSI (B) for *X. laevis* exposed to either a DMSO vehicle control or an agonist (bezafibrate [BZ], ciprofibrate [CP], pirinixic acid [PA]) with raw data displayed in the background. Dots represent treatment mean ± 95% CI “*” indicates a significant (*p* < 0.05) and “^” indicates a weak (*p* < 0.1) treatment effect from a Dunnett post‐hoc test. *n* = 12 per treatment (n_female_ = 4–6/treatment, n_male_ = 6–8/treatment).

We did not observe effects of xPPAR agonist exposure on morphometric endpoints (Figure 5), with exception of a sex‐specific effect on SMI (Figure 6; Table S.9). We investigated the direction of this interaction and found a significant main effect of the agonist on SMI for males and females (Table S.10) with a decrease in females (8.5%) and weak increase in males (6.5%) (Table S.11). However, despite there being a sex‐specific effect on SMI, there was no interaction for HSI. We do not report SHI for this study due to the inability to establish organ‐level relationships with structural size based on the DMSO vehicle control, potentially related to variability from the temporal block.

### 5.3 Effect of Acute xPPAR Agonist Exposure on *X. laevis* Gene Expression During Metamorphic Climax

**Figure 7:**
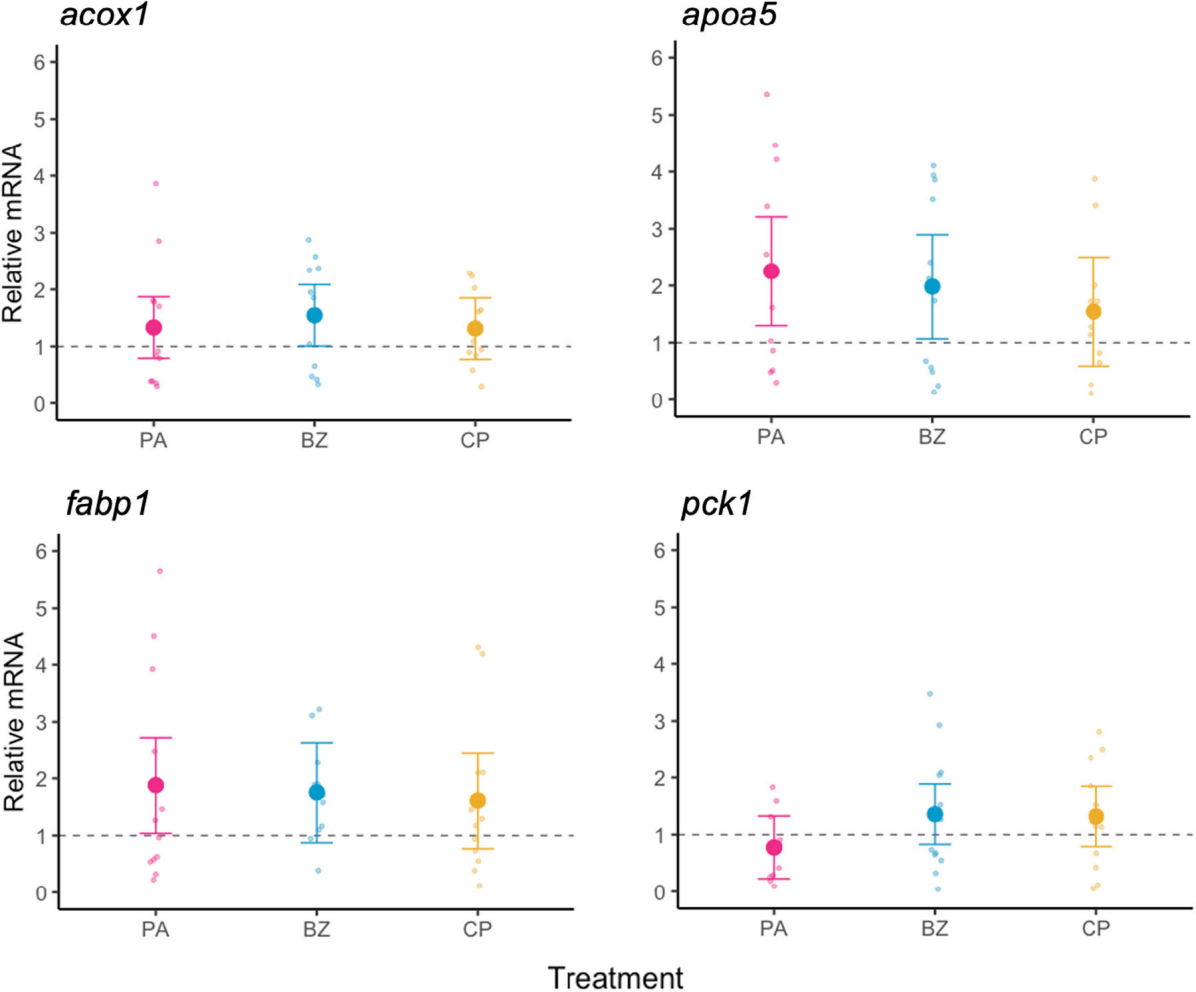
Marginal mean ± 95% CI of xPPAR target genes (*acox1*, *apoa5*, *fabp1*, *pck1*) expression in livers of *X. laevis* exposed to a xPPAR agonist (pirinixic acid, PA; bezafibrate, BZ; ciprofibrate, CP). Values relative to DMSO vehicle controls represented by the dashed line at 1.0. Raw data displayed in the background. *n* = 11–12 per treatment.

The expression of *acox1*, *apoa5*, *fabp1*, and *pck1* did not significantly change across agonist treatments relative to the DMSO vehicle control (Figure 7; Table S.12).

## 6 Discussion

### 6.1 Characterization Study

Assessing the patterns of *Xenopus laevis* body condition and *xPPAR* expression is valuable for contributing to the understanding of energetic flux and demands through anuran metamorphosis. Over the course of late premetamorphosis, prometamorphosis, and metamorphic climax, we observed a distinct rise and fall of SMI, our proxy for body condition. Notably, SMI does mirror the trend observed for mass across metamorphosis, traditionally used as an apical endpoint in toxicology studies. However, the allometric scaling of this metric reduces variability by accounting for changes in SVL during remodeling stages NF 62 through 66. SMI increases up to NF 58, as tadpoles are continuously feeding before the height of metamorphic climax due to a lack of negative regulatory feedback mechanisms; (Bender et al. 2018). This enables maximization of their body size and energy reserves to meet nutrient needs for remodeling and biosynthesis during the later non‐feeding stages. The trend of decline in SMI from NF 58 to the conclusion of metamorphosis at NF 66 aligns with the start of the non‐feeding period (~NF 62), accompanied by dramatic cranial restructuring and tail resorption.

From the perspective of developmental toxicology, evaluating differential expression of nuclear receptors is important to identify critical phases when an organism may be particularly susceptible to xenobiotic insult. When evaluating expression relative to early premetamorphosis (NF 48), hepatic *xPPARα/β/γ* showed distinct patterns. PPARα is hailed as the master regulator of hepatic lipid metabolism that maintains metabolic flexibility by adapting lipid catabolism and storage to maintain homeostasis with its activation generally enhancing fatty acid oxidation (Bougarne et al. 2018; Berthier et al. 2021). During late premetamorphosis, prometamorphosis, and metamorphic climax, *xPPARα* was significantly upregulated relative to early premetamorphosis with the highest expression observed during late premetamorphosis (NF 51) and then stabilizing until metamorphic climax. This pattern deviates from observations in *Rana omeimontis* where there was a downregulation of hepatic *xPPARα* at metamorphic climax compared to prometamorphic tadpoles (Zhu et al. 2020). This difference in hepatic transcription of *xPPARα* could relate to differences in species metabolic adjustments and regulation during metamorphosis. Unlike *X. laevis*, *R. omeimontis* lacks larval fat bodies and may downregulate catabolism during metamorphic climax to conserve hepatic lipid reserves until metabolic contributions of tail resorption have been exhausted (Zhu et al. 2019; Zhu et al. 2020).

*PPARβ* expression and functional differentiation is not extensively understood, but mammalian models indicate roles in enhancing fatty acid oxidation and glucose catabolism, working synergistically with PPAR*α* (Berthier et al. 2021). Although x*PPARα* showed transcriptional upregulation and stability in the liver of *X. laevis*, x*PPARβ* expression did not significantly differ from early premetamorphosis until it was upregulated at NF 66. Working with xPPAR*α* to direct intracellular lipid mobilization, the increased expression in x*PPARβ* could indicate higher energetic demand on the liver towards the end of metamorphosis due to depletion of other energy reserves, such as the tail and fat bodies (Wright et al. 2011; Zhu et al. 2019).

In vertebrates, *PPARγ* is expressed at lower levels than *PPARα*, but still plays an important role in mediating hepatic fatty acid uptake and promoting lipogenesis (Berthier et al. 2021). x*PPARγ* expression was upregulated through late premetamorphosis, prometamorphosis, and metamorphic climax relative to early premetamorphosis, except for NF 62. This dip in receptor expression at NF 62 aligns with the onset of the non‐feeding stage, which would inherently decrease the demand for *de novo* lipogenesis and fatty acid (FA) uptake due to the fasting state. Upregulation at the conclusion of metamorphosis (NF 66) could indicate a preparation for hepatic lipogenesis to resume as the froglet begins hunting.

### 6.2 Pharmaceutical Agonist Study

Contrary to initial expectations, we did not observe changes in expression for downstream genes of xPPARα/β/γ (*acox1*, *apoa5*, *fabp1*, *pck1*), or for most of our apical endpoints (mass, SVL, liver mass, HSI) following subchronic exposure to pirinixic acid, bezafibrate, or ciprofibrate at a nominal concentration of 50 μM. However, we did detect a sex‐specific response for body condition (SMI) to pirinixic acid exposure, where SMI decreased for female tadpoles and weakly increased for male tadpoles. Throughout this developmental period, sexually‐dimorphic differences in body condition would not be expected based upon the findings of our characterization study. However, it is important to highlight that this significant change in SMI was detected under subchronic exposure at a high pharmaceutical exposure concentration, with measured concentrations being substantially higher than the targeted nominal of 50 uM at study initiation (Figure 4, Table 1). This disparity may be related to preparing a higher stock concentration than intended at study initiation. Therefore, subsequent studies would be needed to assess whether this sex‐specific alteration elicited by pirinixic acid is detectable at lower exposure concentrations. Melvin (2016) and Melvin et al. (2017) observed apical and metabolic changes in the striped marsh frog (*Limnodynastes peronii*) following aquatic exposure to nonsteroidal pharmaceuticals and complex pharmaceutical mixtures at dose ranges well below this present study (≤ 1 ppm), indicating the ability to apically detect pharmaceutical‐mediated responses in other anurans and at lower concentrations.

Despite debate in the ecology literature regarding the appropriateness of body condition indices as proxy for energy reserves or as a fitness‐related trait, SMI has been positively correlated with energy and fat reserves in amphibians (e.g., bullfrog [*Rana catesbeiana*] and rough‐skinned newts [*Taricha granulosa*]), which are both vital components of fitness (Scott et al. 2007; MacCracken and Stebbings 2012; Wilder et al. 2016). Therefore, the decrease in SMI for female and potential increase for male tadpoles may indicate diverging energetic consequences of pirinixic acid exposures between sexes, potentially due to different toxicokinetics or focal mechanisms of action related to receptor expression or sensitivity not captured in our study. Importantly, although pirinixic acid was developed as an antihyperlipidemic agent characterized to agonize PPAR*α* in mammalian models, studies with teleost and other anurans have shown variable responses for apical endpoints in comparison. Generally, rodents exposed to high concentrations of pirinixic acid through food experience weight loss and liver enlargement (Moya‐Camarena et al. 1999; Cunningham 2007). Recent in vivo studies indicate no change in length, mass, or HSI for male Atlantic cod (*Gadus morhua)* subchronically exposed to high doses of pirinixic acid through intraperitoneal injection, and no change in length or mass for polar cod (*Boreogadus saida*) (Vieweg et al. 2018; Eide et al. 2023). For anurans, Lin et al. (2022) reported an increase in HSI and hepatic triglyceride content following aquatic pirinixic acid exposure in adult male black‐spotted frog (*Pelophylax nigromaculata*), while Zhu et al. (2021) observed an increased growth rate and hepatic lipid mobilization for *R. omeimontis*.

Importantly, these nonmammalian studies with divergent results use different routes of exposure (e.g., intraperitoneal vs. aqueous exposure with DMSO vehicle), which brings up the role of first‐pass metabolism for orally ingested fibrates (e.g., bezafibrate, ciprofibrate, pirinixic acid). In mammals, generally, fibrates are readily absorbed by the gastrointestinal system, subsequently metabolized by hepatic cytochrome P450 (CYP) 3A4, and excreted renally (Miller and Spence 1998). Notably, a developmental change in hepatic CYP3A4‐like activity has been observed in a sister species, *Xenopus tropicalis*, during metamorphic climax using a Luciferin‐IPA assay (Mori et al. 2017). In conjunction with an ortholog identified in the *X. laevis* genome and recent demonstration that phase I and II enzymes are inducible in late prometamorphic tadpoles, it is plausible that *X. laevis* tadpoles were able to compensate for exposure to high‐dose fibrates with exception for pirinixic acid (Fisher et al. 2023; Wada et al. 2024). Additionally, once systemic, fibrates strongly bind to serum albumin for transport through the bloodstream and serum proteins have classically been recognized to be less abundant in fully aquatic larvae of *X. laevis* relative to terrestrial organisms, which may contribute to a lower responsiveness (Miller and Spence 1998; Liberi et al. 2022; Duellman and Trueb 1986). Therefore, if there were sex‐specific differences in the capacity for first‐pass metabolism of pirinixic acid, then this could amount to different apical outcomes, especially given additional potential burden of exposure through gills and skin. To our best knowledge, there have been no reports on sex‐differences in anurans for apical endpoints to pirinixic acid exposure. Sexually‐dimorphic change in body mass in response to fibrate exposure has been reported in mice, but with males decreasing and females increasing in body weight, which is opposite in directionality relative to our findings (Yoon 2010). Considering these species differences and a lack of sex‐specific mechanistic insight for anurans, further studies are warranted to clarify what may be driving this sex‐specific effect of pirinixic acid on *X. laevis* body condition, particularly since our study is not able to distinguish between overt toxicity resulting from high doses and compensatory mechanisms.

There are a few potential explanations for the lack of xPPAR‐mediated transcriptional response in *Xenopus laevis* tadpoles to pharmaceutical agonists. First, despite high exposure levels, internal concentrations may not have reached effective levels if bioavailability of these pharmaceuticals was low or first‐past metabolism, as discussed, was significant; measuring hepatic levels of agonists in future studies could address this. It is also plausible that binding between an agonist and its corresponding xPPAR did not elicit alterations to transcription for this pathway in vivo, despite in vitro CARLA assays indicating cofactor recruitment (Krey et al. 1997). Our sampling regime also may not have captured transient agonistic responses during the subchronic exposure due to compensation by cellular desensitization, which could occur through nuclear receptor downregulation or repression (Santos et al. 2011). If an effective dose was reached internally, this latter explanation is reasonable given indication for potential acclimation to pirinixic acid in chronically exposed juvenile turbot (*Scophthalmus maximus*) through transitory change in the hepatic expression of the PPAR*α* target *acox1*, and bezafibrate in zebrafish (*Danio rerio*) through transitory changes in *PPARβ* expression observed in the testis (Velasco‐Santamaría et al. 2011; Urbatzka et al. 2015). However, variability of in vivo and in vitro results for xPPAR‐mediated effects of these pharmaceuticals in mammalian and aquatic species highlights the challenges of interpreting disparate results between exposure systems, across dose‐ranges, and across species (Olivares‐Rubio and Vega‐López 2016; Melvin et al. 2017; Li et al. 2018).

Overall, under this exposure paradigm, pharmaceutical agonists (pirinixic acid, bezafibrate, ciprofibrate) do not provide transcriptional insight for in vivo agonism of xPPAR pathways in late metamorphic *X. laevis* tadpoles, which did not support our hypothesis. Before attempting to use these pharmaceuticals for future in vivo exposures investigating effects of xPPAR activation in amphibians, further studies are necessary to assess the bioavailability of these compounds from aquatic exposure through quantifying body burdens and should potentially consider other delivery methods, such as intraperitoneal injection or gavage. Without this knowledge, these compounds should not be relied upon as positive controls in future studies investigating the effects of xPPAR activation in *X. laevis* in vivo.

## 7 Conclusions

There is a need for the development of novel approaches for studying the health impacts of environmental pollutants that can interact with the PPAR signaling pathway. PPARs are nuclear hormone receptors that are activated by fatty acids and their derivatives and play a vital role in energy homeostasis, including lipid metabolism. We hypothesized the pharmaceuticals PPAR agonists (pirinixic acid, bezafibrate, and ciprofibrate) would agonize *Xenopus laevis* hepatic xPPARα/β/γ, dysregulating the expression of downstream target genes (*apoa5*, *fabp1*, *acox1*, *pck1*) and reducing apical endpoints (body mass, body condition [scaled mass index, SMI], and relative liver mass). To better design and interpret the results of this experiment, we first ran a characterization study and examined temporal changes in gene expression and apical endpoints during metamorphosis which was absent from the literature. From this study we found a dynamic hepatic expression of *xPPARα/β/γ* during late metamorphosis presumably necessary in coordinating energy flux and highlighting a potential period of susceptibility to chemicals that disrupt lipid homeostasis. Apical endpoints generally followed a similar trend, with a steady increase through early prometamorphosis to late metamorphosis (NF 56–57), a height during late prometamorphosis and metamorphic climax (NF 57–62), and a decrease at the conclusion of metamorphosis (NF 66). Although not previously demonstrated, this was expected, as energy demands dramatically increase until metamorphic climax when feeding ceases. Upon exposure, however, we did not observe changes in hepatic gene expression for *xPPARα/β/γ* targets but observed a decrease in female body condition following pirinixic acid exposure. These results raise questions about how to best utilize *X. laevis* as in vivo models for researching PPAR signaling pathways. On one hand, changes in apical endpoints across development were consistent with expected patterns of fat storage before metamorphosis and fat mobilization during non‐feeding stages, suggesting that these endpoints could be useful in assessing apical effects of exposures that alter lipid homeostasis. On the other hand, the lack of responses to known PPAR agonists raises the possibility that *X. laevis* are not particularly sensitive to exogenous agonism at these developmental stages and under the conditions tested.

## Author Contributions

**Anna Bushong:** conceptualization, data curation, formal analysis, investigation, methodology, project administration, visualization, writing – original draft, writing – review and editing. **Tyler D. Hoskins:** conceptualization, methodology, resources, validation, visualization, writing – review and editing. **Meredith Scherer:** investigation, validation, writing – review and editing. **Maria S. Sepúlveda:** conceptualization, funding acquisition, methodology, resources, supervision, validation, writing – review and editing.

## Disclosure

This report was prepared as an account of work sponsored by an agency of the United States Government. Neither the United States Government nor any agency thereof, nor any of their employees, makes any warranty, express or implied, or assumes any legal liability or responsibility for the accuracy, completeness or usefulness of any information, apparatus, product, or process disclosed, or represents that its use would not infringe privately owned rights. Reference herein to any specific commercial product, process, or service by trade name, trademark, manufacturer, or otherwise does not necessarily constitute or imply its endorsement, recommendation, or favoring by the United States Government.

## Conflicts of Interest

The authors declare no conflicts of interest.

## Supporting information

General_Supplementary_Information.

Supplementary_Information_Primer_FINAL.

## Data Availability

The data that support the findings of this study are available on request from the corresponding author. The data are not publicly available due to privacy or ethical restrictions.
