# Supplementary material for: Developmental Patterns of Hepatic Peroxisome Proliferator‐Activated Receptor (PPAR) Expression in Xenopus laevis and Response to Pharmaceutical Agonists During Metamorphic Climax
Source: J Exp Zool A Ecol Integr Physiol. 2025 Sep 3;343(10):1191–204. doi: 10.1002/jez.70031 (PMC12604685; doi:10.1002/jez.70031)
Supplement: Supplementary file 1 — General_Supplementary_Information. [file JEZ-343-1191-s002.docx]

## **Table S.1.** Feeding rates adapted from the OECD AMA guidelines for static renewal (OECD, 2009). Sera Micron® was suspended in RO water to administer during feeding.

##

| **Days Post-Fertilization** | **Food Ration (mg of Sera Micron per animal/day)** |
| --- | --- |
| 4-8 | 5 |
| 9-12 | 9 |
| 13-17 | 13 |
| 18-22 | 15 |
| 23-25 | 18 |
| 26-28 | 20 |
| 29-32 | 25 |
| 33-39 | 28 |

## **Table S.2.** Information on RT-qPCR primers used for real-time quantitative PCR of *X. laevis* xPPARα/β/γ and target genes (*apoa5, fabp1, acox1,​ pck1*) and reference gene (*sub1*). ‘T_A_’ = Annealing Temperature; ‘E%’ = Efficiency calculated from slope of standard curve; ‘[Primer]’ = Concentration of each primer (F & R) per qPCR reaction. “*” denotes efficiency within dynamic range.

| **Target Gene** | **F/Left Primer (5’-3’)** | **R/Right Primer (5’-3’)** | **T_A_ (^o^C)** | **E%** | **[Primer]** | **Amplicon** | **Spans Exon?** | **NCBI** | **Source** | **DOI** |
| --- | --- | --- | --- | --- | --- | --- | --- | --- | --- | --- |
| ***acox*** | ACATGGGATCAGCAAGAACAC | CCACTGCATTAGGACGGATTA | 51.9 | 92.1 | 250 nM | 127 bp | Yes | NM_001096680.1 | Tamaoki et al., 2018 | https://doi.org/10.1002/jez.2246 |
| ***apoa*** | CCGGAAGAGTTAAGGAAGGTTT | GCTCAGCTTATCCTGTGTTGTG | 51.9 | 90.5 | 250 nM | 125 bp | No | NM_001110744.1 | Self-designed | NA |
| ***fabp1*** | TAAAGGGTGTCACCGAGATTG | TCTCCCCTGTTGGTGTTTCTA | 51.9 | 92.3 | 188 nM | 129 bp | No | AF068301.1 | Tamaoki et al., 2018 | https://doi.org/10.1002/jez.2246 |
| ***pck1*** | GCAGCTGAACATAAAGGCAAG | TGAGCCAGTGAGCAAGGTATT | 53 | 92.7 | 250 nM | 97 bp | Yes | NM_001086477.1 | Tamaoki et al., 2018 | https://doi.org/10.1002/jez.2246 |
| ***xPPARa*** | GCCGAGAAGACGTTAGTCGC | GTGAAGCCGGGGATGGATTT | 52.7 | 96.2* | 250 nM | 146 bp | No | NM_001095362.1 | Self-designed | NA |
| ***xPPARb*** | GCTTCGGCACACTCTACCTC | AAGACTGCGCAGGAACTCAC | 52 | 107.6 | 250 nM | 155 bp | No | NM_001087841.1 | Self-designed | NA |
| ***xPPARg*** | CGTCACCCCCTTACTTTTCA | CTTCTTGTGAATCCGGCAAT | 48.7 | 93.3 | 125 nM | 222 bp | Yes | NM_001087843.1 | Self-designed | NA |
| ***sub1*** | AGCAGGAGAAATGAAGCCAGG | CCGACATCTGCTCCTTCAGT | 53.5 | 97.3 | 188 nM | 80 bp | Yes | XM_018266540.2 | Mughal et al., 2018 | https://doi.org/10.1038/s41598-017-18684-1 |

## **Table S.3.** ANOVA tables (Type III) reporting comparison of mean Mass, Scaled Mass Index SMI, Liver Mass, Scaled Hepatic Index SHI, and Hepatosomatic Index HSI across NF stages, including variance of spatial block and genetic sex. Significance definitions: ‘ *** ’ $\leq$ 0.001; ‘ ** ’ $\leq$ 0.01; ‘ * ’ $\leq$ 0.05; ‘ . ’ $\leq$0.1; ns = not significant.

| **Panel** | **Response** | **Source of Variance** | **SS** | **df** | ***F*** | ***p*** | **Sig** |
| --- | --- | --- | --- | --- | --- | --- | --- |
| A | **Mass** | (Intercept) | 39.069 | 1 | 1792.133 | <0.001 | *** |
|  |  | Block | 0.099 | 1 | 4.533 | 0.037 | * |
|  |  | Sex | 0.005 | 1 | 0.225 | 0.637 | ns |
|  |  | NF Stage | 1.013 | 4 | 11.622 | <0.001 | *** |
|  |  | Sex:NF Stage | 0.010 | 4 | 0.112 | 0.978 | ns |
|  |  | Residuals | 1.439 | 66 |  |  |  |
| B | **SMI** | (Intercept) | 37.981 | 1 | 4709.478 | <0.001 | *** |
|  |  | Block | 0.023 | 1 | 2.828 | 0.097 | . |
|  |  | Sex | 0.022 | 1 | 2.671 | 0.107 | ns |
|  |  | NF Stage | 0.994 | 4 | 30.818 | <0.001 | *** |
|  |  | Sex:NF Stage | 0.026 | 4 | 0.802 | 0.528 | ns |
|  |  | Residuals | 0.540 | 67 |  |  |  |
| D | **Liver Mass** | (Intercept) | 7225.440 | 1 | 939.111 | <0.001 | *** |
|  |  | Block | 37.413 | 1 | 4.863 | 0.031 | *** |
|  |  | Sex | 1.524 | 1 | 0.198 | 0.658 | ns |
|  |  | NF Stage | 803.923 | 4 | 26.122 | <0.001 | *** |
|  |  | Sex:NF Stage | 10.795 | 4 | 0.351 | 0.843 | ns |
|  |  | Residuals | 523.186 | 68 |  |  |  |
| E | **SHI** | (Intercept) | 7323.891 | 1 | 1402.907 | <0.001 | *** |
|  |  | Block | 24.748 | 1 | 4.741 | 0.033 | * |
|  |  | Sex | 4.761 | 1 | 0.912 | 0.343 | ns |
|  |  | NF Stage | 878.863 | 4 | 42.087 | <0.001 | *** |
|  |  | Sex:NF Stage | 27.961 | 4 | 1.339 | 0.264 | ns |
|  |  | Residuals | 354.995 | 68 |  |  |  |
| F | **HSI** | (Intercept) | 76.188 | 1 | 2213.093 | <0.001 | *** |
|  |  | Block | 0.030 | 1 | 0.871 | 0.353 | ns |
|  |  | Sex | 0.002 | 1 | 0.052 | 0.820 | ns |
|  |  | NF Stage | 7.081 | 5 | 41.139 | <0.001 | *** |
|  |  | Sex:NF Stage | 0.182 | 5 | 1.055 | 0.392 | ns |
|  |  | Residuals | 2.720 | 79 |  |  |  |

## **Table S.4.** Results of post-hoc Tukey comparisons of Mass, Scaled Mass Index SMI, Liver Mass, Scaled Hepatic Index SHI, and Hepatosomatic Index HSI for prometamorphic and metamorphic climax developmental stages, including variance of spatial block and genetic sex. Significance definitions: ‘ *** ’ $\leq$ 0.001; ‘ ** ’ $\leq$ 0.01; ‘ * ’ $\leq$ 0.05; ‘ . ’ $\leq$0.1; ns = not significant.

| **Endpoint** | **Comparison** | **Estimate** | **SE** | **t value** | ***p*** | **Sig** |
| --- | --- | --- | --- | --- | --- | --- |
| **Mass** | 57 – 56 | 0.270 | 0.076 | 3.531 | 0.007 | ** |
|  | 58 – 56 | 0.427 | 0.076 | 5.582 | <0.001 | *** |
|  | 62 – 56 | 0.300 | 0.076 | 3.929 | 0.002 | ** |
|  | 66 – 56 | 0.074 | 0.069 | 1.072 | 0.819 | ns |
|  | 58 – 57 | 0.157 | 0.074 | 2.121 | 0.222 | ns |
|  | 62 – 57 | 0.030 | 0.074 | 0.412 | 0.994 | ns |
|  | 66 – 57 | -0.196 | 0.066 | -2.950 | 0.034 | * |
|  | 62 – 58 | -0.126 | 0.074 | -1.712 | 0.432 | ns |
|  | 66 – 58 | -0.353 | 0.066 | -5.304 | <0.001 | *** |
|  | 66 – 62 | -0.226 | 0.066 | -3.403 | 0.01 | ** |
| **SMI** | 57 – 56 | 0.284 | 0.046 | 6.112 | <0.001 | *** |
|  | 58 – 56 | 0.435 | 0.047 | 9.349 | <0.001 | *** |
|  | 62 – 56 | 0.285 | 0.047 | 6.128 | <0.001 | *** |
|  | 66 – 56 | 0.088 | 0.042 | 2.079 | 0.24 | ns |
|  | 58 – 57 | 0.151 | 0.045 | 3.349 | 0.011 | * |
|  | 62 – 57 | 0.001 | 0.045 | 0.018 | 1 | ns |
|  | 66 – 57 | -0.197 | 0.040 | -4.872 | <0.001 | *** |
|  | 62 – 58 | -0.150 | 0.045 | -3.336 | 0.012 | * |
|  | 66 – 58 | -0.347 | 0.040 | -8.588 | <0.001 | *** |
|  | 66 – 62 | -0.197 | 0.040 | -4.883 | <0.001 | *** |
| **Liver Mass** | 57 - 56 | 4.125 | 1.436 | 2.872 | 0.042 | * |
|  | 58 - 56 | 6.482 | 1.398 | 4.637 | <0.001 | *** |
|  | 62 - 56 | 12.767 | 1.436 | 8.889 | <0.001 | *** |
|  | 66 - 56 | 1.913 | 1.301 | 1.470 | 0.583 | ns |
|  | 58 - 57 | 2.357 | 1.349 | 1.748 | 0.411 | ns |
|  | 62 - 57 | 8.642 | 1.389 | 6.221 | <0.001 | *** |
|  | 66 - 57 | -2.212 | 1.246 | -1.775 | 0.395 | ns |
|  | 62 - 58 | 6.285 | 1.348 | 4.662 | <0.001 | *** |
|  | 66 - 58 | -4.569 | 1.203 | -3.797 | 0.003 | ** |
|  | 66 - 62 | -10.854 | 1.249 | -8.693 | <0.001 | *** |
| **SHI** | 57 - 56 | 4.301 | 1.183 | 3.636 | 0.005 | ** |
|  | 58 - 56 | 7.001 | 1.151 | 6.080 | <0.001 | *** |
|  | 62 - 56 | 13.181 | 1.183 | 11.140 | <0.001 | *** |
|  | 66 - 56 | 1.821 | 1.071 | 1.700 | 0.439 | ns |
|  | 58 - 57 | 2.700 | 1.111 | 2.431 | 0.119 | ns |
|  | 62 - 57 | 8.880 | 1.144 | 7.759 | <0.001 | *** |
|  | 66 - 57 | -2.480 | 1.027 | -2.415 | 0.123 | ns |
|  | 62 - 58 | 6.180 | 1.111 | 5.564 | <0.001 | *** |
|  | 66 - 58 | -5.180 | 0.991 | -5.225 | <0.001 | *** |
|  | 66 - 62 | -11.359 | 1.029 | -11.044 | <0.001 | *** |
| **HSI** | 56 - 51 | 0.279 | 0.098 | 2.849 | 0.06 | . |
|  | 57 - 51 | 0.459 | 0.091 | 5.051 | <0.001 | *** |
|  | 58 - 51 | 0.559 | 0.088 | 6.354 | <0.001 | *** |
|  | 62 - 51 | 1.250 | 0.090 | 13.823 | <0.001 | *** |
|  | 66 - 51 | 0.460 | 0.081 | 5.667 | <0.001 | *** |
|  | 57 - 56 | 0.180 | 0.100 | 1.794 | 0.473 | ns |
|  | 58 - 56 | 0.279 | 0.098 | 2.857 | 0.058 | . |
|  | 62 - 56 | 0.970 | 0.100 | 9.685 | <0.001 | *** |
|  | 66 - 56 | 0.181 | 0.092 | 1.977 | 0.363 | ns |
|  | 58 - 57 | 0.099 | 0.090 | 1.102 | 0.878 | ns |
|  | 62 - 57 | 0.790 | 0.093 | 8.508 | <0.001 | *** |
|  | 66 - 57 | 0.001 | 0.083 | 0.015 | 1 | ns |
|  | 62 - 58 | 0.691 | 0.090 | 7.662 | <0.001 | *** |
|  | 66 - 58 | -0.098 | 0.080 | -1.220 | 0.824 | ns |
|  | 66 - 62 | -0.789 | 0.084 | -9.451 | <0.001 | *** |

## **Table S.5.** Welch’s ANOVA tables reporting comparison of mean Mass, Scaled Mass Index SMI, Snout-Vent Length SVL, Liver Mass, Scaled Hepatic Index SHI, Fat Body Mass, and Fat Somatic Index FSI across NF stages. Significance definitions: ‘ *** ’ $\leq$ 0.001; ‘ ** ’ $\leq$ 0.01; ‘ * ’ $\leq$ 0.05; ‘ . ’ $\leq$0.1; ns = not significant.

| **Response** | **n** | **Welch’s F** | **DF num** | **DF den** | ***p*** | **Sig** |
| --- | --- | --- | --- | --- | --- | --- |
| Mass | 113 | 767.19 | 6 | 39.305 | <0.001 | *** |
| SMI | 113 | 2072.19 | 6 | 39.078 | <0.001 | *** |
| SVL | 113 | 1458.75 | 6 | 44.29 | <0.001 | *** |
| Liver Mass  SHI | 95  94 | 296.66  369.47 | 5  5 | 35.195  35.548 | <0.001  <0.001 | ***  *** |
| Fat Body Mass | 79 | 52.75 | 4 | 33.806 | <0.001 | *** |
| FSI | 79 | 69.29 | 4 | 34.539 | <0.001 | *** |
| SFI | 77 | 174.2 | 4 | 31.015 | <0.001 | *** |

## **Table S.6.** Results of post-hoc Games-Howells comparisons of Mass, Scaled Mass Index SMI, Snout-Vent Length SVL, Liver Mass, Scaled Hepatic Index SHI, Fat Body Mass, Scaled Fat Index SFI, and Fat Scaled Index FSI across NF stages. It should be noted that Games-Howell post-hoc tests cannot account for variance associated with spatial block or genetic sex. Significance definitions: ‘ *** ’ $\leq$ 0.001; ‘ ** ’ $\leq$ 0.01; ‘ * ’ $\leq$ 0.05; ‘ . ’ $\leq$0.1; ns = not significant.

| **Endpoint** | **Group 1** | **Group 2** | **Estimate** | **95% CI Lower** | **95% CI Upper** | ***p*** | **Sig** |
| --- | --- | --- | --- | --- | --- | --- | --- |
| **Mass** | 48 | 51 | 0.097 | 0.084 | 0.110 | <0.001 | *** |
|  | 48 | 56 | 0.759 | 0.664 | 0.854 | <0.001 | *** |
|  | 48 | 57 | 1.016 | 0.854 | 1.178 | <0.001 | *** |
|  | 48 | 58 | 1.137 | 0.948 | 1.326 | <0.001 | *** |
|  | 48 | 62 | 1.036 | 0.891 | 1.181 | <0.001 | *** |
|  | 48 | 66 | 0.821 | 0.764 | 0.879 | <0.001 | *** |
|  | 51 | 56 | 0.662 | 0.567 | 0.758 | <0.001 | *** |
|  | 51 | 57 | 0.919 | 0.757 | 1.081 | <0.001 | *** |
|  | 51 | 58 | 1.040 | 0.851 | 1.230 | <0.001 | *** |
|  | 51 | 62 | 0.939 | 0.794 | 1.084 | <0.001 | *** |
|  | 51 | 66 | 0.725 | 0.666 | 0.783 | <0.001 | *** |
|  | 56 | 57 | 0.257 | 0.079 | 0.434 | 0.002 | ** |
|  | 56 | 58 | 0.378 | 0.177 | 0.580 | <0.001 | *** |
|  | 56 | 62 | 0.277 | 0.115 | 0.439 | <0.001 | *** |
|  | 56 | 66 | 0.063 | -0.043 | 0.168 | 0.501 | ns |
|  | 57 | 58 | 0.122 | -0.110 | 0.353 | 0.641 | ns |
|  | 57 | 62 | 0.020 | -0.181 | 0.222 | 1.000 | ns |
|  | 57 | 66 | -0.194 | -0.361 | -0.027 | 0.017 | * |
|  | 58 | 62 | -0.101 | -0.323 | 0.120 | 0.766 | ns |
|  | 58 | 66 | -0.316 | -0.509 | -0.122 | 0.001 | *** |
|  | 62 | 66 | -0.215 | -0.365 | -0.064 | 0.003 | ** |
| **SMI** | 48 | 51 | 0.097 | 0.090 | 0.103 | <0.001 | *** |
|  | 48 | 56 | 0.756 | 0.707 | 0.805 | <0.001 | *** |
|  | 48 | 57 | 1.013 | 0.917 | 1.108 | <0.001 | *** |
|  | 48 | 58 | 1.133 | 1.018 | 1.248 | <0.001 | *** |
|  | 48 | 62 | 1.032 | 0.954 | 1.110 | <0.001 | *** |
|  | 48 | 66 | 0.821 | 0.779 | 0.864 | <0.001 | *** |
|  | 51 | 56 | 0.660 | 0.611 | 0.709 | <0.001 | *** |
|  | 51 | 57 | 0.916 | 0.821 | 1.012 | <0.001 | *** |
|  | 51 | 58 | 1.036 | 0.921 | 1.151 | <0.001 | *** |
|  | 51 | 62 | 0.935 | 0.857 | 1.014 | <0.001 | *** |
|  | 51 | 66 | 0.725 | 0.682 | 0.767 | <0.001 | *** |
|  | 56 | 57 | 0.257 | 0.155 | 0.359 | <0.001 | *** |
|  | 56 | 58 | 0.376 | 0.256 | 0.497 | <0.001 | *** |
|  | 56 | 62 | 0.276 | 0.189 | 0.362 | <0.001 | *** |
|  | 56 | 66 | 0.065 | 0.004 | 0.126 | 0.030 | * |
|  | 57 | 58 | 0.120 | -0.019 | 0.259 | 0.126 | ns |
|  | 57 | 62 | 0.019 | -0.095 | 0.134 | 0.998 | ns |
|  | 57 | 66 | -0.192 | -0.292 | -0.091 | <0.001 | *** |
|  | 58 | 62 | -0.101 | -0.231 | 0.029 | 0.209 | ns |
|  | 58 | 66 | -0.312 | -0.431 | -0.192 | <0.001 | *** |
|  | 62 | 66 | -0.211 | -0.295 | -0.126 | <0.001 | *** |
| **SVL** | 48 | 51 | 5.959 | 5.352 | 6.567 | <0.001 | *** |
|  | 48 | 56 | 17.817 | 16.770 | 18.863 | <0.001 | *** |
|  | 48 | 57 | 19.460 | 17.806 | 21.114 | <0.001 | *** |
|  | 48 | 58 | 20.062 | 18.312 | 21.812 | <0.001 | *** |
|  | 48 | 62 | 13.374 | 12.456 | 14.292 | <0.001 | *** |
|  | 48 | 66 | 14.933 | 14.353 | 15.514 | <0.001 | *** |
|  | 51 | 56 | 11.858 | 10.797 | 12.918 | <0.001 | *** |
|  | 51 | 57 | 13.501 | 11.840 | 15.162 | <0.001 | *** |
|  | 51 | 58 | 14.103 | 12.346 | 15.860 | <0.001 | *** |
|  | 51 | 62 | 7.415 | 6.480 | 8.350 | <0.001 | *** |
|  | 51 | 66 | 8.974 | 8.358 | 9.591 | <0.001 | *** |
|  | 56 | 57 | 1.643 | -0.157 | 3.443 | 0.089 | . |
|  | 56 | 58 | 2.245 | 0.360 | 4.131 | 0.013 | * |
|  | 56 | 62 | -4.443 | -5.666 | -3.220 | <0.001 | *** |
|  | 56 | 66 | -2.883 | -3.934 | -1.832 | <0.001 | *** |
|  | 57 | 58 | 0.602 | -1.601 | 2.805 | 0.975 | ns |
|  | 57 | 62 | -6.086 | -7.834 | -4.338 | <0.001 | *** |
|  | 57 | 66 | -4.527 | -6.183 | -2.870 | <0.001 | *** |
|  | 58 | 62 | -6.688 | -8.525 | -4.851 | <0.001 | *** |
|  | 58 | 66 | -5.129 | -6.881 | -3.376 | <0.001 | *** |
|  | 62 | 66 | 1.559 | 0.636 | 2.482 | <0.001 | *** |
| **Liver Mass** | 51 | 56 | 6.900 | 5.844 | 7.956 | <0.001 | *** |
|  | 51 | 57 | 10.713 | 8.276 | 13.150 | <0.001 | *** |
|  | 51 | 58 | 13.753 | 11.392 | 16.115 | <0.001 | *** |
|  | 51 | 62 | 20.171 | 16.595 | 23.748 | <0.001 | *** |
|  | 51 | 66 | 9.395 | 7.728 | 11.063 | <0.001 | *** |
|  | 56 | 57 | 3.813 | 1.263 | 6.364 | 0.002 | ** |
|  | 56 | 58 | 6.853 | 4.373 | 9.333 | <0.001 | *** |
|  | 56 | 62 | 13.271 | 9.628 | 16.915 | <0.001 | *** |
|  | 56 | 66 | 2.495 | 0.625 | 4.366 | 0.004 | ** |
|  | 57 | 58 | 3.040 | -0.116 | 6.196 | 0.064 | . |
|  | 57 | 62 | 9.458 | 5.404 | 13.512 | <0.001 | *** |
|  | 57 | 66 | -1.318 | -4.108 | 1.472 | 0.699 | ns |
|  | 58 | 62 | 6.418 | 2.397 | 10.439 | 0.001 | *** |
|  | 58 | 66 | -4.358 | -7.086 | -1.630 | 0.001 | *** |
|  | 62 | 66 | -10.776 | -14.563 | -6.990 | <0.001 | *** |
| **SHI** | 51 | 56 | 6.994 | 5.749 | 8.239 | <0.001 | *** |
|  | 51 | 57 | 10.770 | 8.694 | 12.847 | <0.001 | *** |
|  | 51 | 58 | 13.825 | 11.842 | 15.808 | <0.001 | *** |
|  | 51 | 62 | 21.132 | 18.319 | 23.944 | <0.001 | *** |
|  | 51 | 66 | 9.413 | 7.918 | 10.907 | <0.001 | *** |
|  | 56 | 57 | 3.777 | 1.497 | 6.056 | <0.001 | *** |
|  | 56 | 58 | 6.831 | 4.633 | 9.030 | <0.001 | *** |
|  | 56 | 62 | 14.138 | 11.194 | 17.082 | <0.001 | *** |
|  | 56 | 66 | 2.419 | 0.600 | 4.238 | 0.004 | ** |
|  | 57 | 58 | 3.055 | 0.389 | 5.720 | 0.018 | * |
|  | 57 | 62 | 10.361 | 7.102 | 13.621 | <0.001 | *** |
|  | 57 | 66 | -1.358 | -3.764 | 1.049 | 0.528 | ns |
|  | 58 | 62 | 7.307 | 4.093 | 10.521 | <0.001 | *** |
|  | 58 | 66 | -4.412 | -6.744 | -2.080 | <0.001 | *** |
|  | 62 | 66 | -11.719 | -14.750 | -8.688 | <0.001 | *** |
| **Fat Body Mass** | 56 | 57 | 1.120 | 0.230 | 2.009 | 0.009 | ** |
|  | 56 | 58 | 2.973 | 1.536 | 4.410 | <0.001 | *** |
|  | 56 | 62 | 8.307 | 6.178 | 10.436 | <0.001 | *** |
|  | 56 | 66 | 3.360 | 2.071 | 4.648 | <0.001 | *** |
|  | 57 | 58 | 1.853 | 0.303 | 3.404 | 0.014 | * |
|  | 57 | 62 | 7.188 | 4.994 | 9.381 | <0.001 | *** |
|  | 57 | 66 | 2.240 | 0.812 | 3.668 | 0.001 | *** |
|  | 58 | 62 | 5.334 | 2.947 | 7.722 | <0.001 | *** |
|  | 58 | 66 | 0.387 | -1.388 | 2.161 | 0.969 | ns |
|  | 62 | 66 | -4.948 | -7.282 | -2.614 | <0.001 | *** |
| **SFI** | 56 | 57 | 1.168 | -0.016 | 2.353 | 0.054 | . |
|  | 56 | 58 | 3.519 | 1.361 | 5.676 | 0.001 | *** |
|  | 56 | 62 | 8.281 | 7.317 | 9.244 | <0.001 | *** |
|  | 56 | 66 | 3.856 | 1.850 | 5.863 | <0.001 | *** |
|  | 57 | 58 | 2.351 | 0.029 | 4.672 | 0.046 | * |
|  | 57 | 62 | 7.112 | 5.731 | 8.493 | <0.001 | *** |
|  | 57 | 66 | 2.688 | 0.484 | 4.892 | 0.011 | * |
|  | 58 | 62 | 4.762 | 2.513 | 7.010 | <0.001 | *** |
|  | 58 | 66 | 0.337 | -2.424 | 3.098 | 0.997 | ns |
|  | 62 | 66 | -4.425 | -6.543 | -2.306 | <0.001 | *** |
| **FSI** | 56 | 57 | 0.095 | -0.002 | 0.192 | 0.057 | . |
|  | 56 | 58 | 0.242 | 0.113 | 0.371 | <0.001 | *** |
|  | 56 | 62 | 0.765 | 0.612 | 0.918 | <0.001 | *** |
|  | 56 | 66 | 0.394 | 0.250 | 0.538 | <0.001 | *** |
|  | 57 | 58 | 0.147 | 0.003 | 0.291 | 0.044 | * |
|  | 57 | 62 | 0.671 | 0.506 | 0.835 | <0.001 | *** |
|  | 57 | 66 | 0.299 | 0.141 | 0.457 | <0.001 | *** |
|  | 58 | 62 | 0.523 | 0.342 | 0.705 | <0.001 | *** |
|  | 58 | 66 | 0.152 | -0.024 | 0.328 | 0.119 | ns |
|  | 62 | 66 | -0.372 | -0.564 | -0.179 | <0.001 | *** |

## **Table S.7**. ANOVA tables (Type III) reporting comparison of mean ln(Fold Change) of *xPPARα, xPPARβ,* and *xPPARγ* across development. Significance definitions: ‘ *** ’ $\leq$ 0.001; ‘ ** ’ $\leq$ 0.01; ‘ * ’ $\leq$ 0.05; ‘ . ’ $\leq$0.1; ns = not significant.

| **Endpoint** | **Source of Variance** | **SS** | **df** | ***F*** | ***p*** | **Sig** |
| --- | --- | --- | --- | --- | --- | --- |
| ***xPPARα*** | (Intercept) | 38.099 | 1 | 142.187 | <0.001 | *** |
|  | Block | 0.880 | 1 | 3.285 | 0.077 | . |
|  | NF Stage | 9.002 | 6 | 5.599 | <0.001 | *** |
|  | Residuals | 10.718 | 40 |  |  |  |
| ***xPPARβ*** | (Intercept) | 9.435 | 1 | 28.118 | <0.001 | *** |
|  | Block | 1.102 | 1 | 3.285 | 0.078 | . |
|  | NF Stage | 4.820 | 6 | 2.394 | 0.046 | * |
|  | Residuals | 13.086 | 39 |  |  |  |
| ***xPPARγ*** | (Intercept) | 49.932 | 1 | 107.572 | <0.001 | *** |
|  | Block | 1.649 | 1 | 3.553 | 0.067 | **. |
|  | NF Stage | 19.162 | 6 | 6.880 | <0.001 | *** |
|  | Residuals | 18.103 | 39 |  |  |  |

## **Table S.8.** Results of post-hoc Dunnett comparisons of ln(Fold Change) for *xPPARα, xPPARβ,* and *xPPARγ* across development. Significance definitions: ‘ *** ’ $\leq$ 0.001; ‘ ** ’ $\leq$ 0.01; ‘ * ’ $\leq$ 0.05; ‘ . ’ $\leq$0.1; ns = not significant.

| **Endpoint** | **Comparison** | **Estimates** | **SE** | **t value** | ***p*** | **Sig** |
| --- | --- | --- | --- | --- | --- | --- |
| **xPPAR*α*** | 51-48 | 1.450 | 0.295 | 4.912 | <0.001 | *** |
|  | 56-48 | 1.167 | 0.280 | 4.163 | 0.001 | *** |
|  | 57-48 | 0.947 | 0.260 | 3.648 | 0.004 | ** |
|  | 58-48 | 1.179 | 0.280 | 4.206 | 0.001 | *** |
|  | 62-48 | 0.831 | 0.260 | 3.203 | 0.014 | * |
|  | 66-48 | 1.017 | 0.268 | 3.794 | 0.003 | ** |
| **xPPAR*β*** | 51-48 | 0.818 | 0.339 | 2.412 | 0.093 | . |
|  | 56-48 | 0.502 | 0.323 | 1.557 | 0.446 | ns |
|  | 57-48 | 0.257 | 0.300 | 0.855 | 0.901 | ns |
|  | 58-48 | 0.719 | 0.323 | 2.228 | 0.137 | ns |
|  | 62-48 | 0.785 | 0.300 | 2.617 | 0.059 | . |
|  | 66-48 | 0.934 | 0.310 | 3.016 | 0.023 | * |
| **xPPAR*γ*** | 51-48 | 1.359 | 0.399 | 3.407 | 0.008 | *** |
|  | 56-48 | 1.689 | 0.379 | 4.452 | <0.001 | *** |
|  | 57-48 | 1.413 | 0.353 | 4.004 | 0.002 | ** |
|  | 58-48 | 1.879 | 0.379 | 4.954 | <0.001 | *** |
|  | 62-48 | 0.428 | 0.353 | 1.214 | 0.684 | ns |
|  | 66-48 | 1.290 | 0.364 | 3.543 | 0.006 | ** |

## **Table S.9.** ANOVA tables (Type III) reporting comparison of mean mass, Scaled Mass Index SMI, Snout-Vent Length SVL, liver mass, and Hepatosomatic index HSI across *xPPAR* agonist treatments after accounting for variation in temporal block and genetic sex. Significance definitions: ‘ *** ’ $\leq$ 0.001; ‘ ** ’ $\leq$ 0.01; ‘ * ’ $\leq$ 0.05; ‘ . ’ $\leq$0.1; ns = not significant.

| **Panel** | **Endpoint** | **Source of Variance** | **SS** | **df** | **F** | ***p*** | **Sig** |
| --- | --- | --- | --- | --- | --- | --- | --- |
| A | **Mass** | (Intercept) | 16.643 | 1 | 2032.402 | <0.001 | *** |
|  |  | Block | 0.004 | 2 | 0.273 | 0.763 | ns |
|  |  | Sex | 0.008 | 1 | 0.934 | 0.340 | ns |
|  |  | Agonist | 0.005 | 3 | 0.192 | 0.901 | ns |
|  |  | Sex:Agonist | 0.031 | 3 | 1.244 | 0.307 | ns |
|  |  | Residuals | 0.311 | 38 |  |  |  |
| B | **SMI** | (Intercept) | 15.387 | 1 | 7784.047 | <0.001 | *** |
|  |  | Block | 0.001 | 2 | 0.295 | 0.746 | ns |
|  |  | Sex | 0.002 | 1 | 0.944 | 0.338 | ns |
|  |  | Agonist | 0.023 | 3 | 3.831 | 0.017 | * |
|  |  | Sex:Agonist | 0.039 | 3 | 6.567 | 0.001 | *** |
|  |  | Residuals | 0.075 | 38 |  |  |  |
| C | **SVL** | (Intercept) | 10899.373 | 1 | 9028.252 | <0.001 | *** |
|  |  | Block | 0.170 | 2 | 0.070 | 0.932 | ns |
|  |  | Sex | 0.423 | 1 | 0.351 | 0.558 | ns |
|  |  | Agonist | 7.552 | 3 | 2.085 | 0.119 | ns |
|  |  | Sex:Agonist | 10.067 | 3 | 2.779 | 0.055 | . |
|  |  | Residuals | 43.461 | 36 |  |  |  |
| D | **Liver Mass** | (Intercept) | 0.001 | 1 | 632.055 | <0.001 | *** |
|  |  | Block | 0.000 | 2 | 4.077 | 0.025 | * |
|  |  | Sex | 0.000 | 1 | 0.426 | 0.518 | ns |
|  |  | Agonist | 0.000 | 3 | 0.114 | 0.951 | ns |
|  |  | Sex:Agonist | 0.000 | 3 | 0.330 | 0.803 | ns |
|  |  | Residuals | 0.000 | 38 |  |  |  |
| E | **HSI** | (Intercept) | 12.060 | 1 | 441.999 | <0.001 | *** |
|  |  | Block | 0.106 | 2 | 1.943 | 0.157 | ns |
|  |  | Sex | 0.000 | 1 | 0.001 | 0.982 | ns |
|  |  | Agonist | 0.003 | 3 | 0.042 | 0.988 | ns |
|  |  | Sex:Agonist | 0.021 | 3 | 0.262 | 0.852 | ns |
|  |  | Residuals | 1.037 | 38 |  |  |  |

## **Table S.10.** ANOVA tables (Type III) reporting comparison of mean Scaled Mass Index SMI by sex across *xPPAR* agonist treatments after accounting for variation in temporal block. Significance definitions: ‘ *** ’ $\leq$ 0.001; ‘ ** ’ $\leq$ 0.01; ‘ * ’ $\leq$ 0.05; ‘ . ’ $\leq$0.1; ns = not significant.

| **Genetic Sex** | **Source of Variance** | **SS** | **df** | ***F*** | ***p*** | **Sig** |
| --- | --- | --- | --- | --- | --- | --- |
| **Male** | (Intercept) | 17.832 | 1 | 10575.282 | <0.001 | *** |
|  | Block | 0.002 | 2 | 0.477 | 0.628 | ns |
|  | Agonist | 0.018 | 3 | 3.511 | 0.034 | * |
|  | Residuals | 0.034 | 20 |  |  |  |
| **Female** | (Intercept) | 15.183 | 1 | 6447.054 | <0.001 | *** |
|  | Block | 0.003 | 2 | 0.694 | 0.514 | ns |
|  | Agonist | 0.023 | 3 | 3.319 | 0.047 | * |
|  | Residuals | 0.038 | 16 |  |  |  |

## **Table S.11.** Results of post-hoc Dunnett comparisons of Scaled Mass Index SMI for genetic male and female tadpoles exposed to either a DMSO vehicle control or an agonist treatment (Bezafibrate (BZ), Ciprofibrate (CP), Pirinixic Acid (PA)). Significance definitions: ‘ *** ’ $\leq$ 0.001; ‘ ** ’ $\leq$ 0.01; ‘ * ’ $\leq$ 0.05; ‘ . ’ $\leq$0.1; ns = not significant.

| **Endpoint** | **Comparison** | **Estimates** | **SE** | **t value** | ***p*** | **Sig** |
| --- | --- | --- | --- | --- | --- | --- |
| **Male** | BZ - DMSO | 0.001 | 0.025 | 0.030 | 1.000 | ns |
|  | CP - DMSO | -0.017 | 0.023 | -0.746 | 0.794 | ns |
|  | PA - DMSO | 0.054 | 0.024 | 2.277 | 0.084 | . |
| **Female** | BZ - DMSO | -0.035 | 0.030 | -1.178 | 0.528 | ns |
|  | CP - DMSO | 0.010 | 0.010 | 0.306 | 0.982 | ns |
|  | PA - DMSO | -0.074 | 0.028 | -2.656 | 0.045 | * |

##

## **Table S.12.** ANOVA tables (Type III) reporting comparison of mean relative mRNA of *acox1*, *apoa5*, *fabp1*, and *pck1* for *X. laevis* livers across *xPPAR* agonist treatments after controlling for variation in temporal block and genetic sex. Significance definitions: ‘ *** ’ $\leq$ 0.001; ‘ ** ’ $\leq$ 0.01; ‘ * ’ $\leq$ 0.05; ‘ . ’ $\leq$0.1; ns = not significant.

| **Endpoint** | **Source of Variance** | **SS** | **df** | ***F*** | ***p*** | **Sig** |
| --- | --- | --- | --- | --- | --- | --- |
| ***acox1*** | (Intercept) | 0.442 | 1 | 0.710 | 0.405 | ns |
|  | Block | 0.101 | 2 | 0.081 | 0.923 | ns |
|  | Sex | 0.495 | 1 | 0.794 | 0.379 | ns |
|  | Agonist | 0.634 | 3 | 0.340 | 0.797 | ns |
|  | Sex:Agonist | 0.323 | 3 | 0.173 | 0.914 | ns |
|  | Residuals | 23.047 | 37 |  |  |  |
| ***apoa5*** | (Intercept) | 3.294 | 1 | 2.632 | 0.114 | ns |
|  | Block | 1.397 | 2 | 0.558 | 0.577 | ns |
|  | Sex | 3.206 | 1 | 2.562 | 0.118 | ns |
|  | Agonist | 2.955 | 3 | 0.787 | 0.509 | ns |
|  | Sex:Agonist | 1.406 | 3 | 0.374 | 0.772 | ns |
|  | Residuals | 43.803 | 35 |  |  |  |
| ***fabp1*** | (Intercept) | 0.343 | 1 | 0.456 | 0.504 | ns |
|  | Block | 0.508 | 2 | 0.337 | 0.716 | ns |
|  | Sex | 0.011 | 1 | 0.014 | 0.905 | ns |
|  | Agonist | 3.931 | 3 | 1.740 | 0.176 | ns |
|  | Sex:Agonist | 5.231 | 3 | 2.315 | 0.092 | . |
|  | Residuals | 27.864 | 37 |  |  |  |
| ***pck1*** | (Intercept) | 0.016 | 1 | 0.015 | 0.902 | ns |
|  | Block | 8.649 | 2 | 4.196 | 0.023 | * |
|  | Sex | 1.864 | 1 | 1.809 | 0.187 | ns |
|  | Agonist | 2.229 | 3 | 0.721 | 0.546 | ns |
|  | Sex:Agonist | 0.904 | 3 | 0.293 | 0.830 | ns |
|  | Residuals | 37.102 | 36 |  |  |  |

**Table S.13.** CV analysis by developmental stage for liver samples using pilot data and experimental data. The coefficient of variation (CV) is a ratio of the Standard Deviation to the mean of the linearized Ct values expressed as a percentage. The mean Ct for technical duplicates are the values linearized. To interpret, a lower CV value would be desirable as it would indicate a lower variation across samples and a higher stability. Pilot qPCR data generated using samples treated with perfluorinated alkyl substances for a different study. Please reference supplementary GitHub link for spreadsheets with raw data and calculations.

| Pilot qPCR Data for *sub1* for liver samples | | | | | |
| --- | --- | --- | --- | --- | --- |
| **All Stages (NF 58, 62, 66)** | | **By Stage** | **NF 58** | **NF 62** | **NF 66** |
| Mean 2^-(Ct) | 1.23E-08 | Mean 2^-(Ct) | 1.04E-08 | 1.33E-08 | 1.32E-08 |
| SD 2^-(Ct) | 5.31E-09 | SD 2^-(Ct) | 8.22E-09 | 3.09E-09 | 3.54E-09 |
| **CV (SD/Mean) x100%** | 43.20 | **CV (SD/Mean) x100%** | 78.94 | 23.16 | 26.86 |

| qPCR data for *sub1* for liver samples of characterization study | | | | | | | | | |
| --- | --- | --- | --- | --- | --- | --- | --- | --- | --- |
| **All Stages (NF 51, 56, 57, 58, 62, 66)** |  | **By Stage** | **NF 51** | **NF 56** | **NF 57** | **NF 58** | **NF 62** | **NF 66** |  |
| Mean 2^-(Ct) | 3.20E-08 | Mean 2^-(Ct) | 3.74E-08 | 3.80E-08 | 4.30E-08 | 3.24E-08 | 1.55E-08 | 2.89E-08 |  |
| SD 2^-(Ct) | 1.89E-08 | SD 2^-(Ct) | 2.29E-08 | 1.59E-08 | 1.91E-08 | 2.08E-08 | 8.06E-09 | 1.75E-08 |  |
| **CV (SD/Mean) x100%** | 59.09 | **CV (SD/Mean) x100%** | 61.11 | 41.91 | 44.46 | 64.11 | 52.06 | 60.46 |  |

**References**

Mughal, B. B., M. Leemans, P. Spirhanzlova, B. Demeneix, and J. B. Fini. 2018. “Reference Gene Identification and Validation for Quantitative Real‐Time PCR Studies in Developing Xenopus laevis.” *Scientific Reports* 8, no. 1: 496. https://doi.org/10.1038/s41598-017-18684-1.

OECD. 2009. *Test No. 231: Amphibian Metamorphosis Assay, OECD Guidelines for the Testing of Chemicals, Section 2*. Paris: OECD Publishing. https://doi.org/10.1787/9789264076242-en.

Tamaoki, K., A. Ishihara, and K. Yamauchi. 2018. “Expression Pattern and Histone Acetylation of Energy Metabolic Genes in Xenopus laevis Liver in Response to Diet Statuses.” *Journal of Experimental Zoology Part A: Ecological and Integrative Physiology* 331, no. 2: 120–127. https://doi.org/10.1002/jez.2246.
