# Supplementary material for: Developmental Patterns of Hepatic Peroxisome Proliferator‐Activated Receptor (PPAR) Expression in Xenopus laevis and Response to Pharmaceutical Agonists During Metamorphic Climax
Source: J Exp Zool A Ecol Integr Physiol. 2025 Sep 3;343(10):1191–204. doi: 10.1002/jez.70031 (PMC12604685; doi:10.1002/jez.70031)

Supplementary Information for Primers, PCR, & qPCR assays

Table of Contents

[Text 1. Genotypic Sexing PCR assays 2](#_Toc173502912)

[Text 2. qPCR assays 2](#_Toc173502913)

[Text 3. *acox1* primer documentation 2](#_Toc173502914)

[Text 4. *apoa5* primer documentation 5](#_Toc173502915)

[Text 5. *fabp1* primer documentation 8](#_Toc173502916)

[Text 6. *pck1* primer documentation 11](#_Toc173502917)

[Text 7. *sub1* primer documentation 13](#_Toc173502918)

[Text 8. *xPPARa* primer documentation 16](#_Toc173502919)

[Text 9. *xPPARb* primer documentation 20](#_Toc173502920)

[Text 10. *xPPARg* primer documentation 22](#_Toc173502921)

# **Text 1.** Genotypic Sexing PCR assays

We used the following thermal profile for PCR assay for genotypic sexing of *X. laevis*: (a) 1 cycle at 95^o^C for 1 min, (b) 35 cycles of 95^o^C for 30 sec, 60^o^C for 30 sec, 72^o^C for 1 sec, and (c) 1 cycle of 72^o^C for 1 min. PCR product was visualized using gel electrophoresis (2% agarose gel, SYBR^TM^ Safe) to determine the presence of the DM-W and DMRT-1 gene. Product for DM-W should visualize at ~250 bp and DMRT-1 at ~200 bp.

# **Text 2.** qPCR assays

We used the following thermal profile for qPCR assays for amplification of xPPAR target gene of interest: (a) 1 cycle at 95^o^C for 1 min, (b) 40 cycles of 95^o^C for 15 sec, primer-specific annealing temperature for 30 sec, 72^o^C for 30 sec, and (c) 1 cycle of 72^o^C for 30 sec. Each assay concluded with a continuous melt curve to check for non-target amplification in the product. Each cDNA sample representing a biological individual was assigned to a plate map. Each plate map underwent a single freeze-thaw cycle and assay for each target gene conducted within a 12-hour period.

# **Text 3.** *acox1* primer documentation

**ACOX1**, Acyl-CoA oxidase 1, palmitoyl L homeolog (*acox1.L*): target species—African Clawed Frog (*Xenopus laevis*)

**F/Left Primer (5^’^-3^’^):** ACATGGGATCAGCAAGAACAC

21 bp Tm 55.4°C 47.6%GC

**R/Right Primer(5^’^-3^’^**): CCACTGCATTAGGACGGATTA

**In sequence:** TAATCCGTCCTAATGCAGTGG

21 bp Tm 54.4°C 47.6%GC

**Source:** Tamaoki et al., 2018; https://doi.org/10.1002/jez.2246

**Spans exon-exon:** Yes

**Amplicon Product:** 127 bp

Start & End of reading frame

Predicted F primer sequence

Predicted R Primer sequence

>NM_001096680.1 Xenopus laevis acyl-CoA oxidase 1, palmitoyl L homeolog (acox1.L), mRNA

GAGAGACAAGAGAATGAACCCGGACTTGAGCAGGGAAAGGGCCGCCGCCTCCTTCAACCCCGAGACCATC

ACCTACATCCTGGATGGCAGCCCCGAGAGGACCCACAGGAGGAGAGAAATTGAGGGTCTGGTTCTCAATG

ACCCCGGCTTCCAGCATGAAGACCCCAATTTCCTGTCTCGCAGTGAGCGTTATGAATTGGCCATCAAGAA

GAGTGCCCTGATGGTTCAGAAGATGAGGGAACATGGCATCTCTGACCCAGAGGAAATCTACTGGTTCAAA

AGTTTTGTTCACCGGGGCCGCCCAGAGCCCCTAGATCTTCATCTGGGCATGTTCTTGCCCACGCTATTGA

ACCAGGCTACACCCGAGCAGCAGGAGCGTCTCTTCATGCCTGCCTGGAACCTGGAGATCATTGGGACCTA

TGCTCAAACTGAGATGGGACATGGAACCCACCTCCGAGGGCTGGAGACCACAGCTACATATGATCCATCC

ACCCAGGAGTTCATCCTCAACAGCCCAACTGTCTCTTCTATCAAATGGTGGCCTGGAGGATTGGGAAAAA

CCTCAAACCACGCTGTAGTGCTGGCACAACTTTACACACAAGGAGAATGTAAAGGGCTTCATGCTTTTAT

AGTGCCCATCCGCCACATGGGCACCCATGAGCCTCTTCCAGGTGTGATGGTTGGAGACATAGGGCCTAAG

TTTGGGTTTGATGAAACAGATAACGGGTTCCTTAAGTTTGACAAGGTTCGGATTCCTCGAGAGTATATGA

TGATGAAGTACGCCAAGGTTGAGCCAGATGGAACTTACGTGAAGCCACTCAGTGACAAACTGACCTATGG

CACAATGGTGTTCATTCGTTCAATGATTGTAGGAGACTCTGCACGCAGCCTTTCCCGTGCCTGTACCATC

GCTGTCCGGTATAGTACCGTGAGGCACCAGTCAGAGATCAGAGCTGGCGAGCCAGAGCCCCAAATCCTAG

ACTTTCAAACTCAGCAATACAAGTTATTCCCGCTGCTGGCTACAGCATACGCCTTCCAGTTTGTGGGATC

CTACATGAACCACACTTATCACAGAATCAGCGCTGAAATCCAAGATGGCAATCTGAATGAACTGCCCGAG

CTGCATGCCCTTTCTGCGGGCTTGAAAGCCTTCACAACATGGGTAGCCAACGCAGGTATTGAAGAGTGCA

GGATGGCATGTGGCGGACACGGATACTCCCGTTGCAGTGGGATACCTGATATCTATGTGACTTTTACCCC

TGCATGCACATATGAGGGTGAAAACACAGTGATGATGCTACAAACTGCAAGGTTTTTGGTGAAAAGCTAC

GAAGCTGTTCTTTCTGGGAAACAACTTGATGGAATGGTGTCATACCTCAATGATGTTTCCCAGCACCGTG

TACAGCCCCACCCTGTTGCCGGCAGATTCCTTGTCAAGGACATCAATGACATTCAAAATCTGGTGGAGGC

CTACAAACAACGAGCTGCTTGGTTAGTTGTGGCTGCTGCCAATAGTGTAAACGCCGACTCAAAACGTGGA

AAGCGCAAGGAAGACGCATGGAACAAGAACTCTGTAGATCTGGTCCGAGCTTCAGAGGCCCACTGTCATT

ATGTGGTTGTGAGACTCTTTGCGGAAAAGTTGTCTGAGGTTCTCGATGTGGCTGCCCATCGTATTCTGAG

CTCTCTCTGCCTTTTCTACGCGCTACATGGGATCAGCAAGAACACTGGGGACTTCCTTCAGGCTGGACTT

CTGACCGCTCTTCAGGTAAATCAAGTGCAGCAAAGAGTAAAGGATCTGCTGGCAGTAATCCGTCCTAATG

CAGTGGCTTTGGTGGATGCCTTTGATTACTCAGACACACAACTGGGATCTGTTCTGGGCAGATATGACGG

TAACGTCTATGAGAATATGTTTGAGTGGGCAAAGAAATCACCGCTGAACAAAACACAGGTGCACGAATCC

TTCCACAAGTACTTGAAACCACTACAGTCCAAACTATGATCCACCCATCAATTCTGCTGAACCCGAATCT

GGAAACACAAAAAGCAGTTCTCTTTTTTTTTGATAACCTACCCTCGAAGTAATAAAACTGTTTTCCTAAT

TTCTGTCAAATTATGTTTATTAACAAGGGGCCAAAGCAATGTGTAGGCAATGTAATCCTACATTAACAGA

GATCTTATCAACCTTTTGGCACAGCCAGTTGTTGCTCTAGGTCGGAGATGACGACATAGCTAGTACAGTC

ATGCCGACCTGTAGAAACTAAAGGCATACACACCCTTTATAAGATCAGCATTGTTTGTATAGTGGTTAAT

AAACAAAAGTAGGAGCAATGACCCATCTAACCAGCAGAGGTGCCTCCCTAATAATGTTAAAAGGAAGTGA

AAATCCAACTTACAATAAACCTTTAAGAATTATGTAGTCAATGATATAATAAGATTAGTCTGGGTCTAGA

ATTTGAGCTGTTTCCCTGTTTCTTTGCCAGGCTCACTGAATTGAGCAACAAGAAATGATCAGTAAAATAA

GGACAGAAGTGCTAAAGAGAAATATAAGCAATACACAGTTATAACAACATCTGCCCCACATCCACCTGCT

TTTTGGGTTACCAGGTACACTTCCCTAGAAACCAAATGACTAAGTTTTGCCTCGAATTGATGCAAAATAG

AAAAGCATATAAAAGACAGCTCCACAGTGGATAAGAAAACATTTGTCAATACACTCAATGTTAATCTAAA

GGAGCGCTTGCTCCGGACTACTTGGACAGAATTTAAGGGAATATTTGACTAATGCACTGGACGGTGTTCG

CTGTAATGTTTCCATGTTGTCTTTTGATGGATGTCAGAGACGGTGTTATCTTGGGGATAAAGAGAGACAA

GTTTAAAAATGTAATTAATCTGTTAATTTTTATGTAAAGATTATAATAAAGCCCTGAAATTTCAAAAAAA

AAAAAAAAAAAAAAAAAA

**Primer verification**

**>Thermal gradient for optimum T_A_**

60.0°C to 50.0°C

**>Standard Curve Parameters**

**Slope:** -3.53

**R^2^:** 0.983

**E%:** 92.1%

**cDNA Dilution Factor:** 1:2

**[cDNA] range:** 5.0 ng – 0.3 ng

**T_A_:** 51.9°C

**Final [primer pair]:** 2.0 uL/rxn(i.e., 250 nM per primer)

**>Melt Curve**

Mean T_m_ (SEM): 83.32°C (0.00)


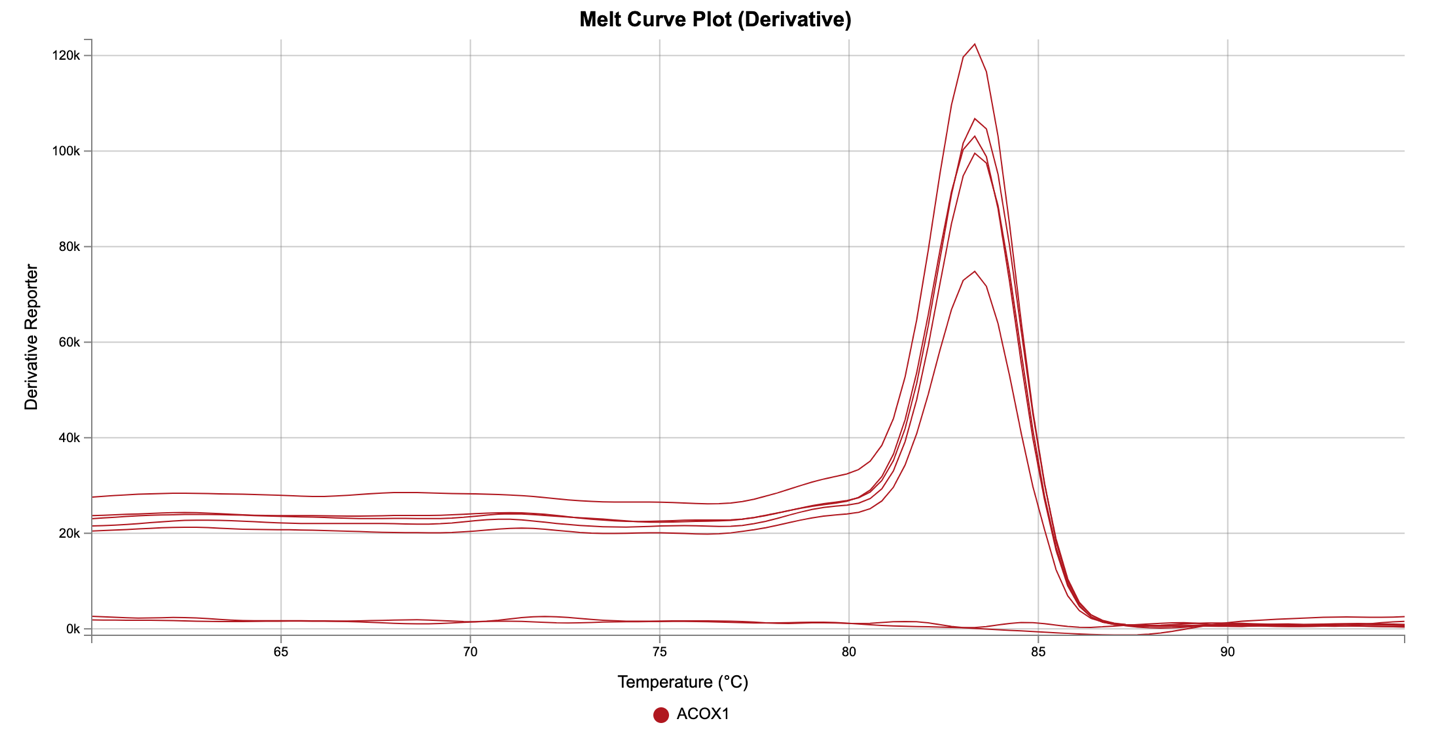


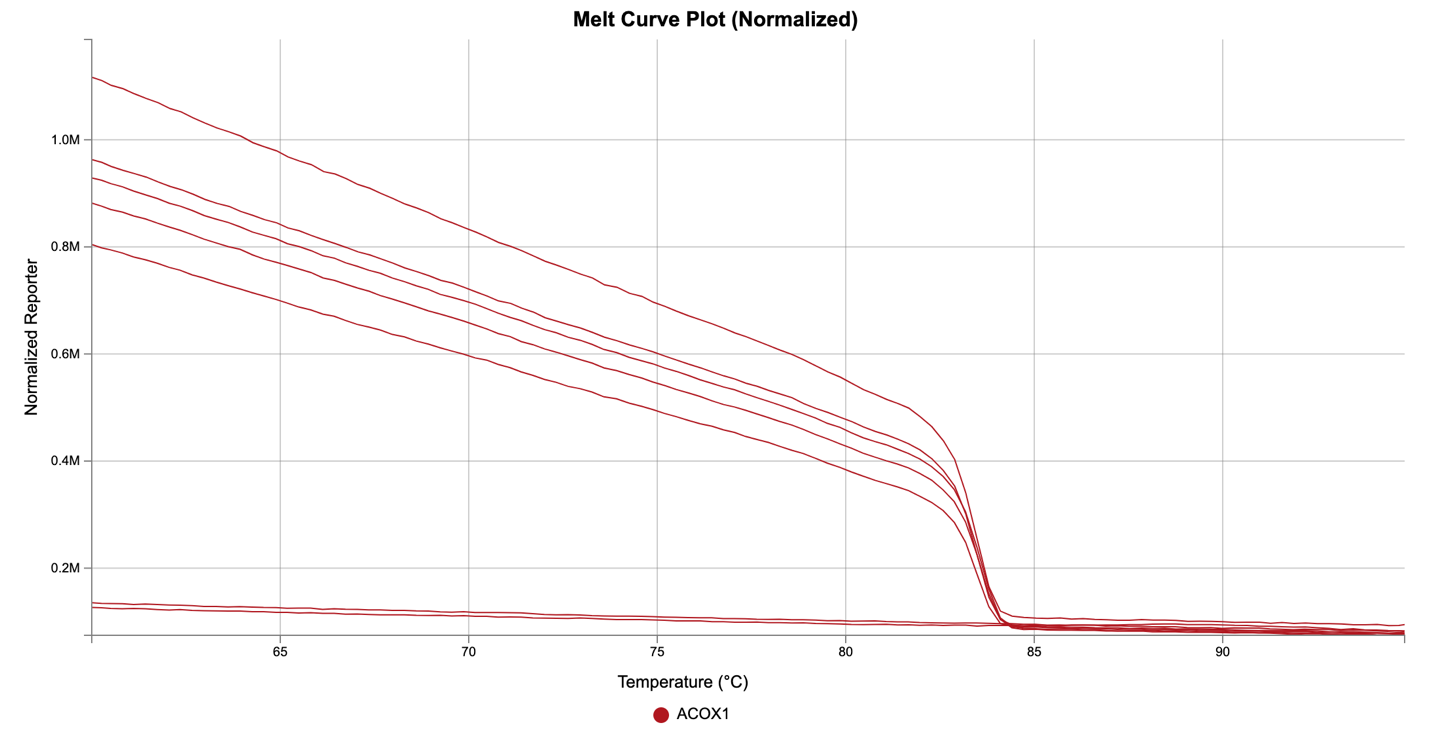


# **Text 4.** *apoa5* primer documentation

**APOA5**, apolipoprotein A5 L homeolog liver fatty acid binding protein (*apoa5.L*): target species—African Clawed Frog (*Xenopus laevis*)

**F/Left Primer (5^’^-3^’^):** CCGGAAGAGTTAAGGAAGGTTT

21 bp Tm 54.6°C 45.5%GC

**R/Right Primer(5^’^-3^’^**): GCTCAGCTTATCCTGTGTTGTG

**In sequence:** CACAACACAGGATAAGCTGAGC

21 bp Tm 56.0°C 50.0%GC

**Source:** Self-designed

**Spans exon-exon:** No

**Amplicon Product:** 125 bp

Start & End of reading frame

Predicted F primer sequence

Predicted R Primer sequence

>NM_001110744.1 Xenopus laevis apolipoprotein A5 L homeolog (apoa5.L), mRNA

CAGACAGAGTGGCCAAAGGACAGCAGGGTGCTGTGTTATACCACATATCTGGTGTCAGCAACAAAAACAG

CAGAATCTCATTTACTATTATATAGGTTCTGAAGATTCCCTTTTGTCCATTTAGCCTCAAGATGGTGAAA

ACTAGTTGGTTGTTCCTGCTGCTCCTTGTGACTCTGACAGGCAGTAAGGCTGAAAATACCCGCAGTGGCT

TCTGGGAATACTTAAGCCAGCTGACCAGCGATAAGGACAAATGGGACCTACAGCAGAATGCAGCACGAGA

GATAAACACTCTGAAGAGCAGTTTCCAGAATGGAGTTAATTATGTTGGCAAATTCCTAGGACCACTGAAA

AATGGGTTTCAGCAACGGTTGTATCAGGACACTGATGGTCTTCAAAGACTTATCAGCAGAGAATTGCAAG

AGCTCCGCAGGAAGATATACCCATACATGGATGAAGCACACCAGAAAATCAGCAAAAATCTGGAGCAGCT

ACAAAGTCGCTTACTACCATACACAAGTGAACTGAAAGACCAGGTGGAATGGGGAGCACAAGAGCTGCAC

CTTCAACTCAGACCATATAAAGATGATCTGAAAACCTGGAAAATGGATAAACTAGCTGAACATTTACAAG

ACCGTATTATTCTCCATACCGGAAGAGTTAAGGAAGGTTTCTACCCACTAGCTGAACGATTAGTGGAAGA

GATCCATCATGCCGCAGAAGAATTACATTTAAATCTTTCGCCTCATACACACACAACACAGGATAAGCTG

AGCCAACAGGTGCAGGAACTATCTCAAAAACTTACTAAAAATGCCAGGGATCTTCATGAGAAAATTCACA

AGAATCTGGATGAGTTAAAACAGCAGTTGGTGTCCTACCCTCATCAGATAAAACAACAATTCCCTAATGG

ACACAGCGCAGAACATGTAGCCCCTTATGTGGATGAGATGGCTGCTCAGGTACAGAAAGAGGTGGAAGAA

TTCCAGAGAAGCACTAAGATGCAGATAGAGGACTTTACAAGAACCATCAATAAAGAGGCTGAGGAAATGC

AAAACAAGTTATCCCCTGCATCTTGGGATTTTCAAGACAGTGTTAGCACGGTAGAGGATGTGCAAGAGAA

ACTGGATTCCTTGTGGAGGGACATAGCCCAGAGTCTGGACTAATAATGTGACTTTGAGGCACTAATAATC

CACCAATGAAATGTGAGATCACAGTAACATTAAGGCACTTGCTACTGCTACAGTATCACTAAGCAATTAT

GTTCAGTGGTCAAAGTATGATGGTACCTATTGGTACAAAATAGCTCAAGGTTTTGTTTACAAATCAGGGA

CATTCCAATTTTATAATTAAGAGATCAAAACACCACTGTATTTTTTATTTGAATTCATCCATATTTCTAA

TGGGATTAGGCTTAAAGCATTCTGTTTTATATTTATATGCTTGTTCATAAACTTGTTATATTGTACATAA

TAAACTGTTTTTGTAAAAAAAAAAAAAAAAAAAAAAAAA

**Primer verification**

**>Thermal gradient for optimum T_A_**

60.0°C to 50.0°C

**> Revalidated Standard Curve Parameters at 51.9°C**

**Slope:** -3.57

**R^2^:** 0.990

**E%:** 90.5%

**cDNA Dilution Factor:** 1:2

**[cDNA] range:** 5.0 ng – 0.3 ng

**T_A_:** 51.9°C

**Final [primer pair]:** 2.0 uL/rxn (i.e., 250 nM per primer)

**>Melt Curve**

Mean T_m_ (SEM): 80.67°C (0.02)


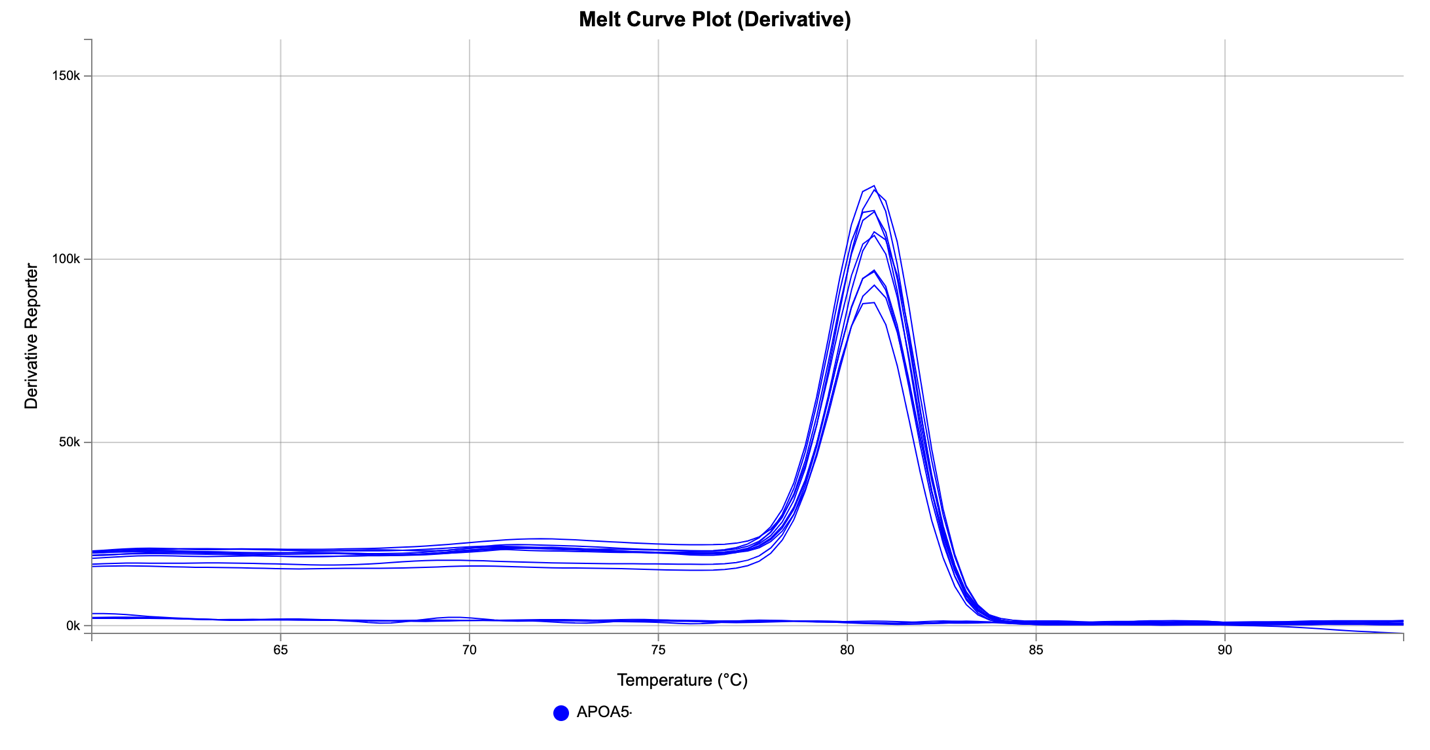


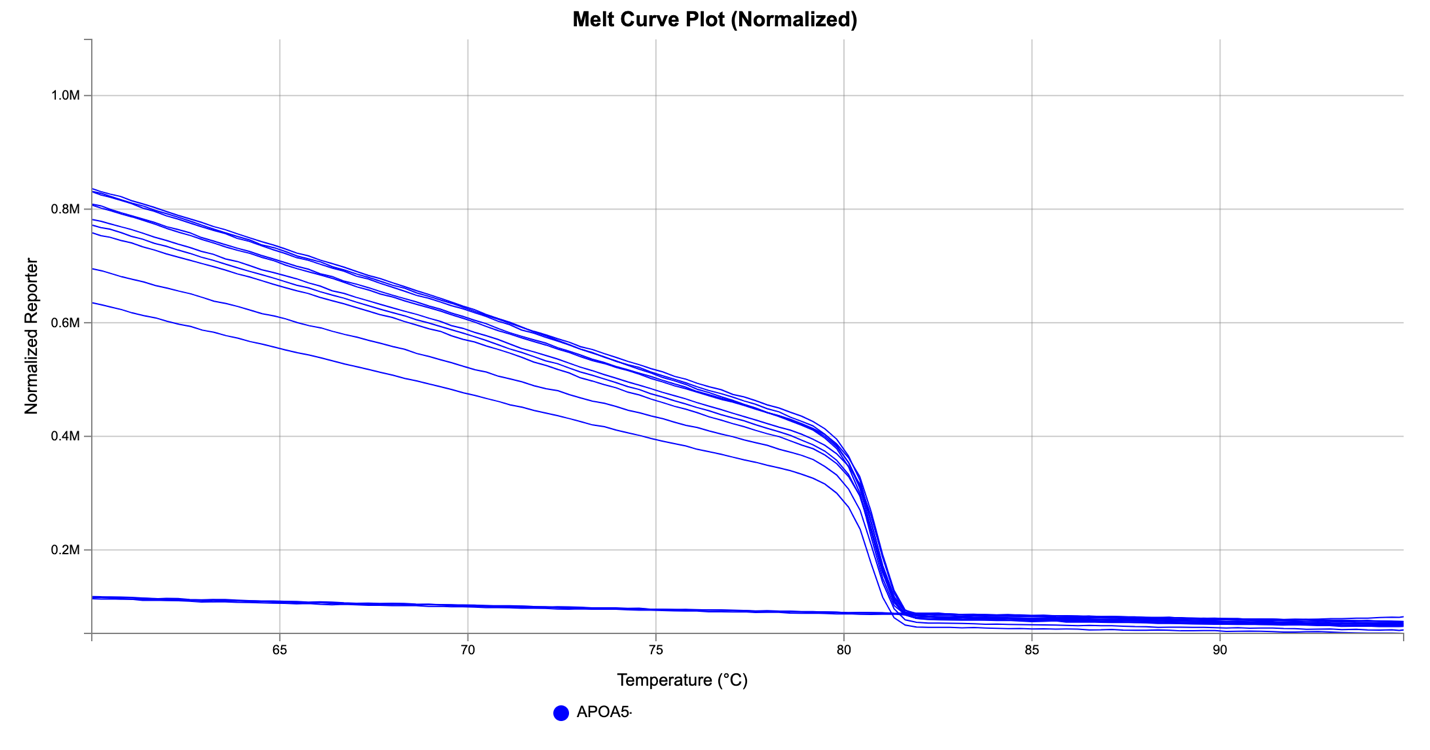


# **Text 5.** *fabp1* primer documentation

**FABP**, liver fatty acid binding protein (*fabp1*): target species—African Clawed Frog (*Xenopus laevis*)

**F/Left Primer (5^’^-3^’^):** TAAAGGGTGTCACCGAGATTG

21 bp Tm 54.5°C 47.6%GC

**R/Right Primer(5^’^-3^’^**): TCTCCCCTGTTGGTGTTTCTA

**In sequence:** TAGAAACACCAACAGGGGAGA

21 bp Tm 55.5°C 47.6%GC

**Source:** Tamaoki et al., 2018; https://doi.org/10.1002/jez.2246

**Spans exon-exon:** No

**Amplicon Product:** 129 bp

Start & End of reading frame

Predicted F primer sequence

Predicted R Primer sequence

>AF068301.1 Xenopus laevis liver fatty acid binding protein (FABP) mRNA, partial cds

ATTCAAAAAGGAAAGGACATAAAGGGTGTCACCGAGATTGAACAGAATGGCAAACACTTCATTGTCACTG

TGACAACTGGCTCCAAAGTCTTACGCAATGAGTTCACCATTGGGCAGGAAGCAGAGCTAGAAACACCAAC

AGGGGAGAAGGTCAAGTCCGTGGTAAACATGGAGGGAGATAACAAGTTAGTT

Note, the largest ORF reads 3’-5’, so above ORF identified according to the reverse compliment.

**Primer verification**

**>Thermal gradient for optimum T_A_**

60.0°C to 50.0°C

**>Standard Curve Parameters**

**Slope:** -3.53

**R^2^:** 0.995

**E%:** 92.3%

**cDNA Dilution Factor:** 1:2

**[cDNA] range:** 5.0 ng – 0.3 ng

**T_A_:** 51.9°C

**Final [primer]:** 1.5 uL/rxn(i.e., 188 nM per primer)

**>Melt Curve**

Mean T_m_ (SEM): 83.09°C (0.03)


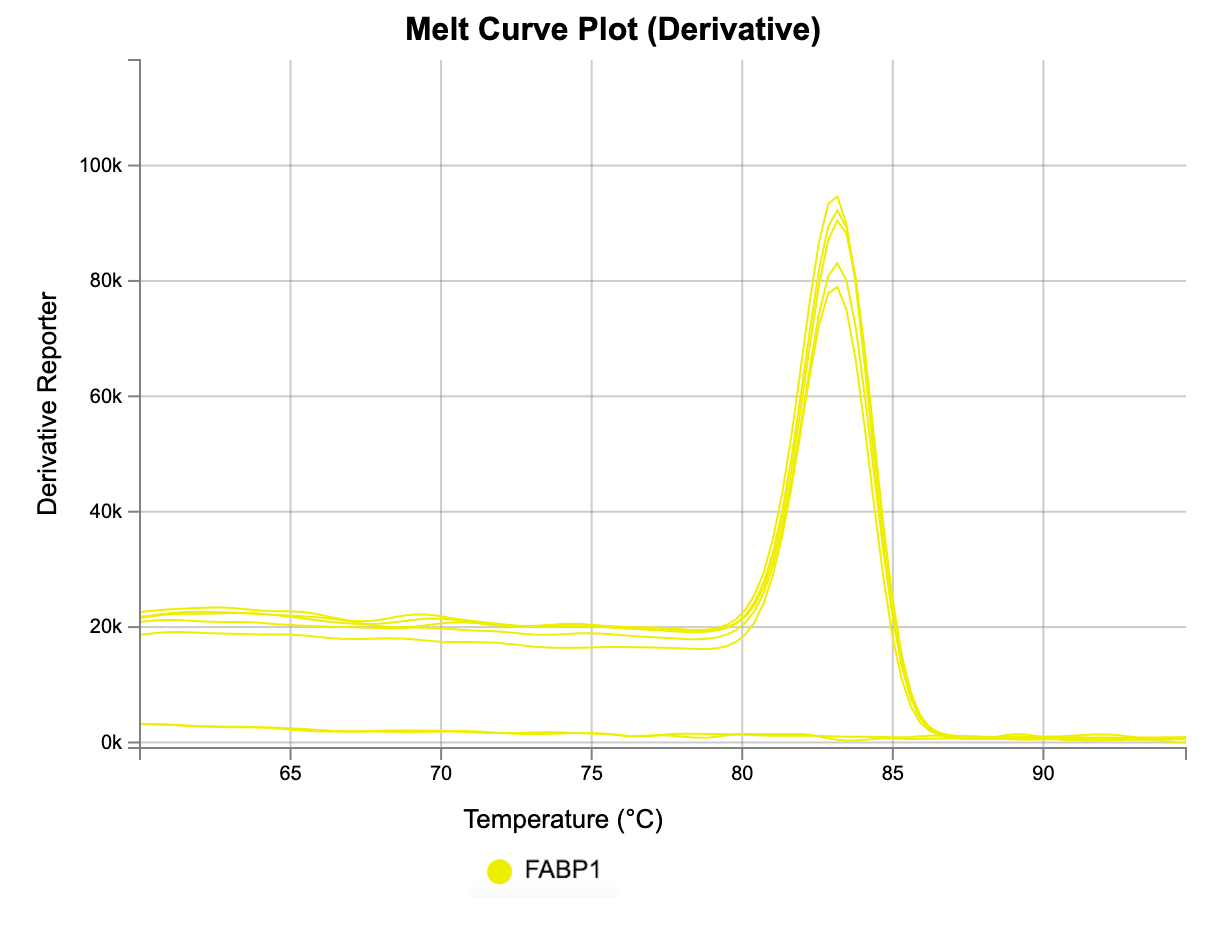


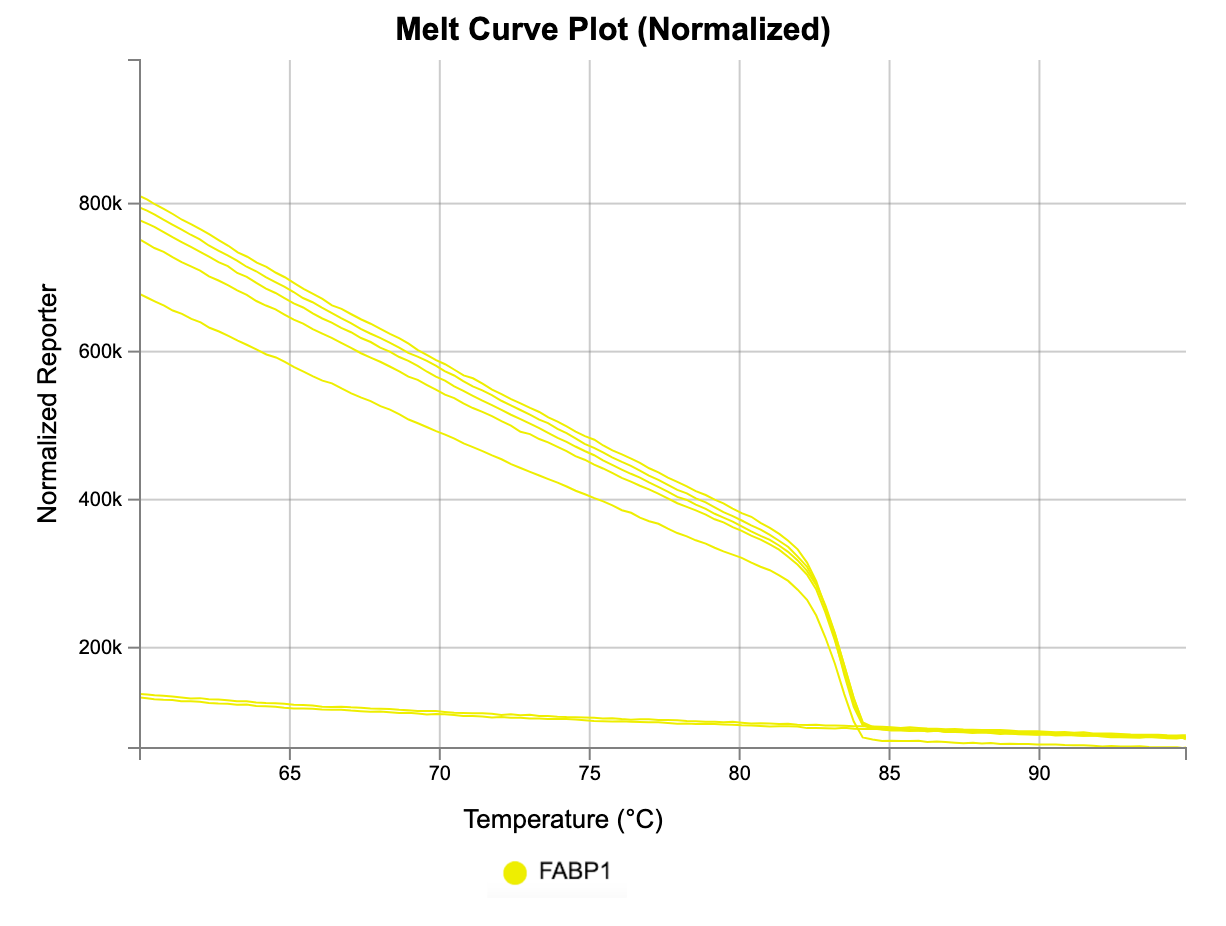


# **Text 6.** *pck1* primer documentation

**PCK1**, Phosphoenolpyruvate carboxykinase 1 S homeolog (*pck1.S*): target species—African Clawed Frog (*Xenopus laevis*)

**F/Left Primer (5^’^-3^’^):** GCAGCTGAACATAAAGGCAAG

21 bp Tm 54.7°C 47.6%GC

**R/Right Primer(5^’^-3^’^**): TGAGCCAGTGAGCAAGGTATT

**In sequence:** AATACCTTGCTCACTGGCTCA

21 bp Tm 56.3°C 47.6%GC

**Source:** Tamaoki et al., 2018; https://doi.org/10.1002/jez.2246

**Spans exon-exon:** Yes

**Amplicon Product:** 97 bp

Start & End of reading frame

Predicted F primer sequence

Predicted R Primer sequence

>NM_001086477.1 Xenopus laevis phosphoenolpyruvate carboxykinase 1 S homeolog (pck1.S), mRNA

GAAAGAGGAAACAATAAGTAGAGAATTCTTTCCTAGAAGAAAGAAGAAATCCTATTTCCTAAGGTGAAGG

AATCTACAAGTCTATAGACCCCACCATGCTTTACCAGATACAGGCAGAGCTGCAGATCACTGGCAAAGTG

AAACAGGGCAGCCTGAATATGCTCAGCCCCACAGTACAGAATTATATCCTCACTAATGCCAAGATCTGCC

AGCCAGACTATATCCATATCTGTGATGGCTCCGAGGAGGAGAATAAGAAGCTTCTCCACCTGATGGAGGA

AACTGGCATGGTCACCAGACTGCATAAGTACGAGAACTGCTGGTTGGCACGGACAGATCCACAGGATGTG

GCGAGGATTGAAAGTAAGACAGTGATTGTCACTCAGGAACAGAGAGACACCGTACCCCTGGCTAAGAGTG

GGCAGAGCCAGTTGGGGCGCTGGATGTCAGAGGAGGACTTTAAAAAAGCCTTTAAATCCAGATTCCCTGG

TTGTATGAAAGGTCGCACAATGTACGTCATCCCATTTAGCATGGGGCCAATCGGGTCCCCGCTGTCCAAG

ATTGGGATTGAGTTGACTGATTCCCCATATGTGGTGGCCAGCATGAGAATCATGACTCGGATAGGAACGG

CTGTCCTGGAGACTCTGGGGGATGGAGAATTTGTGAAGTGTCTACATTCTGTTGGCTGTCCTCTTCCCCT

GAAAAAACCTCTAGTAAACAACTGGCCTTGTAATCCTGAGCTGACTCTCATCGCCCACATACCCGAACAG

AGAGAGATTATTTCCTTTGGAAGTGGTTATGGAGGAAACTCCCTGCTGGGGAAAAAGTGCTTTGCCCTTA

GAATTGCCAGCCGAATCGCCAAAGAAGAAGGGTGGCTTGCTGAGCATATGCTGATTTTGGGTATCACCAA

TCCTCAAGGAGAGAAGAAGTATTTTGCGGCAGCATTCCCCAGTGCCTGCGGGAAAACAAATCTTGCTATG

ATGAAGCCTTCAGTTCCAGGCTGGAAGATTGAATGTGTCGGAGATGACATTGCATGGATGAAGTTCGATG

AACATGGAAACCTAAGGGCCATCAACCCAGAAAATGGCTTTTTTGGGGTCGCACCTGGCACATCTGCAAA

GACAAACCCATATGCCATGGAGACCATTCGAAATAACACAATTTTTACCAATGTGGGAGAAACCAGTGAT

GGAGGCATTTATTGGGAGGGCATTAACAAGTGTTTGGATCCAGGAGTCACCTTGACCTCTTGGAAGAACA

AGGAATGGACCCCAGAGAATGGGGAACCTTGTGCTCACCCTAACTCCAGGTTCTGCACACCTGCAAGCCA

GTGCCCAATCATTGACCCTGAGTGGGAGTCCCAGGAAGGTGTACCCATCGAGGGCTTAATTTTTGGAGGG

CGTAGGCCAGCAGGTGTGCCACTGGTCTATGAGGCTTTGAGTTGGCAACATGGTGTTTTTGTTGGATCTG

CAATGAGGTCTGAAGCAACGGCGGCAGCTGAACATAAAGGCAAGGTTATCATGCATGATCCATTTGCCAT

GAGACCTTTCTTTGGTTACAACTTTGGGAAATACCTTGCTCACTGGCTCAGCATGGAACACTATCCATCC

ACCAAGCTGCCAAAGATCTTCCACGTCAACTGGTTCCGTAAAGACAAAGAAGGCAACTTCCTGTGGCCAG

GCTATGGGGAGAACATTCGCGTCCTCGAGTGGATGTTCAAAAGGATCAATGGAGAAGACTGCGCCAAAGA

GACGCCCATCGGCTTCATCCCGGCTGAAGGTGCCTTGAATCTGAAAGGACTTGGTGATGTCAACATGGAA

GAACTTTTTGAAATATCTAAGGAATTCTGGGAAGAGGAAGTGAAGGACATTAAAAAATATCTAGATGACC

AAGTCAATGCTGATCTACCTTATGAGATTGAAAGGGAGTTGTCTTCATTAGTGGAAAGGCTTAAGCAATT

GTGAACAAGAGTGGTAGGATCTGACTTAGGTCTTAGACTTTCTAAAATAGAAGTGGGCAGCATGCCGGCA

GCCTTGGCATAGTGTATTATAATTGGTGATGGTTGGGTCACTATTATATAAGCTGTGAACAAATCGTTTT

AGAGTAGAAGTTTAGGATAAAATTATTTTGTCTTCCTTACTCAATCACAGTCCTCGGGGTGATTACACTA

TGTGGGGATCAAATCAGATTAGTAGGGTAGACCCCTTGTACTGTATTTTTAGGAACACTGTCTTACTGTA

AAGCACTTTCGTGTTGATGTGTACCTTCTGTTTCCTTTGGTAGGAGAGTTTTTTTAGTGTGAATGTACAG

TGCAAGAGTTTAGGGTTTAGTGTGTGCCTCATCTAGTGAAATAAGACTTGTGAGTCAATGCAAATCATCA

CTGAAACAATGAATTCATACAAAGGGGACACAATAAAGCTAAAAATAAATATATGAATGAGGAAGAGTGT

GAAAAACTCTTTGGCTGTGTAATTCAGAGGAAGCCGAGGAGGAGGAGAAAGGGCAAGTAATATACTAGAC

AATTAGGAATTTTTTTTAAATTAAATATACAAAAATTGTATATCAACTTTTTATATCCTATACATTATGG

TATATGTTCTATTGTACCTGTTTTTGTTCAATGTGAAATTTCAATACAATTTTTATAAAAAAAAAAAAAA

A

**Primer verification**

**>Thermal gradient for optimum T_A_**

58.0°C to 51.0°C

**>Standard Curve Parameters**

**Slope:** -3.51

**R^2^:** 0.985

**E%:** 92.7%

**cDNA Dilution Factor:** 1:2

**[cDNA] range:** 5.0 ng – 0.3 ng

**T_A_:** 53.0°C

**Final [primer pair]:** 2.0 uL/rxn(i.e., 250 nM per primer)

**>Melt Curve**

Mean T_m_ (SEM): 81.21°C (0.02)


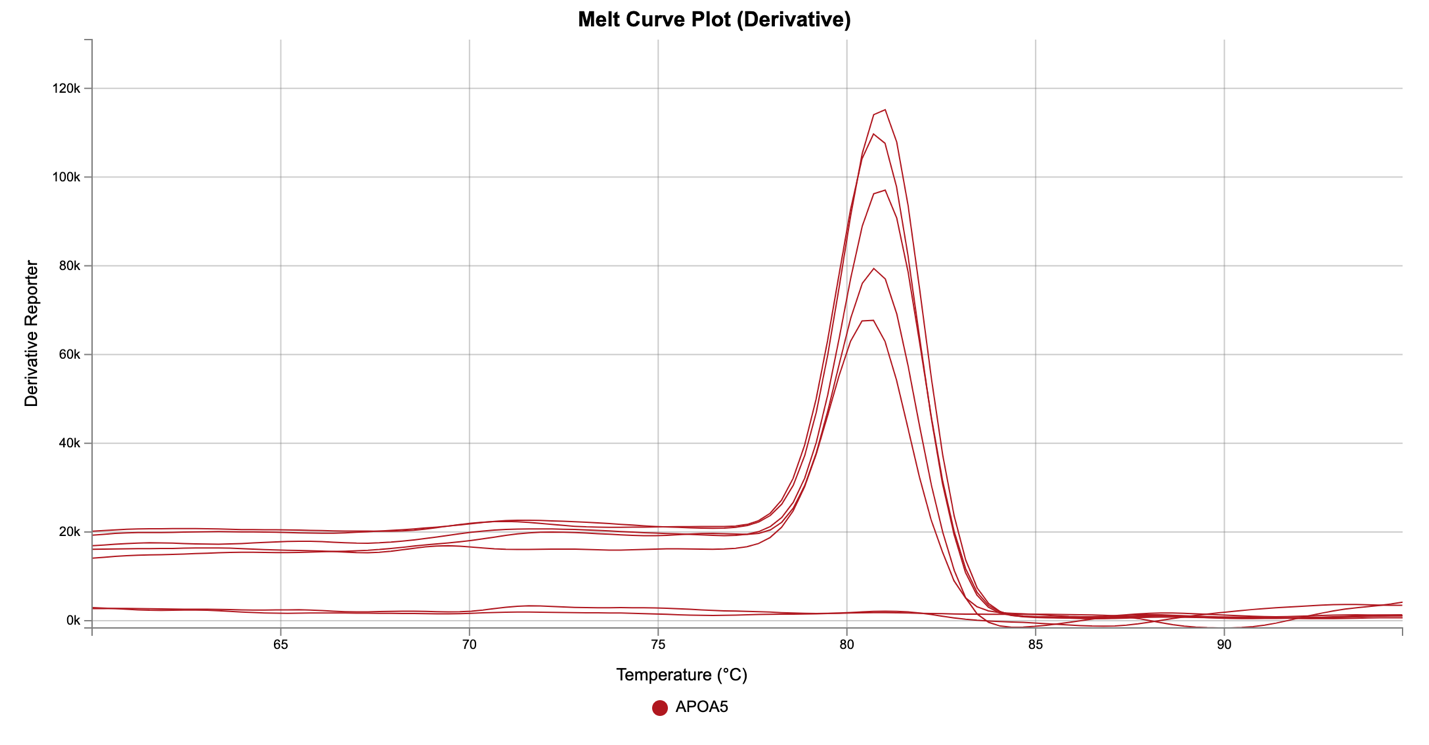


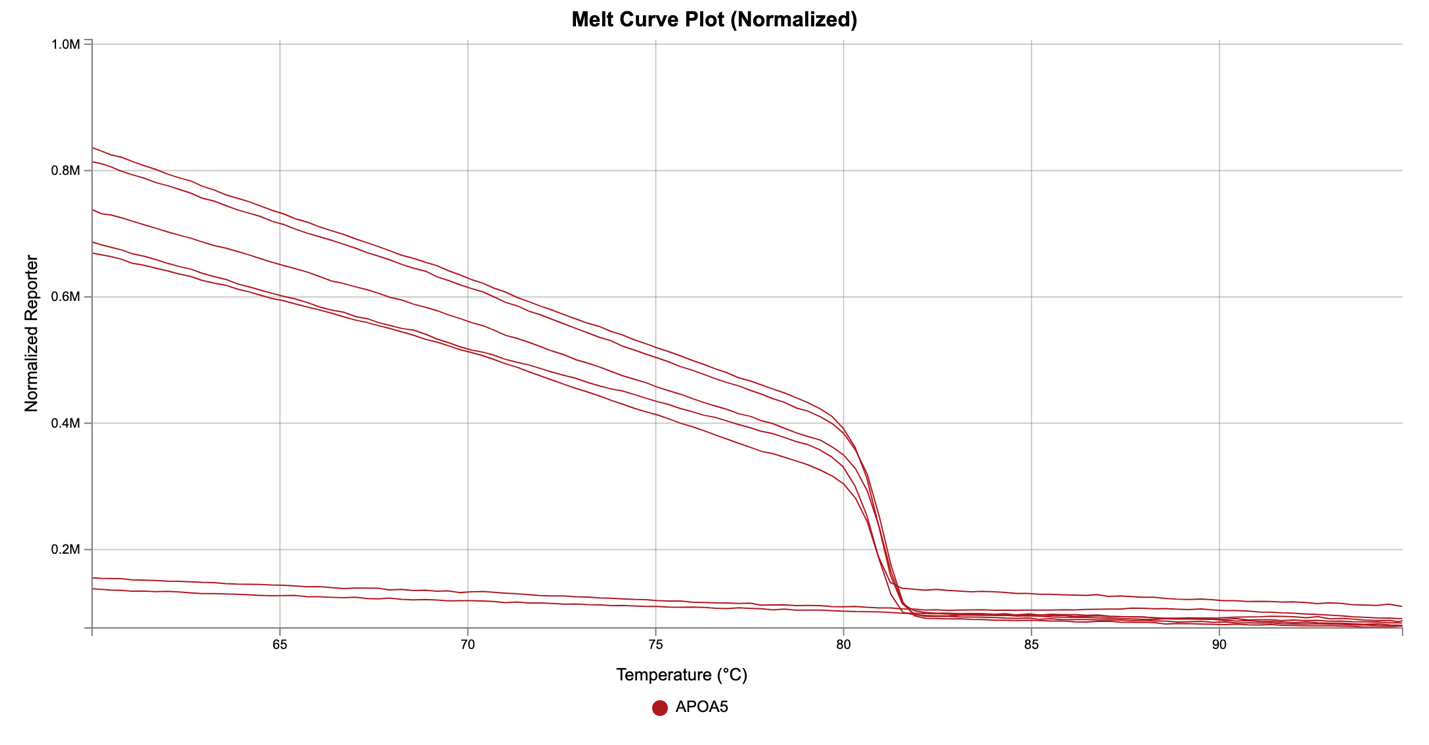


# **Text 7.** *sub1* primer documentation

**SUB1** homolog, transcriptional regulator (*sub1*): target species—African Clawed Frog (*Xenopus laevis*)

**F/Left Primer (5^’^-3^’^):** AGCAGGAGAAATGAAGCCAGG

21 bp Tm 57.4°C 52.4%GC

**R/Right Primer(5^’^-3^’^**): CCGACATCTGCTCCTTCAGT

**In sequence:** ACTGAAGGAGCAGATGTCGG

20 bp Tm 56.8°C 55.0%GC

**Source:** Mughal et al., 2018; https://doi.org/10.1038/s41598-017-18684-1

**Spans exon-exon:** Yes (E4-E5)

**Amplicon Product:** 80 bp

Start & End of reading frame

Predicted F primer sequence

Predicted R Primer sequence

>XM_018266540.2 PREDICTED: Xenopus laevis SUB1 homolog, transcriptional regulator L homeolog (sub1.L), transcript variant X2, mRNA

TTACCAATCACAAGATGGCCGCCAGAATCACAAGATTCTCGAGATACCATATAATTTTTATGACAATTTC

CCGCCGCTCTCCCGCTACTGGCGTTACGTCACTTCCGTTGAGGAGCGATCACAGAACCGCACGTGGTGCT

AGCAAACAGAATGCCAAAATCAAAGGAGATCGTGTCTTCAAGCTCATCTGGAAGTGATTCTGATAGTGAA

GTTGACAAAAAGGTGAAGAGAAAAAAGCAACCACCTCCAGAAAAAGAAAAGCCAGTGAAGAAGCAGAAAA

CAGGGGAAAGTTCTAAGGGCGGAGCTTCTTCAAGGCAAAGCAGTGGCCCTGAGGATAATATGTTCCAGAT

CGGAAAAATGAGATTCGTTAGCGTTAGGGACTTCAAAGGAAAAGTCCTGATTGACGTCAGAGAATACTTC

ATGGATCAAGCAGGAGAAATGAAGCCAGGAAGAAAAGGTATTTCTTTAAATCCTGAGCAGTGGAACCAAC

TGAAGGAGCAGATGTCGGATATTGACGACGCAATAAGAAAACTGTAGAATTCTACCTACGCCTTGACGAA

ACCATGTTTTCATTTTAAGCACTCTTTTTACATTTGCTGTTTTACTTTTTTTTTTTTATACACGCTATTG

TACATTTGGATTGTAAAACAATTTGTCGGCTATTACACTTAATCTCTTCACAATTGCGTAGAAATATACA

CCACGGTGCAATGTGAAACATGTTTTTGTTTAAAAGTTACCCATTACAAATTATCTTTAATTGTTTGAAA

CAGATGACACATTTAAACACTCACTTTTTTTCCCCCTTGATCCGTAAAGGGCAACTTAAAAGGACTGTGC

AGACTCCACACATTTTGTTTATACTAATGCGTAGGGCTACTGTACCACTTTCCACAGCCGGCAGCCTTAA

GCAGCTTAATTTACAGACTTCTTTTTTCCCCTACAGTGTTTTCAACATTTCTCCTCCTTGATGCCCCTAC

ACTTATGACACTGGAGAAACACAAGAGTTAAGTGCAATGTAAAGCAAGGGTTAAAATGCAACATAGCCAT

GTGCATTAGTATAGACAAAATTAAGAGAATTTTTGCTGGGCCTTAAGTTTCCCTTGAACACAATGATGAT

GTT

**Primer verification**

**>Thermal gradient for optimum T_A_**

60.0°C to 53.0°C

**>Standard Curve Parameters**

**Slope:** -3.39

**R^2^:** 0.999

**E%:** 97.3%

**cDNA Dilution Factor:** 1:2

**[cDNA] range:** 5.0 ng – 0.3 ng

**T_A_:** 53.5°C

**Final [primer pair]:** 1.5 uL/rxn(i.e., 188 nM per primer)

**>Melt Curve**

Mean T_m_ (SEM): 79.22°C (0.02)


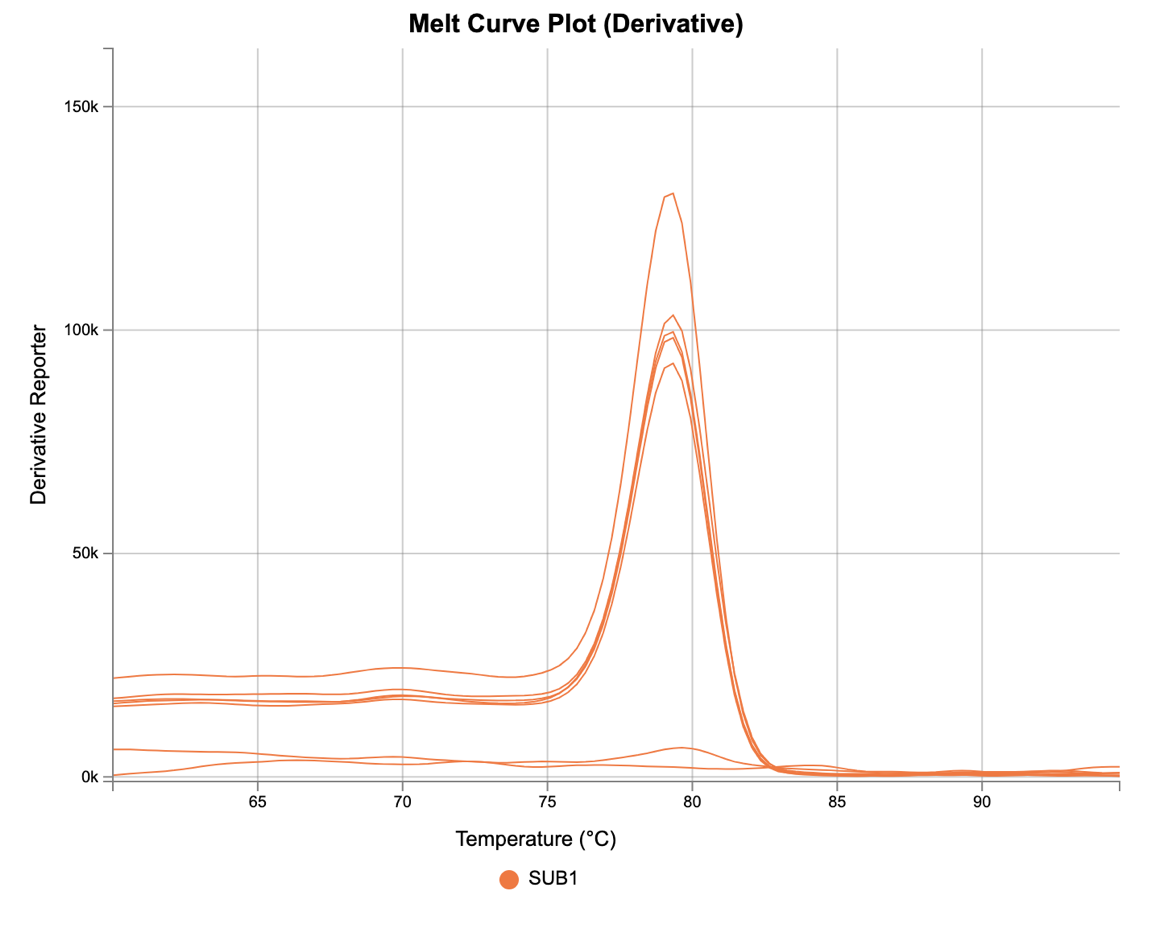


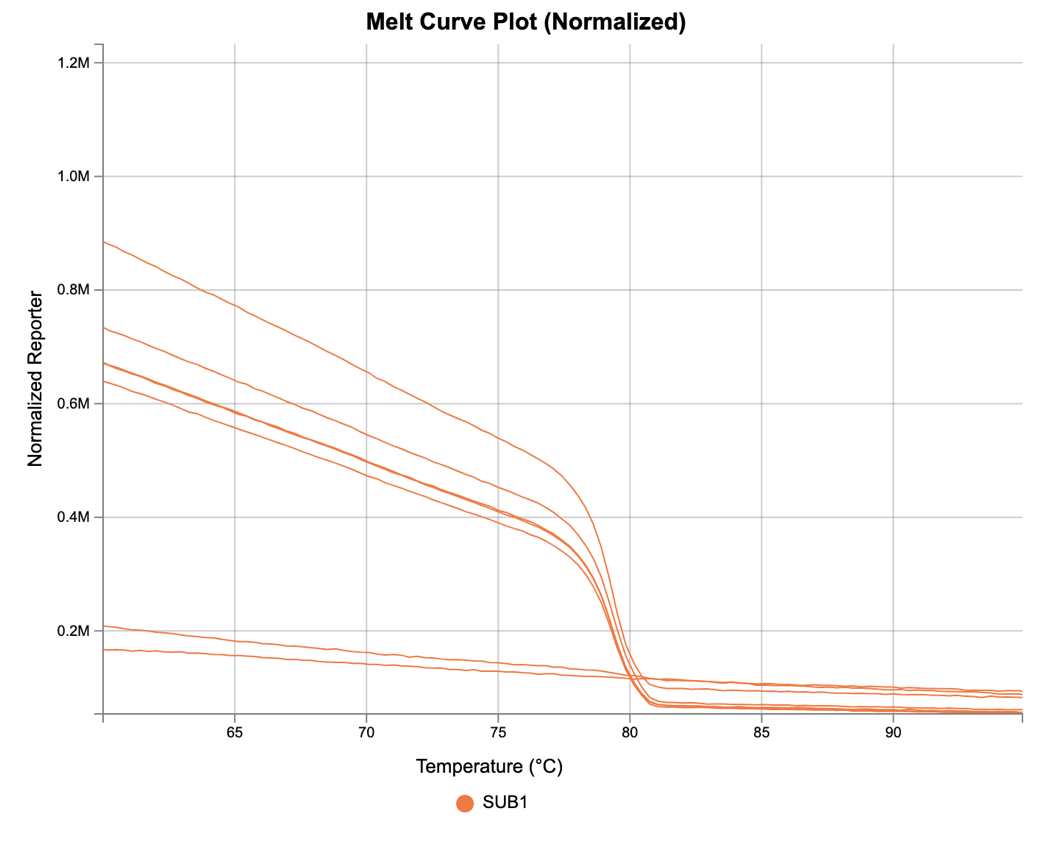


# **Text 8.** *xPPARa* primer documentation

**xPPARa**, Peroxisome proliferator activated receptor alpha L homeolog (*ppara.L*): target species—African Clawed Frog (*Xenopus laevis*)

**F/Left Primer (5^’^-3^’^):** GCCGAGAAGACGTTAGTCGC

20 bp Tm 58.1°C 60%GC

**R/Right Primer(5^’^-3^’^**): GTGAAGCCGGGGATGGATTT

**In sequence:** AAATCCATCCCCGGCTTCAC

20 bp Tm 57.8°C 55%GC

**Source:** Self-designed

**Spans exon-exon:** No

**Amplicon Product:** 146 bp

Start & End of reading frame

Predicted F primer sequence

Predicted R Primer sequence

>NM_001095362.1 Xenopus laevis peroxisome proliferator activated receptor alpha L homeolog (ppara.L), mRNA

GGCGAGAGCAAGGACGCACCGGGGAGAGAAACAGGGACCGGGGCTCCAACCAGCGACCCGCCGACCCCCC

CCGGACACCGGTCTCCCAACACGAGGGAACTGAATTCTCAGCAGCGTGAAAGTCAGAACCGCAATGACCC

GGCAAGTGTCTACTGACGGCGCCTCATTGGCCATGAAAGGATAAGATTCCAGGAACTGATTATTATGGGA

GTTTGAGTTCTTTTTGTTTGGAGTCTCGGAAACCTGAACTAAACAGATTGATCTAATGAAGCTTCCATAG

AAGTAACGACTGATTAACTATGTGATGATTTCGGGTCAGACTTGCTATGTCCGCCATCATGGTAGACACA

AACAGCGAGTTGTGCACTTTGACCACTTTGGATGAGGACGATCTAGGGATTCCGCTGTCCGGAGAGTTCC

TGCAGGACATTGGGGACATTCAGGACATCACTCAGACAATTGGGGACGATGGCTCCATGGCTTTTGGTGC

CTTGGGAAACAGCCCTGGGTCAAACGGCTCTGTCAGCACCGACCTTACAGACACTTTATCTCCTGCGTCG

AGTCCGGCTTCCATCACCACCCCCGCGGCACCGGGTAGCGCCGAGGACGCGTCCTGCAAGTCGCTCAGCC

TCGAGTGCCGGGTGTGCGGAGACAAGGCCTCCGGGTTCCATTACGGAGTTCACGCTTGCGAGGGGTGCAA

GGGCTTTTTCCGGAGGACAATCCGACTCAAGCTGGTGTACGACCAGTGCGAGCGAATGTGTAAGATACAG

AAGAAGAACCGCAACAAGTGCCAGTATTGTCGCTTCGAAAAGTGCCTTAACGTCGGGATGTCCCACAATG

CAATACGATTCGGGAGGATGCCGCGGTCGGAAAAGGCGAAACTGAAAGCGGAAGTCCTGATGTGCGACCA

GGACGTGAAGGATACGCAGATGACCGACCTCCTCTCACTCGCCAGGTTCATTTACGAAGCCTATCTAAAG

AACTTCAACATGAACAAAGTTAAAGCAAGAGCCATCCTGATTGGGAAAGCCAGCACTCCGCCATTTGTTA

TACACGACATGGAGACGCTGTGCATGGCCGAGAAGACGTTAGTCGCGAAGCTGGTGGCCAACGGCATCCA

GAACAAAGAGGCGGAGGTGCGGATCTTCCACTGCTGCCAGTGCACCTCGGTGGAGACGGTCACAGAACTG

ACAGAGTTTGCCAAATCCATCCCCGGCTTCACCGAGCTGGACCTCAACGATCAAGTGACTTTATTAAAAT

ACGGGGTCTACGAGGCCATGTTTGCCATGCTGGCGTCCGTCATGAACAAAGACGGGATGCTGGTGGCGTA

CGGGAACGGTTTCATCACGCGGGAGTTCTTGAAGGGCCTTAGGAAACCGATTGGAGACATGATGGAGCCT

AAGTTTGAATTTGCCATGAAGTTCAACGCACTGGAGCTGGACGACAGCGATCTCTCTCTGTTTGTAGCTG

CCCTCATTTGCTGTGGAGATCGCCCCGGATTGGTGAACGTTTCCAGTATAGAGAAAATGCAGGAGAGCAT

CGTCCATGTCCTTAAACTCCACCTCCAATCAAATCACCCCGACGACGGCTTCCTCTTCCCTAAACTGCTG

CAGAAAATGGCCGACCTGCGGCAGCTGGTGACGGAACACGCGCAGTTGGTGCAGACCATCAAAAAGACTG

AGACGGACGCCGCCCTCCATCCGTTACTGCAGGAAATCTACAGGGACATGTACTGAGCCCTCTGCGAAGA

TATTTTTTTTATTAGGATTTTTTTGTTTTTTTTTAATTTTCTGGACGGGAGCGAAATCTGCAGATTTAGG

GCTTATTTTCCTGTGCCTTCTCTTCCAGCCGCGGCGACAATTCCTAACACTGCACTACGGTTTCTTCAGC

AAATGTAACGCAGACAATAAGCCGGGGGTGGGGGGGCGAACACTACAGAATATTTGGTTTTAAATATATA

CTAATATTTTTCTCTTTTTATGTATTTTGACTGAAAAAACGCGGTGTTTATAGTGTGTATACCGTACGCC

GCGCTTTTCATGACCTAAAGATGATATTTAACATGTTGCAGTTTGATTGGGAAACAGATTTGATTCAGGG

TCATGAGAATCCAACCCCCAAACTAATAAATATTACATTTCCCAGCATCGTTGGAAGGCTTAAAGGGATA

CTGTCATGGGGAAAAAAAATTTTTTCAGGATGAATCGGTTGGTGGTGCTGCTCCATCGGAGTTCTGCGCT

GGGGTCCATTTCTCGAGGGAGCAGGCAGATTTTTTTGTATTTGGTTTTGGGATCTGACATGGGGCTGGGC

ATGTTGTCGGTTTCCCGGCTGCCCCTAGTCATGTGGCTTGTGCTCTGATGGACTTCGGTCACTCTTTGCT

GCTGTGCTGCAGGTTGGAGTGATGTCACCCCCTCCCTTTTCCCCCCAGCGGCCAGGCGGGAGAGCAATGG

GGGGGTGGCCAGATTGCAGCTCCCTAGCACAAGATGGCAGCTGCCTGGTGGATCTAAAGGTGGCCATACA

TGGATAGATCCGCTCGTTTGGCGATGTCGCCAAACGAGCGGATCTCCCTCCGATATGCCCACCTTGAGGT

GGGCAATATCGGGCAGATCCGATCGTGGGCCCTAGGGCCCAACGATCGGATCCTAACATTCGGCAAACGG

GCGGTCGGATCGCGGGACCGCATCAACAAACAGATGCGGCCGTGATCCGACGGGATTTTCTGTCCCATCC

GATCGAGATCTGGCCGACTTTCGGCCAGATCTCGATCGGGGAAGCCGGCAGCTTTTATCGGCCCGTGTAT

GGCCGCCTTAAGAACGGCACTGGATGGTGGGATCCAGGTCCCACTGAGACACATTCGGTTGCATTGGGAA

GGAGAGGCAGCAGCCTGCCAGGGGGCGTTTCTCTCCTAGAGTGCAGGCACGGGTCACGTGACTTGGGGCG

GCTGGGAGGTTGGCGGGATGTCTGGCCCCATGTCAGATTTCAAAATTGAATATGGAGGGGTCGGTTTGCT

CTTTTGGGAGGTGGATTTCGGTGCGGAGTTCTGCTGGAGTGGCACTATTGACTGATGGGTTTTGGTAAAA

ACATGTTTTTCCATGACAGTATCCCTTTAACAGCTGGAAAATAAATAAAAGTGCCCAGGAGTATAGTCGC

TGAATCCGATTAATCTAATAAGGGAGGGTTTTTGACAGAATACCCATTTTTTTCGCCTATTTCAGTGGCA

ATTAAAGGGACAGGGATCCTTTGCAAGACGGGGTTCCGTTTTAAAGGGAAAATATACCCTAAGCGGTATT

AACGTCATTTTCTTGAAATTCGGAAACTGGGGCGTAGGCGAATGCATAGTCTCGCCTAGTTGTATTCAGT

CATATGTGCGGGAGCAGCCATTCACGCTGTGCCCTGATAATCACCAACGCAACCCCTTAGAAGGGATGTT

GGTCAGTATCCATAGCAACCAGGTAGCAGTTTGAATGTAAAACGGGGGAGCCACAGAACCGGAATAGAAA

TACACAACTTTTTAAAAAAACATTTCAAGGTATTCATAAAAATACAAGGTGAACTTCCCCCCTTAAAAAA

GTAAATAACGCTCTGGACATGGTTTCAGAATAGTGATGAGCGGAATACCCCTCCCCTTTGGTGATGTCAT

TGAGCGGAATACCCCTCCCCTTTGGTGATGTCATTGTGCCAACTCCTTACTAACTGTAAGTCAGCGCCAG

GTTCACAGCGTCCTTATTTATGTATTTTCGAAGGCTACCGTATATACTCGAGTATAGGCCGAGGTACCTA

GTTTTACCTCCAAAAACTGGGAAAGCTTATTGACTCAAGTATAAGCCGAGGGTGAGAGATGCAGCAGCTT

CTGGTAAGTTTCGGTCTCGTTTTTGGATGACTATTCTTGGGCGCCGGCTACTATTCTAAGGCGCCGGCGA

ATATTCTTAGGCTCCGGCTACTATTCTAAGGCGTCGGCGAATATTCTTAGGCGCCGGCGACCGTTTTTGC

GCTTGACCCGAGTATAAACCGAGGTAGAGTTTTTCAGCATATTTTGGGGGCTGAAAAACTCGGCTAATAC

TCGAGTATATACGGTACATTTCTGATTTTTTCATAATTTTGTACAGGAATCTCCGAAGCCTCAAATAACT

CCAGACTAGGGGGGGTGGGGGAGACTGGAGTTTATAAGATTTTATTTTTGGGGGGGAATATATTTTCTTG

GATACATTTTTAGATTTTATGGTAACGAACCACTATAGTACGAATGAACAAAAAAGAGAAACAATTTTCC

GTCCGCATTGGGCTACTAAATATTTTTGAACTGACTCGCCACTTCTCCTTTAAGATAATCTCGGACAGAT

ACATTTGGAATGTATTGATACAAAATGCACTGCTTCTGACATAGAAATGGTATTTTTTTTCTCAGGGATC

GCCTTTTTTTTGTCAACTTTTTGCCTTTTTTTCTAGTTCTTAGCTTTAGCGATGTTTCTCGTCAGTCCAA

CAAGCACCTTCATGAGATGTTCTGAATGTTTCTGTGATCTTCTTAGTCACAAGCTGCTCTAGAAAATCTG

TCTTTGATAAATCTGTTTTCCCGTAAAAAAAAAAAAAAAAAA

**Primer verification**

**>Thermal gradient for optimum T_A_**

62.0°C to 52.0°C

**>Standard Curve Parameters**

**Slope:** -3.70

**Slope** **for dynamic range:** -3.42

**R^2^:** 0.994

**R^2^ for dynamic range:** 0.999

**E%:** 86.1%

**E% for dynamic range:** 96.2%

**cDNA Dilution Factor:** 1:2

**[cDNA] range:** 10.0 ng – 0.6 ng

**[cDNA] range for dynamic range:** 5.0 ng – 0.6 ng

**T_A_:** 52.7°C

**Final [primer pair]:** 2.0 uL/rxn(i.e., 250 nM per primer)

**>Melt Curve**

Mean T_m_ (SEM): 87.41°C (0.02)


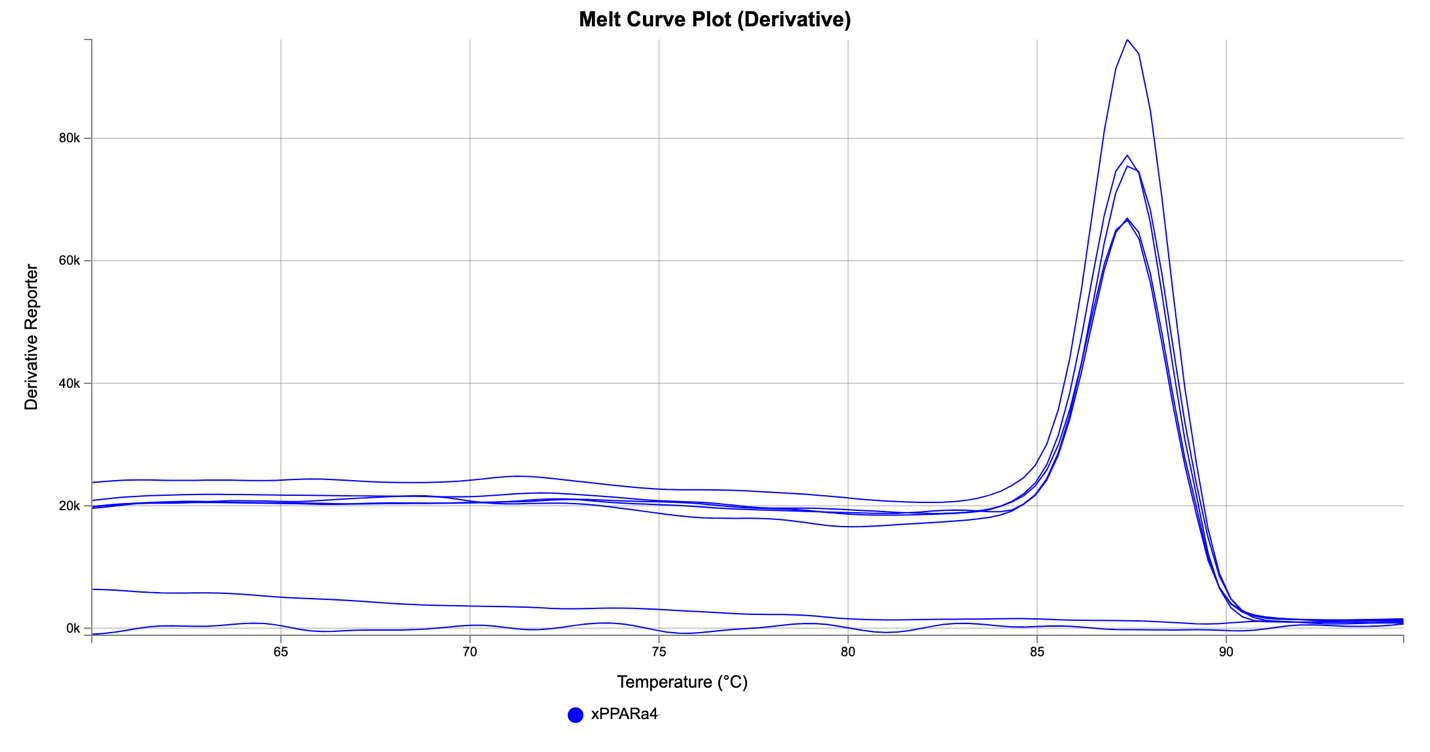


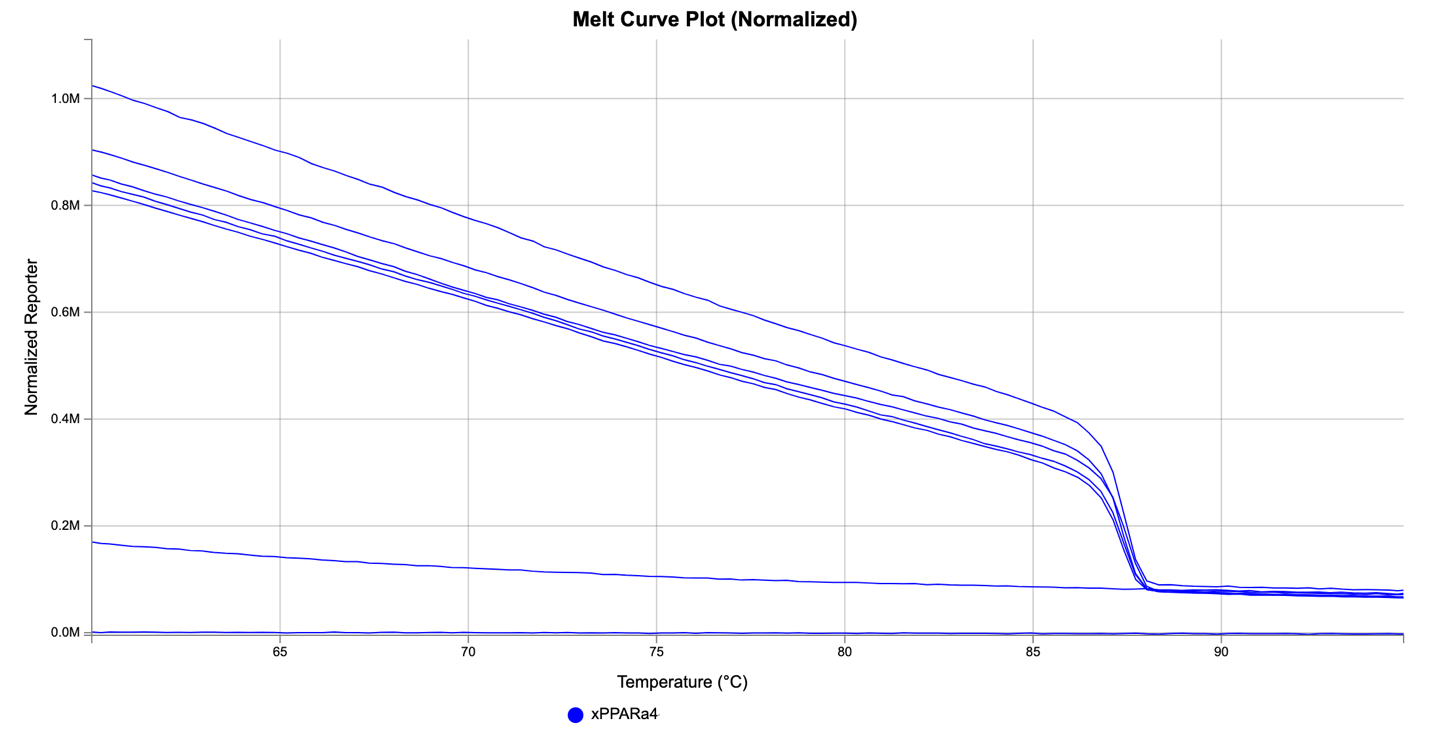


# **Text 9.** *xPPARb* primer documentation

**xPPARb**, Peroxisome proliferator activated receptor delta S homeolog (*ppard.S*): target species—African Clawed Frog (*Xenopus laevis*)

**F/Left Primer (5^’^-3^’^):** GCTTCGGCACACTCTACCTC

20 bp Tm 57.8°C 60.0%GC

**R/Right Primer(5^’^-3^’^**): AAGACTGCGCAGGAACTCAC

**In sequence:** GTGAGTTCCTGCGCAGTCTT

20 bp Tm 57.7°C 55.0%GC

**Source:** Self-designed

**Spans exon-exon:** No

**Amplicon Product:** 155 bp

Start & End of reading frame

Predicted F primer sequence

Predicted R Primer sequence

>NM_001087841.1 Xenopus laevis peroxisome proliferator activated receptor delta S homeolog (ppard.S), mRNA

GCAGGAAGGGAGGGGTGACGGAGCTCATCATTTAAAGAACTAAACAGTTTCACATCACGTCACATTGCTC

ACAGATGAAAGAAGAAATCCCACCTCGTTCTCCAATCTTAGATGAGCAGCCTTCCACCCCTCTGGAGCAT

CAGGAGACATCACAGAGTGTCGACTGCAAGATCTGTGGAGACAGAGCCTCAGGGTTCCATTATGGGGTTC

ATGCGTGTGAGGGTTGCAAGGGATTTTTCCGTCGCACCATACGGATGAGATTGCAGTATGAACATTGTGA

CCGCAACTGTAAGATCCAAAAGAAAAATCGGAACAAATGTCAATACTGCCGATTCAATAAGTGTTTGAGC

CTTGGCATGTCCCACAATGCCATCAGATTTGGTCGGATGCCGGAGTCAGAGAAGAGGAAGCTAGTGCAGG

CACCAGTATCTGATTCAGCTGCTCCTGATTCTCCAGTGTCTGACTTGGATGTTCTATCTCAGCTGATCCA

CTCTTCCTACATGAACACTTTTACCATGACTAAGAAGAGAGCACGGGACATCCTGACTGGCAGGAACAGT

ATCTCGCCCTTTGTCATACATGACATGGATACCCTGTGGCAAGCAGAGCAAGGGACCGTGTGGGAACAAT

TGCCAACCCAAAACCTTACAGGAACAGAGATTGGAGTTCATGTATTTTATCGTTGCCAATGCACTTCTGT

GGAGACTGTTCGTGCACTTACTGACTTTGCCAAAAGGATTCCTGGCTTCGGCACACTCTACCTCAATGAC

CAGGTCACACTTCTAAAATATGGAGTCCATGAGGCTATCTTTTGCATGCTGGCCTCCCTTATGAACAAAG

ATGGGCTGCTAGTTGCTGGAGGGCGTGGATTTGTTACCCGTGAGTTCCTGCGCAGTCTTCGCCAGCCATT

TTGTCATATCATGGAGCCCAAGTTCCACTTTGCCTCCAAGTTCAATGCCCTGGAACTTAATGACAGTGAT

CTGGCACTTTTTGTGGCATCCATCATCTTATGCGGTGATCGTCCAGGCCTCATTAATCCCTCACAGGTAG

AGGACATCCAGGAGGGTATTCTAGGTGCTCTTAGACGTCATCTCAAGGCATCTCACACAGATGCTCCTTT

TCTGTTCCCTAAACTTCTGCACAAGATGGCTGATTTGCGTCAGTTAGTGACTGAGCATGCTGAGCTTGTC

CAGAGTATAAAACGAACAGAATCCAGCGCGGCTCTACATCCATTACTGCAAGAGATTTACAGAGACATGT

ATTGATGATGTTGAAAATAAGAACCTTATAAAAGGCGATTATTCCTTCAAAGCTATATATGTCCACTCAT

AATATTCAGTTTGACTATATCTTGTTTTATAAACAATAGACTTAATGTATAGTAATTGCTTTTCTCAATA

TCATCAGATTTGCATTTCAGTTTAACACTAACTTATAAGGTTAACCTTAACAATCGCTTCTAAAAATAAA

TCTAAAACATATTTTTGTAAAGAATACAATTGTACAAGCATTTCCATTATAGTATGTATAATTTATG

**Primer verification**

**>Thermal gradient for optimum T_A_**

62.0°C to 52.0°C

**>Standard Curve Parameters**

**Slope:** -3.15

**R^2^:** 0.991

**E%:** 107.6%

**cDNA Dilution Factor:** 1:2

**[cDNA] range:** 10.0 ng – 0.6 ng

**T_A_:** 52.0°C

**Final [primer pair]:** 2.0 uL/rxn(i.e., 250 nM per primer)

**>Melt Curve**

Mean T_m_ (SEM): 84.13°C (0.02)


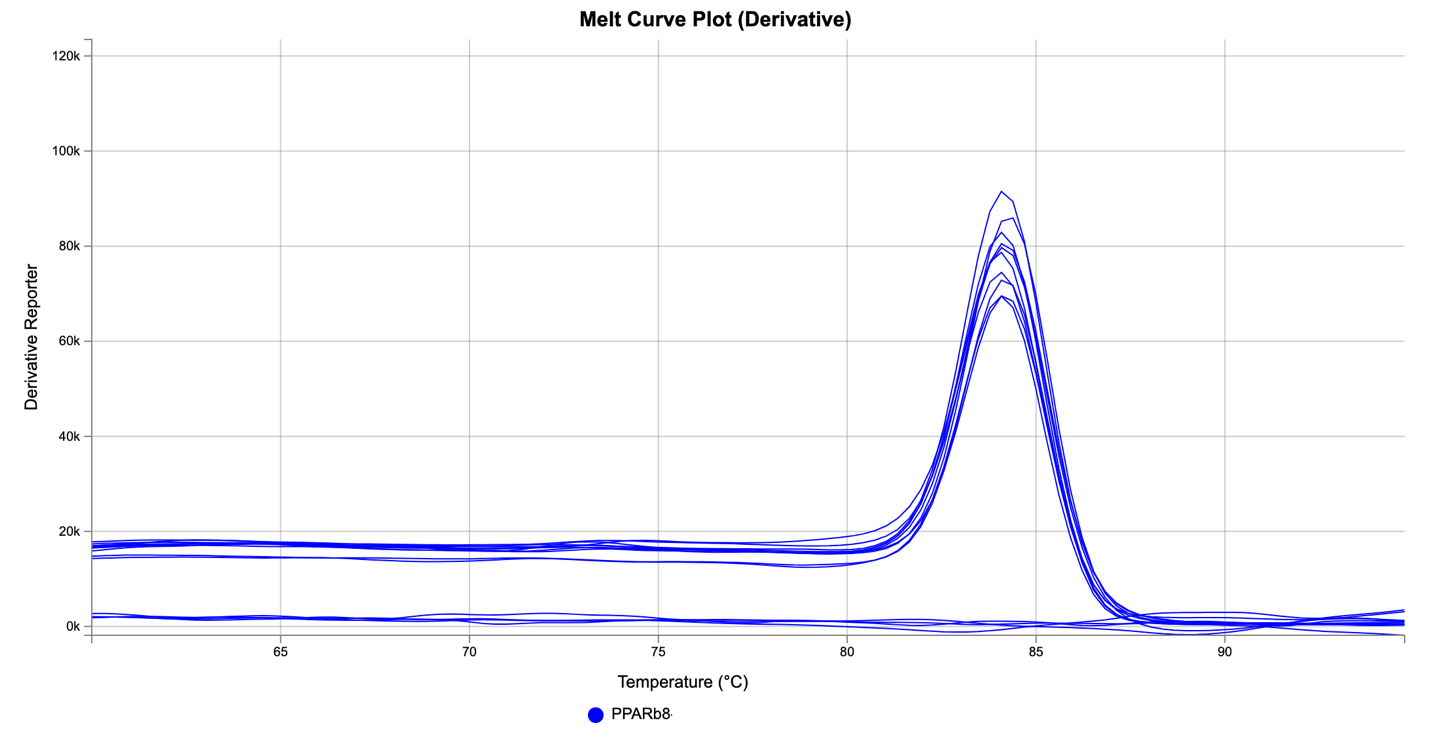


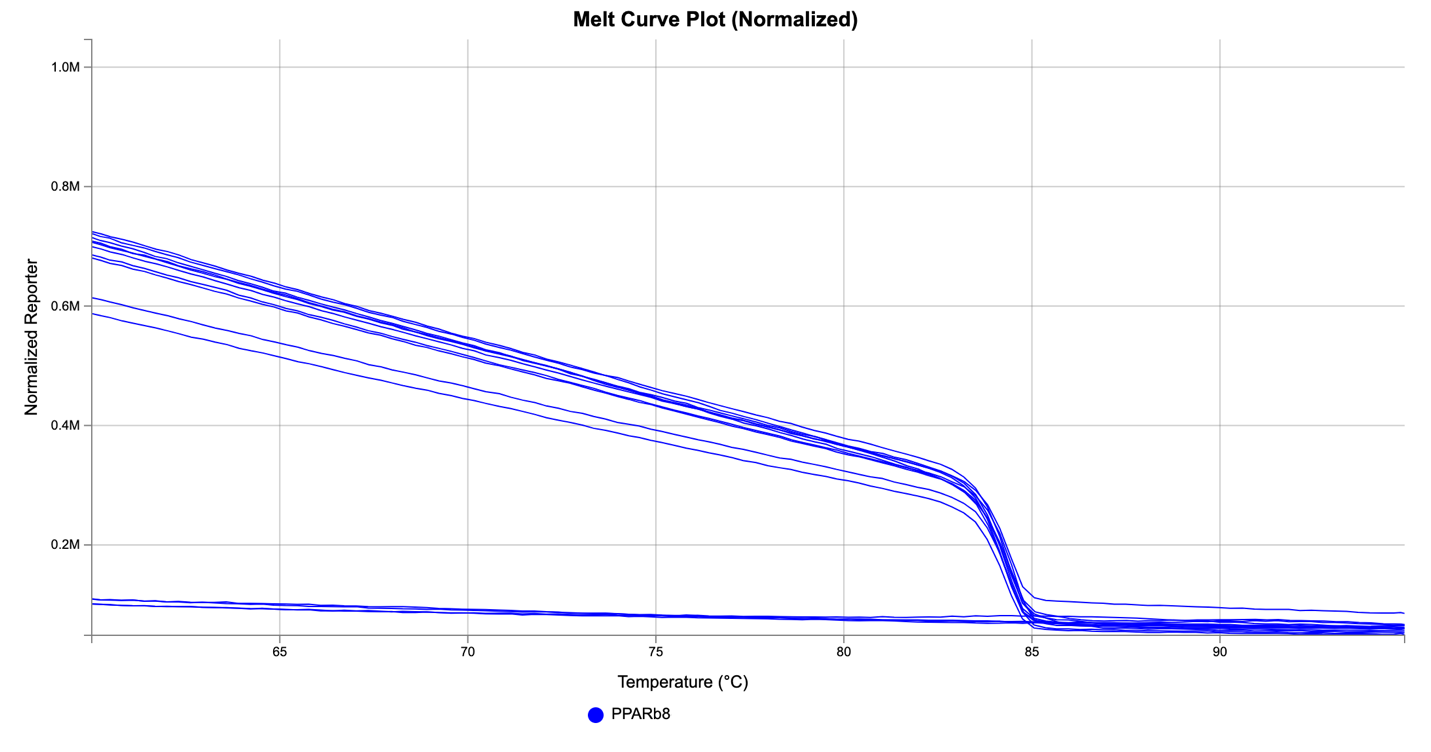


# **Text 10.** *xPPARg* primer documentation

**xPPARg**, peroxisome proliferator activated receptor gamma L homeolog (*pparg.L*): target species—African Clawed Frog (*Xenopus laevis*)

**F/Left Primer (5^’^-3^’^):** CGTCACCCCCTTACTTTTCA

20 bp Tm 54.7°C 50.0%GC

**R/Right Primer(5^’^-3^’^**): CTTCTTGTGAATCCGGCAAT

**In sequence:** ATTGCCGGATTCACAAGAAG

20 bp Tm 53.3°C 45.0%GC

**Source:** Self-designed

**Spans exon-exon:** Yes

**Amplicon Product:** 222 bp

Start & End of reading frame

Predicted F primer sequence

Predicted R Primer sequence

>NM_001087843.1 Xenopus laevis peroxisome proliferator activated receptor gamma L homeolog (pparg.L), mRNA

GAGGCCGAGGAACAAGTGCAGCATTTACTACTCCCTGCTACTCCCTGCTACTCCCTGCTACTCCCTGCAC

AGCAACTTACTGCTACTCCCAGCAGAGCCAACAACTCCCTGCTACTCCCAGCAGAGCCAACAACTCCCTG

CTACTCCCTGCACAGCCAACAACTCCCTGCTACTCCCTGCACAGCCAACAACTCCCTGCAGGACGGGCAC

AGTGACTGGGCAAATCCACAAAGCGCAGGGGGGCTGGCAGTTGGGCACCGCAGGTCTGAGGGTTAATAAT

CTCCCAACTCCGGTCAGATCCGTCTCCCGGCAGAGGTGGCACATGGGAGTCTGAGCGCGTCGGAATCGCA

GCAACAAGAAGCACTTTGTGACATTCCAGGAGAGGAGCAGGAGCGAGCACAGTCATGGTGGACACTGAAA

TGCCTTTCTGGTCCAATTTGAATTTCGGAATGAATTCCATGGACATGTCGGCTCTGGAGGACCATTGCCA

GCCCTATGATATTAAGCCCTTTACTACTGTAGACTTTTCCAGCATCAATTCTCACTATGATGATATTCTG

GATGAAAAGACTTTCCTCTGCAGGAACGACCAGTCGCCCATTGATTATAAATATGACCTGAAGCTACAGG

AATGCCAAAGTTCCATAAAGCTGGAGCCCCCGTCACCCCCTTACTTTTCAGACAAACCACAGTGTAGTAA

AGCCTTTGAAGACACCCCAAATTCCTTCATAGCCATTGAATGCAGAGTTTGTGGGGACAAAGCCTCTGGG

TTCCACTATGGCGTCCATGCATGTGAAGGTTGCAAGGGGTTCTTTAGAAGAACAATCAGGTTGAAGTTAA

TTTATGAAAGGTGCGACCTGAATTGCCGGATTCACAAGAAGAGCAGAAACAAATGTCAGTTCTGCCGGTT

CCAGAAGTGCCTGGCGGTCGGCATGTCCCATAATGCCATCAGGTTCGGTCGGATGCCGCAGGCAGAGAAG

GAGAAATTATTGGCTGAAATCTCCAGCGACATCGACCAGTTGAACCCCGAGTCGGCCGATCAGCGAGTCC

TGGCCAAACACTTGTACGACTCCTACGTCAAGTCCTTCCCACTGACCAAAGCCAAGGCCCCGGGCCATCC

TGACGGGCAGAGCCACCGACAAAACTCCCGTGGTTATACACGACATGAACTCGCTGATGATGGGGGAGGA

TCAGATCAAGGGGCAGTGCGTGAGCCCCGAGCAGAACAAGGAGGTGGCGATTCGAATCTTCCAGCGCTGT

CAGTCGCGCTCCGCGGAGGCGTTCGGGAAATCACGGAATTTGCCAAGAACATCCCGGGATTCGTCAGCCT

CGACCTGAACGACCAAGTGACGCTGCTGAAATACGGGGTGCACGAGATCATATTCACTATGCTGGCCTCG

CTCATGAACAAAGACGGGGTGCTCGTAGCCGAGGGCCAAGGCTTCATGACCCGCGAGTTTCTCAAGAGTC

TGCGCAAACCCTTCTCCGACTTCATGGAGCCCAAATTCGAATTCGCCATTCGCTTCAACTCCCTCGAACT

CGACGACAGCGACTTGGCCATATTTGTAGCGGTCATTATACTGAGCGGAGACCGGCCGGGGCTGCTGAAC

GTGAAACCCATAGAAGACATTCAGGACAGTCTGTTACAGGCGCTGGAGCTGCAGCTGAAGCTCAATCATC

CAGACTCCGCCCAGCTCTTCGCTAAACTGCTGCAGAAGATGACGGACCTGCGGCAGGTGGTGACTGAGCA

CGTGCAGTTACTGCAGCTCATTAAGAAGACGGAGGCCGACATGTGCCTGCACCCCCTCTTACAGGAGATC

TACAAAGACTTGTACTGAGCCCCCCATTATCCACAGACACACTCACCCCCATTTGCACTATTTCTACGTG

GGACCCAACAAAGAAACTGCAGGGCCCCCCATTGCGCCTCATCGGCAGCTCAGGATTTTGACTCTCACAG

ACTTGATCCGCCTCCCGGGGGGGGGCCACAATACTCAAATGTTCCACTTTCTACAAATAATTGTCCCCCT

TAAATGTCTTGTTATT

**Primer verification**

**>Thermal gradient for optimum T_A_**

58.0°C to 48.0°C

**>Standard Curve Parameters**

**Slope:** -3.49

**R^2^:** 0.983

**E%:** 93.3%

**cDNA Dilution Factor:** 1:2

**[cDNA] range:** 5.0 ng – 0.3 ng

**T_A_:** 48.7°C

**Final [primer pair]:** 1.0 uL/rxn(i.e., 125 nM per primer)

**>Melt Curve**

Mean T_m_ (SEM): 83.37°C (0.02)


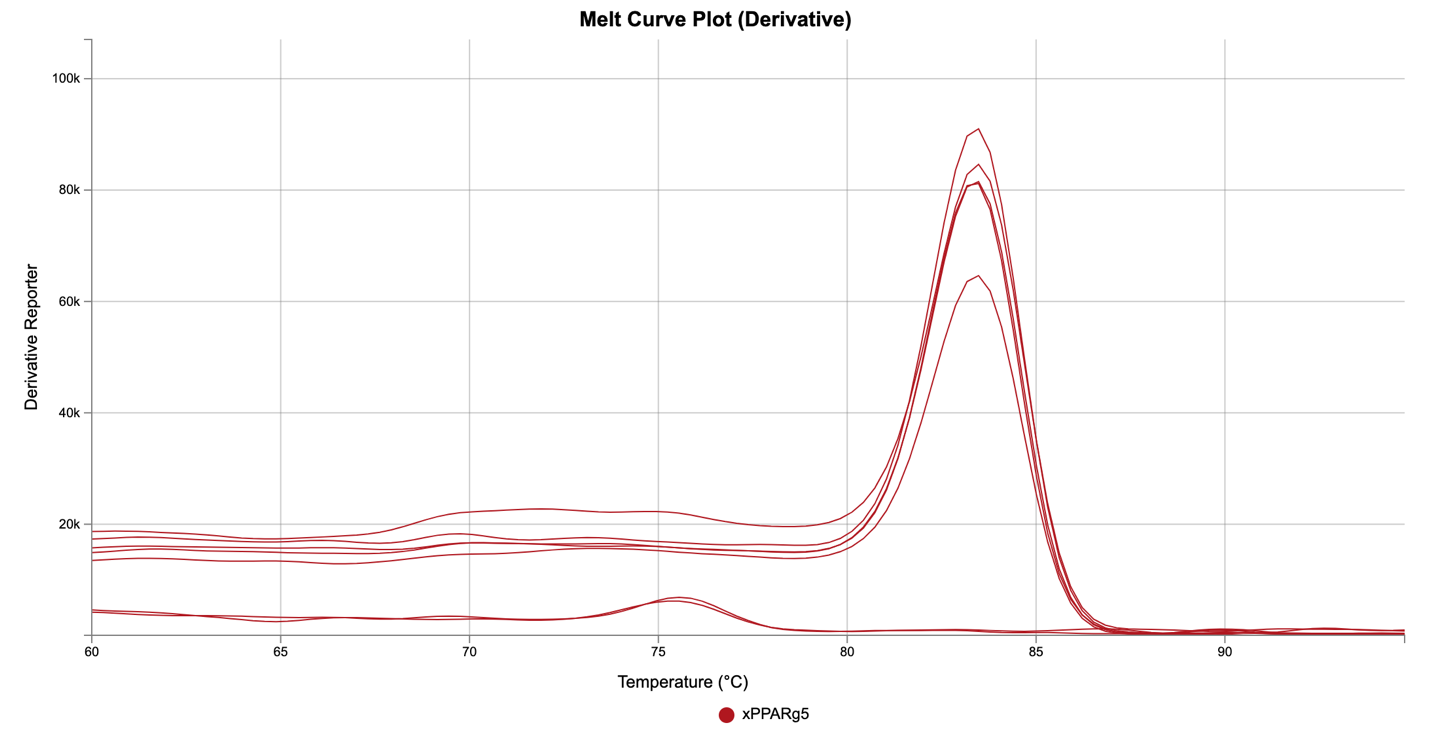


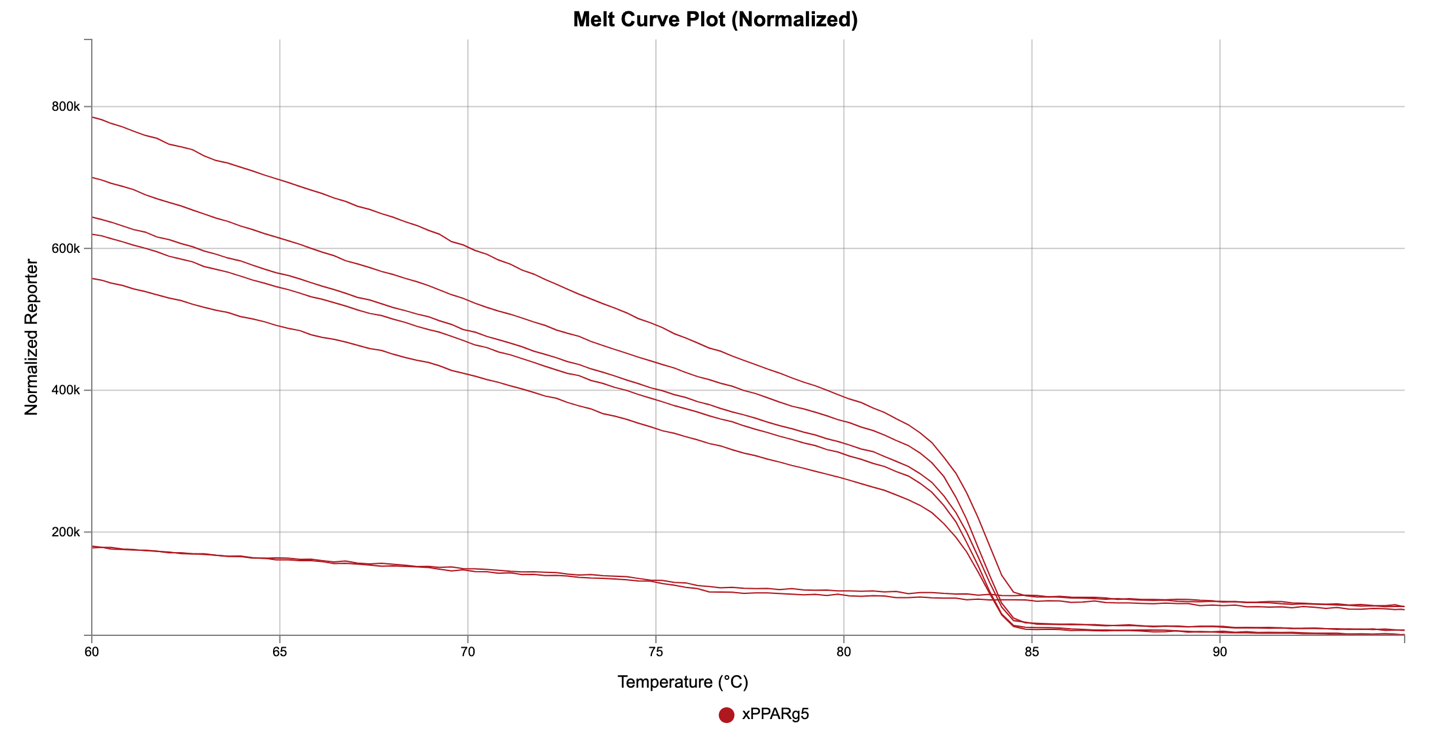

Supplement: Supplementary file 2 — Supplementary_Information_Primer_FINAL. [file JEZ-343-1191-s001.docx]
